# Synthesis, Characterization, and the Effect of Lewis Bases on the Nuclearity of Iron Alkoxide Complexes

**DOI:** 10.1021/acs.inorgchem.3c04538

**Published:** 2024-04-12

**Authors:** Reilly K. Gwinn, Matthew Williams, Trevor P. Latendresse, Carla Slebodnick, Diego Troya, Tasnema Tarannum, Diana A. Thornton

**Affiliations:** † Department of Chemistry, Virginia Polytechnic Institute and State University, Blacksburg, Virginia 24061, United States; ‡ Department of Chemistry and Chemical Biology, Harvard University, Cambridge, Massachusetts 02138, United States

## Abstract

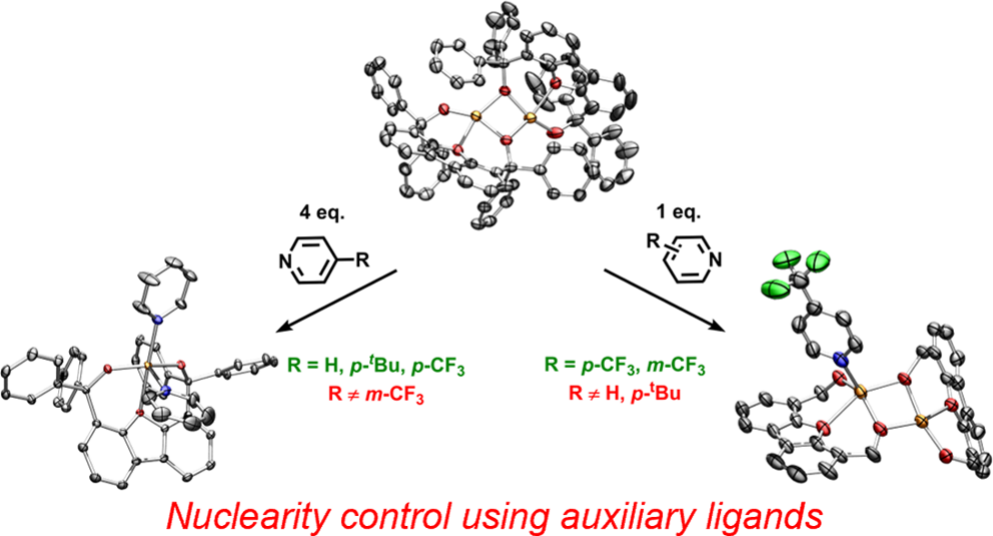

Inspired by the potential of alkoxides as weak-field ligands and their ability to bridge, we report herein a series of high-spin iron complexes supported by a bis-alkoxide framework ^**Ph**^**Dbf**. A diiron complex [Fe_2_(^Ph^Dbf)_2_] (**1a**) is obtained upon metalation of the ligand, whereas addition of substituted pyridines affords five-coordinate mononuclear iron complexes [(R-Py)_2_Fe(^Ph^Dbf)] (**2a**–**4a**, R = H, *p*-^*t*^Bu, *p*-CF_3_). The potential for nuclearity control of the metal complexes via auxiliary ligands is highlighted by the formation of asymmetric diiron species [(*p*-CF_3_–Py)Fe_2_(^Ph^Dbf)_2_] (**5a**) and [(*m*-CF_3_–Py)Fe_2_(^Ph^Dbf)_2_] (**6a**) with trifluoromethyl substituted pyridines, while electron-rich pyridines only produced monomeric species. Electronic properties analysis via UV–vis, electron paramagnetic resonance, ^57^Fe Mössbauer spectroscopy, and time-dependent density functional theory, along with redox capabilities of these complexes are reported to illustrate the effect of nuclearity on reactivity and the potential of these complexes to access higher oxidation states relevant in oxidative chemistry. Species **1a**–**5a**, [(THF)_2_Fe(^Ph^Dbf)][PF_6_] (**7**), [PyFe(^Ph^Dbf)Cl] (**2b**), and [Py_2_Fe(^Ph^Dbf)][PF_6_] (**2c**) were characterized via SCXRD. Indirect evidence for the formation of dimeric Fe(III) species (**1b**, **5b**, and **6b**) is discussed.

## Abstract 2

The synthesis, characterization, and reactivity of dimeric and monomeric iron alkoxide complexes are reported. Studies revealed an effect of Lewis bases on nuclearity, with electron-rich pyridines promoting formation of pyridine-bound monomeric iron species exclusively, whereas 4-trifluoromethylpyridine providing access to both a monomeric and a dimeric pyridine-bound complex. Alternatively, 3-trifluoromethylpyridine is proposed to afford only the respective dimer species, highlighting the diverse coordination profile of these iron alkoxide complexes as a function of pyridine substitution.

## Introduction

Direct C–H bond functionalization has attracted much interest over the past decades given the potential applications to industrial and pharmaceutical processes, for example. Recent efforts have focused on elaborating more sustainable ways for carrying out such transformations, with an emphasis on utilizing earth-abundant, late first-row transition metals.^1,2^ As these metals favor one-electron chemistry, most first-row transition metals activate C–H bonds through the intermediacy of a high-valent species featuring metal–ligand multiple bonds which is often involved in a stepwise C–H bond functionalization event.^2^ Numerous methodologies have explored such an approach and studies have shown that complexes adopting high-spin electronic configurations^2−5^ are more likely to promote the desired C–H bond functionalization owing to the decreased stability and, thus, increased reactivity of the metal–ligand multiply bonded unit.

To access these high-spin metal complexes, various weak-field ligands have been explored, with a focus primarily on N-based scaffolds such as dipyrromethenes^5−7^ or β-diketiminates,^8−10^ which have demonstrated rich and unique chemistry. Fewer studies have examined weaker-field alkoxides as supporting ligands for late, first-row transition metals, primarily due to the propensity of alkoxides to form cluster compounds^11,12^ if not carefully tuned. Nevertheless, several groups have made use of either fluorinated alkoxides to minimize the π-donicity of the ligand and thus inhibit clustering^13−19^ or sterically encumbering alkoxides to enable the formation of well-defined mononuclear complexes.^12,20−25^ These investigations have revealed that alkoxide complexes can accommodate unique electronic configurations (e.g., high-spin square planar complexes^18,26−29^) and indeed allow access to high-spin metal–ligand multiply bonded intermediates (e.g., an iron–oxo example^21^ as well as iron–imido complexes^30−33^ not directly detected, but proposed based on reactivity and computational support).

In addition to the supporting ligand, auxiliary ligands can also have an impact on the electronic structure, nuclearity, and reactivity of metal complexes.^34,35^ For example, addition of pyridine to an intermediate spin ferric imido was proposed to access a high-spin iron imido that can promote hydrogen-atom abstraction from weak C–H bonds.^9^ Addition of Lewis bases can often help promote the formation of well-defined monomeric complexes when aggregation or disproportionation is otherwise favored.^36^ Alternatively, auxiliary ligands can introduce steric constraints to alter product selectivity.^37,38^ Being able to systematically tune structural, electronic, and reactivity properties of metal complexes using auxiliary ligands can be beneficial for synthetic ease as well as for the possibility of accessing a wider reactivity scope.

With an interest in expanding and tuning the reactivity of late transition metal alkoxides, we have been interested in examining bis-alkoxides as ligands for supporting both mono- and bimetallic complexes and understanding the properties such ligands impart. We report herein a series of dibenzofuran-supported bis-alkoxide iron complexes, their distinct behavior in the presence of Lewis bases, and their redox capabilities. All complexes display high-spin electronic configurations, regardless of the auxiliary ligand. We have found that a diiron complex is favored in the absence of strong Lewis bases, albeit addition of some substituted pyridines leads to isolation of 5-coordinate pyridine-bound mononuclear iron species. Interestingly, addition of a weaker Lewis base such as 4-trifluoromethylpyridine allows for isolation of both a monomeric iron complex as well as an asymmetric dimer where only one of the iron centers binds the respective pyridine. Similarly, exposure of the diiron complex to 3-trifluoromethylpyridine is proposed to form an analogous asymmetric dimer; however, no mononuclear iron complex can be obtained. These simple differences in coordination chemistry are of interest as they could allow for systematic tuning of the reactivity of these iron complexes toward the desired transformation.

## Results and Discussion

### Synthesis of Iron Complexes

**Figure 1:**
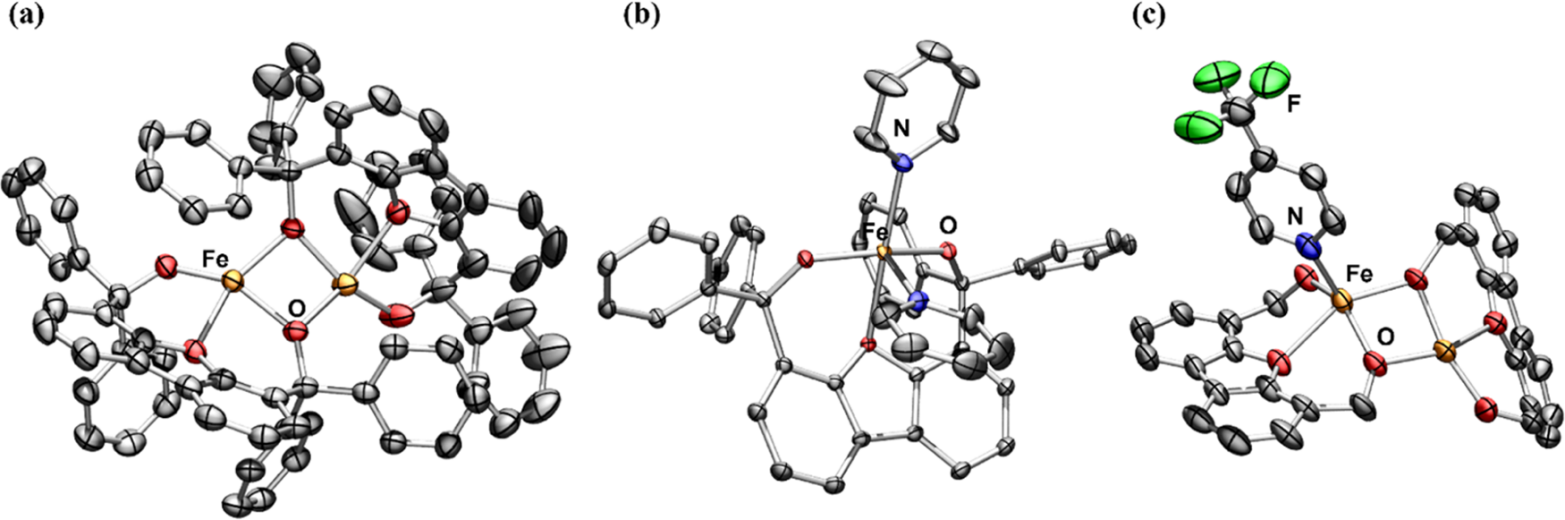
Solid-state molecular structures for (a) [Fe_2_(^Ph^Dbf)_2_] (**1a**), (b) [Py_2_Fe(^Ph^Dbf)] (**2a**), and (c) [(*p*-CF_3_–Py)Fe_2_(^Ph^Dbf)_2_] (**5a**) (truncated) with anisotropic displacement ellipsoids at 50% probability level. Color scheme: Fe, orange; N, blue; O, red F, green. Hydrogens, solvent molecules, disordered benzene groups in **1a**, and phenyl groups on ligand in **5a** are omitted for clarity.

**Scheme 1:**
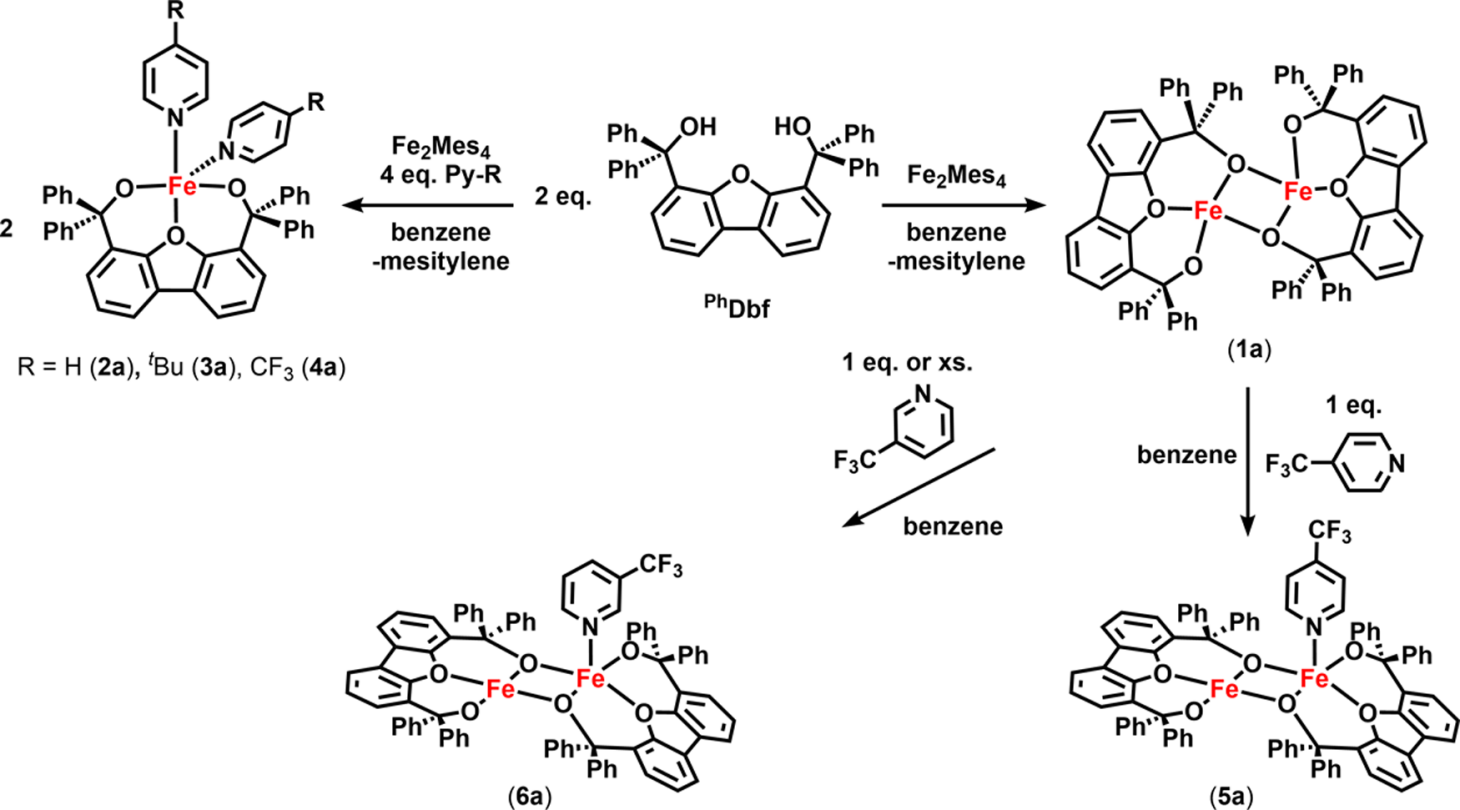
Synthesis of Iron Alkoxide Complexes with and without Lewis Bases

The dibenzofuran-supported bis-alkoxide scaffold 4,6-bis(hydroxydiphenylmethyl)dibenzofuran (^**Ph**^**Dbf**) was synthesized as previously described.^39^ Addition of a stoichiometric amount of tetramesityldiiron (Fe_2_Mes_4_) to ^**Ph**^**Dbf** in benzene resulted in immediate consumption of both materials and formation of a new paramagnetic species, **1a**, along with generation of mesitylene as indicated by ^1^H NMR (Scheme 1). Zero-field ^57^Fe Mössbauer characterization (90 K) of **1a** revealed one major iron-containing species with parameters (δ = 0.92 mm/s and |Δ*E*_Q_| = 0.78 mm/s, Figure S7) that are consistent with high-spin ferrous centers, alongside an additional signal (δ = 1.12 mm/s and |Δ*E*_Q_| = 2.11 mm/s, Figure S7), which is likely a result of decomposition based on the broadness of the signal. Slow evaporation of an ether solution of the resulting pale-gray solid **1a** afforded colorless X-ray quality crystals which allowed identification of **1a** as a diiron alkoxide complex [Fe_2_(^Ph^Dbf)_2_] (Figure 1a), with each of the ^**Ph**^**Dbf** ligands involved in one bridging interaction. Both the weak-field nature of the alkoxide ligands and their propensity for clustering are likely contributing to the formation of a dimeric structure. Analysis of the X-ray structure of **1a** shows differences between the metal coordination environments, albeit both iron centers have a distorted square planar geometry. The slight perturbations in bond metrics and angles with respect to each iron (Table S9) could be an artifact of packing during crystallization or could be a result of the steric profile of the ligand, causing additional tilting of one of the ^**Ph**^**Dbf** ligands, which can be observed in a space-filling model of **1a** (Figure S73). Unlike the rare high-spin square planar iron complexes previously reported,^26,27,29^ the distorted square planar geometry of **1a** likely decreases the gap between the d-orbitals, enabling easy access to a high-spin configuration. Further, the distances between the central furan O-atom and the iron centers of 2.2230(13) and 2.2243(16) Å are indicative of minimal donation from the ligand to the metal, with calculated Mayer bond orders lower than 0.13 (B3LYP/def2-TZVP level). Although the weak interaction could be a consequence of the ligand geometry, we have noted that the diiron complex remains unchanged if exposed to ethereal solvents (e.g., diethyl ether, tetrahydrofuran) upon synthesis or if prepared in tetrahydrofuran, which is unlike typical electrophilic metal centers that quickly coordinate Lewis bases when exposed to them. Nevertheless, upon addition of four equivalents of pyridine to **1a**, a bright orange solution results immediately and a new species (**2a**) is detected in the ^1^H NMR spectrum. Orange crystals of **2a** were obtained from a benzene solution of **2a** at room temperature and X-ray crystallographic characterization confirmed its identity as a monomeric iron species (Figure 1b) with two molecules of pyridine coordinated to the metal center [Py_2_Fe(^Ph^Dbf)]. **2a** can be alternatively prepared directly from Fe_2_Mes_4_ using stoichiometric amounts of pyridine and ^**Ph**^**Dbf** (Scheme 1). Although both methods afford **2a** as the main product, the direct synthetic route generates overall cleaner **2a** following workup. Using this protocol, we set out to prepare a series of monomeric pyridine-bound iron complexes using both electron-rich and electron-poor pyridine ligands: 4-*tert*-butylpyridine (**3a**), and 4-trifluoromethylpyridine (**4a**) (Scheme 1).

Interestingly, we noted that although **3a** behaved very similarly to the parent pyridine-bound complex, addition of a fluorinated pyridine into the system altered the outcome. If less than four equivalents (relative to Fe_2_Mes_4_) of 4-trifluoromethylpyridine were accidentally used, a mixture of two fluorine-containing species is noted (^19^F δ: −120.1 and −141.5 ppm). Diffusion of hexanes into a frozen benzene solution of the mixture resulting from the above reaction of Fe_2_Mes_4_ with less than four equivalents of 4-trifluoromethylpyridine at −35 °C afforded X-ray quality purple crystals, which identified a dimeric iron complex with only one iron center binding 4-trifluoromethylpyridine ([(*p*-CF_3_–Py)Fe_2_(^Ph^Dbf)_2_], **5a**, Figures 1c and S77). ^19^F NMR analysis of the crystals indicated that the signal observed at −120.1 ppm corresponds to **5a**, which can be further confirmed by directly forming **5a** upon addition of one equivalent of 4-trifluoromethylpyridine to **1a** (Scheme 1). In light of this observation, we proposed that the other fluorine-containing species noted in the ^19^F NMR spectrum is likely the corresponding mononuclear complex. Indeed, exposing **1a** to four equivalents of 4-trifluoromethylpyridine provides **4a** (^19^F δ: −141.5 ppm) as judged by both ^1^H and ^19^F NMR spectroscopies. Recrystallization via diffusion of hexanes into a frozen benzene solution of **4a** at −35 °C afforded purple crystals and solidified the assignment of **4a** as the monomeric iron complex [(*p*-CF_3_–Py)_2_Fe(^Ph^Dbf)] (Figure S76).

Unlike 4-trifluoromethylpyridine, addition of 3-trifluoromethylpyridine to **1a** or directly to Fe_2_Mes_4_ afforded a single species (**6a**) by ^19^F NMR, regardless of the amount of Lewis base used. To probe the identity of **6a**, we turned our attention to ^1^H NMR and examined the spectra of our isolated complexes to establish trends. Comparing the spectrum of **4a** (Figure S17) with that of the other monomeric complexes synthesized (**2a** and **3a**, Figures S15 and S16) revealed a similar pattern in the chemical shifts, with perturbations likely caused by the different pyridine coordinated to the iron center. Alternatively, the ^1^H NMR spectrum of **5a** displays a larger number of resonances (Figure S19), consistent with an asymmetric molecule, along with signals in the negative chemical shift region, unlike any of the other characterized iron complexes. Using these notable differences and comparing the ^1^H NMR spectra of **2a**–**4a** and **5a** with that of **6a** (Figure S19), we propose that **6a** is likely a dimeric species akin to **5a** (Scheme 1). Taking advantage of this ^1^H NMR handle to assess the extent of pyridine coordination to the iron center and the observed differences between the two fluorinated pyridine substrates, we tested whether the respective diiron species with only one Lewis base coordinated can be accessed with pyridine and 4-*tert*-butylpyridine. Nevertheless, **2a** and **3a**, respectively, were exclusively obtained regardless of the method of preparation and the amount of pyridine added (one or four equivalents). Further, we noted that addition of pyridine or 4-*tert*-butylpyridine to **6a** results in the formation of the corresponding **2a** or **3a** (Figures S87 and S88), and addition of 4-trifluoromethylpyridine results in a mixture of **4a** and **5a** (Figure S89). This exchange is also observed for **2a** and **4a** (Figures S81, S82, S85, and S86), while **3a** does not display this lability of the bound 4-*tert*-butylpyridine (Figures S83 and S84). These studies suggest that pyridine coordination to the iron center may be correlated with the electron donicity of the pyridine ligand, which could be the reason for the formation of the observed dimeric complexes. Although it is unclear without further investigations why no monomeric species can be accessed with 3-trifluoromethylpyridine, the difference in the steric profile of the *meta*- vs *para*-substituted pyridine ligands may also play a role. Similarly, we have noted that addition of the more sterically hindered lutidine affords a relatively messy ^1^H NMR spectrum (Figure S90) with features analogous to those of **5a** and **6a**, which could also point toward the contribution of steric factors to pyridine coordination, albeit a systematic study is necessary to verify these effects. Nonetheless, these results are interesting as they suggest a dependence of the structure of the iron complexes on the identity of the pyridine ligand. This flexibility in structural control could allow for fine-tuning the reactivity of these complexes by carefully controlling nuclearity and coordination environment.

### X-ray Crystallographic Analysis of **2a**–**4a**

Akin to **1**, the X-ray structures of **2a**–**4a** reveal weak coordination of the iron center to the furan O-atom, with Fe–O distance being on the order of 2.3 Å. Indeed, the calculated Mayer bond order between the O-atom of the furan ring and the Fe center is only 0.10, significantly smaller than that for the other two atoms of the ^Ph^Dbf linker (0.59). Accounting for this weak interaction allows us to describe the geometry of **2a**–**4a** as square pyramidal, with the square plane defined by the three O-atoms and N-atom of one pyridine ligand, with the second coordinated pyridine occupying the axial position. Alternatively, leaving out the Fe–O_furan_ interaction gives a seesaw geometry at the iron center, similar to a previously reported chelating bis-alkoxide bis-tetrahydrofuran iron complex (Fe[OO]^Ph^(THF)_2_).^31^ The different unit linking the two alkoxides in **2a**–**4a** versus Fe[OO]^Ph^(THF)_2_ results in distinct steric profiles imposed by the alkoxide substituents, with ^**Ph**^**Dbf** encouraging a more exposed iron center. Although no major differences in the bond metrics of the three structures can be noted (Table S1), the O_furan_–Fe–N angle is significantly closer to 180° in **2a** as compared to **3a** and **4a**, respectively (**2a**: 176.34(4)°; **3a**: 169.93(4)°; **4a**: 169.84(6)°). Similarly, the N–Fe–N angle in **2a** (94.48(4)°) is significantly lower than the corresponding angle in **3a**–**4a** (**3a**: 102.35(4)°; **4a**: 104.19(6)°). Among representative bond lengths, the average Fe–O_alkoxide_ and Fe–N distances are 1.91 Å (range 1.9082(9)–1.915(8) Å) and 2.15 Å (range 2.1304(11)–2.1618(17) Å). As pyridine ligands can be redox active,^40,41^ we took a closer look at the structural parameters of each coordinated pyridine in **2a**–**4a**, albeit no indication for a radical anion character could be found. The C–N and C–C bond lengths agree well with typical values for neutral pyridine ligands^40^ (Table S2), thus arguing against redox noninnocence in the present complexes.

### Characterization of the Electronic Properties of **1a**–**6a**

**Figure 2:**
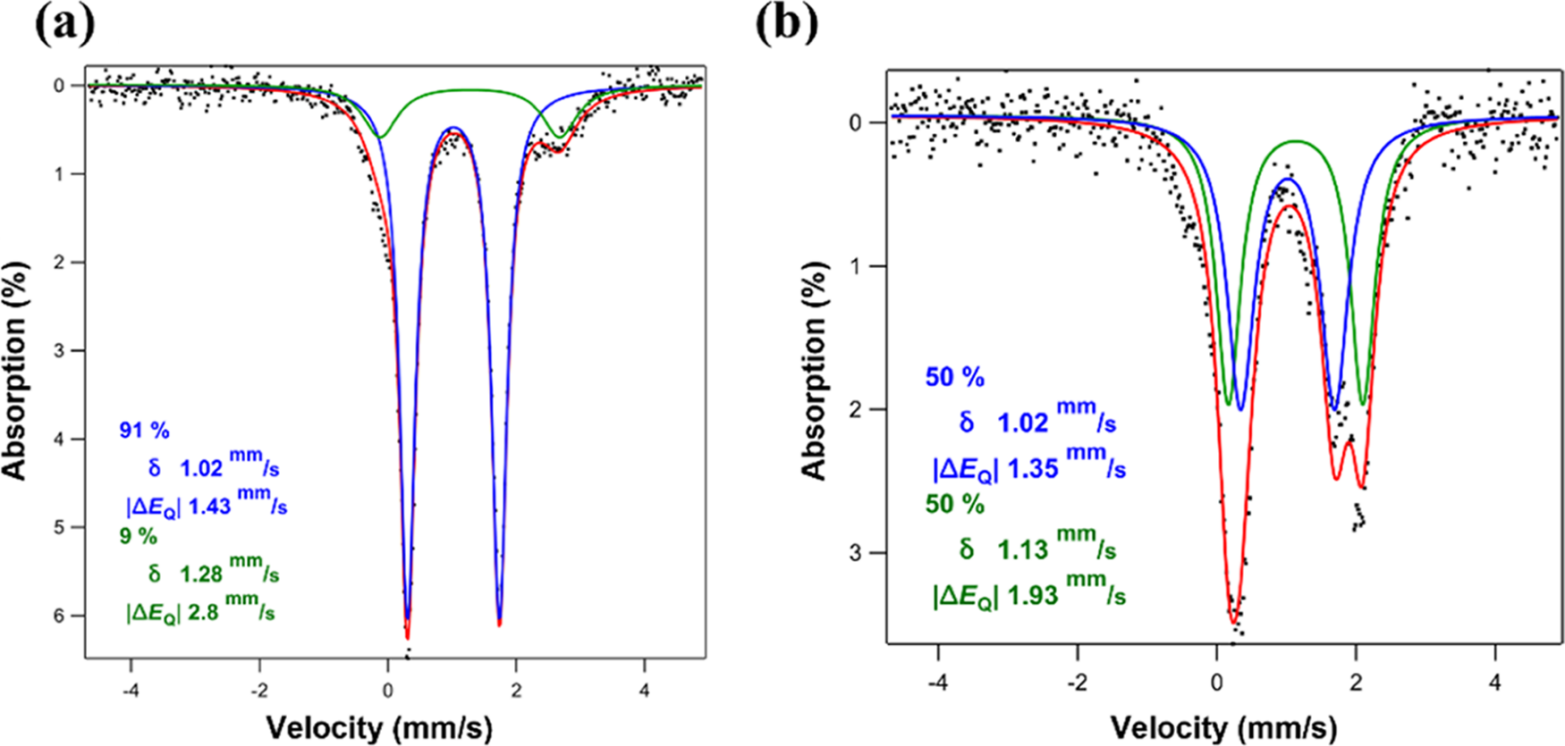
(a) Zero-field ^57^Fe Mössbauer spectrum for [(Py)_2_Fe(^Ph^Dbf)] (**2a**) collected at 90 K. (b) Zero-field ^57^Fe Mössbauer spectrum for [(*m*-CF_3_–Py)Fe_2_(^Ph^Dbf)_2_] (**6a**) collected at 90 K.

Zero-field ^57^Fe Mössbauer spectroscopy was employed to assess the spin states of the isolated complexes. All spectra reveal one major quadrupole doublet with an isomer shift value of 1.02, 1.01, and 0.99 mm/s and quadrupole splittings of 1.43, 1.5, and 1.24 mm/s for **2a**, **3a**, and **4a** (Figures 2a and S8–S10), respectively, parameters which are consistent with four-coordinate high-spin ferrous centers.^42^ The higher isomer shift and quadrupole splitting values as compared to those observed for **1a** can be attributed to a higher coordination number (five versus four) and a more asymmetric coordination environment around the iron center for the monomeric species. As further support for the identity of **6a** as a dimeric pyridine-bound species, two distinct quadrupole doublets can be found (50% δ: 1.02 mm/s and |Δ*E*_Q_| = 1.35 mm/s and 50% δ: 1.13 mm/s and |Δ*E*_Q_| = 1.93 mm/s, Figure 2b), reflecting still the quintet spin state at each iron center. Based on the quadrupole splitting magnitudes, we propose that the lower quadrupole splitting value corresponds to the pyridine-free iron center. The quintet spin states for all complexes can be additionally gleaned from the magnetic moment values of 4.67, 4.88, 5.03, and 6.76 μ_B_ for **2a**–**5a** which are all close to the spin-only value of 4.90 μ_B_ expected for one *S* = 2 center and 6.93 μ_B_ predicted for two noninteracting high-spin ferrous centers, respectively.

**Figure 3:**
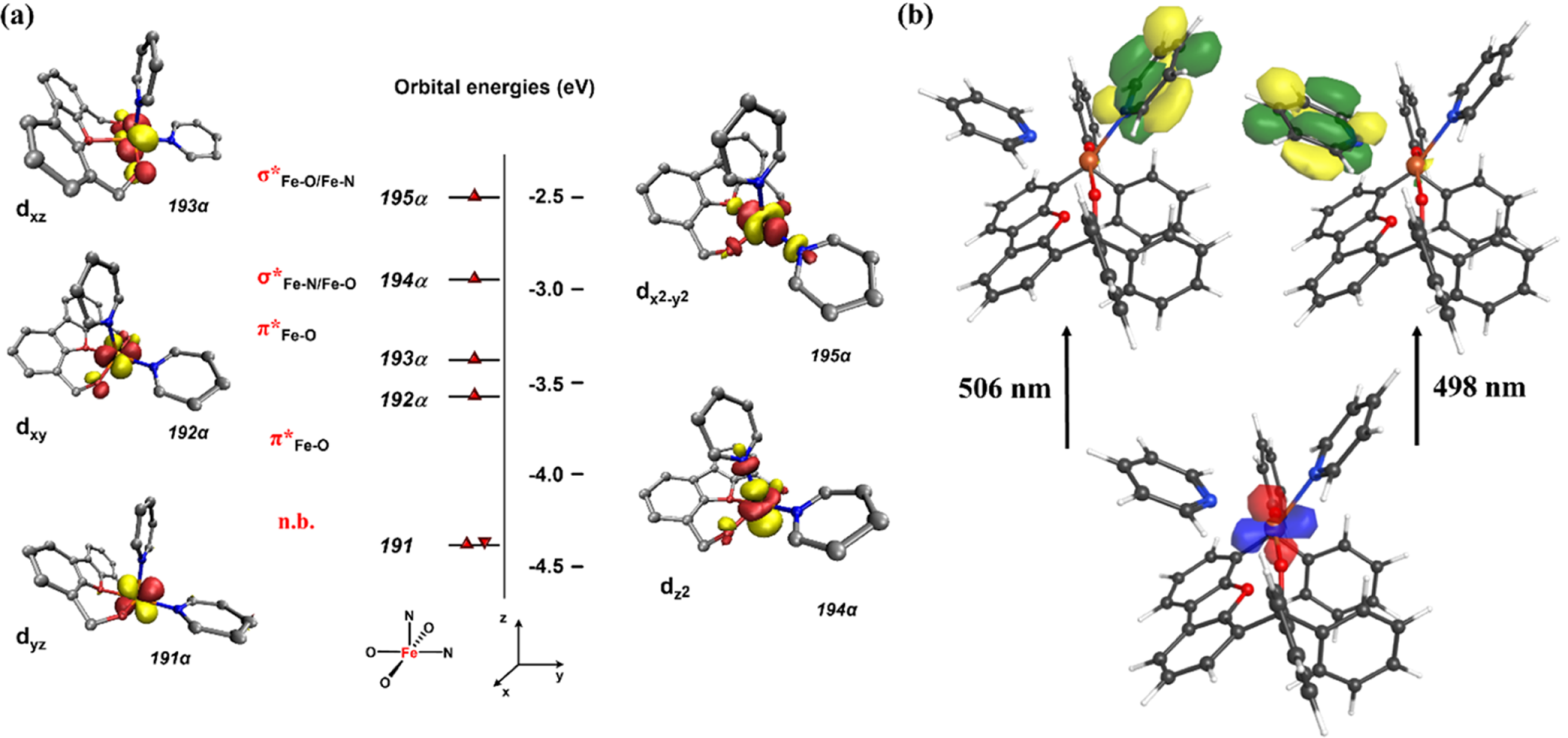
(a) Frontier molecular orbitals for [(Py)_2_Fe(^Ph^Dbf)] (**2a**) (isovalue 0.04). Quasi-restricted orbitals (α-spins) are shown; (b) .

Experimental results were further supported with unrestricted, single-point calculations, which provided ^57^Fe Mössbauer isomer shift parameters in agreement with those observed experimentally. The computed electronic structure for the monomeric iron species provides a qualitative picture of the frontier orbitals composed primarily of the five *d*-orbitals: *d*_*yz*_ (nb) < *d*_*xy*_ (π*_Fe–O_) < *d*_*xz*_ (π*_Fe–O_) < *d*_z^2^_ (σ*_Fe–O, Fe–py_) < *d*_*x*^2^–*y*^2^_ (σ*_Fe–O, Fe–py_) (Figure 3a for **2a**), in line with the observed geometry of the iron complexes. The spin density plot shows minimal involvement from the pyridine ligands (Figure S69), further corroborating the neutral character of these ligands.

**Figure 4:**
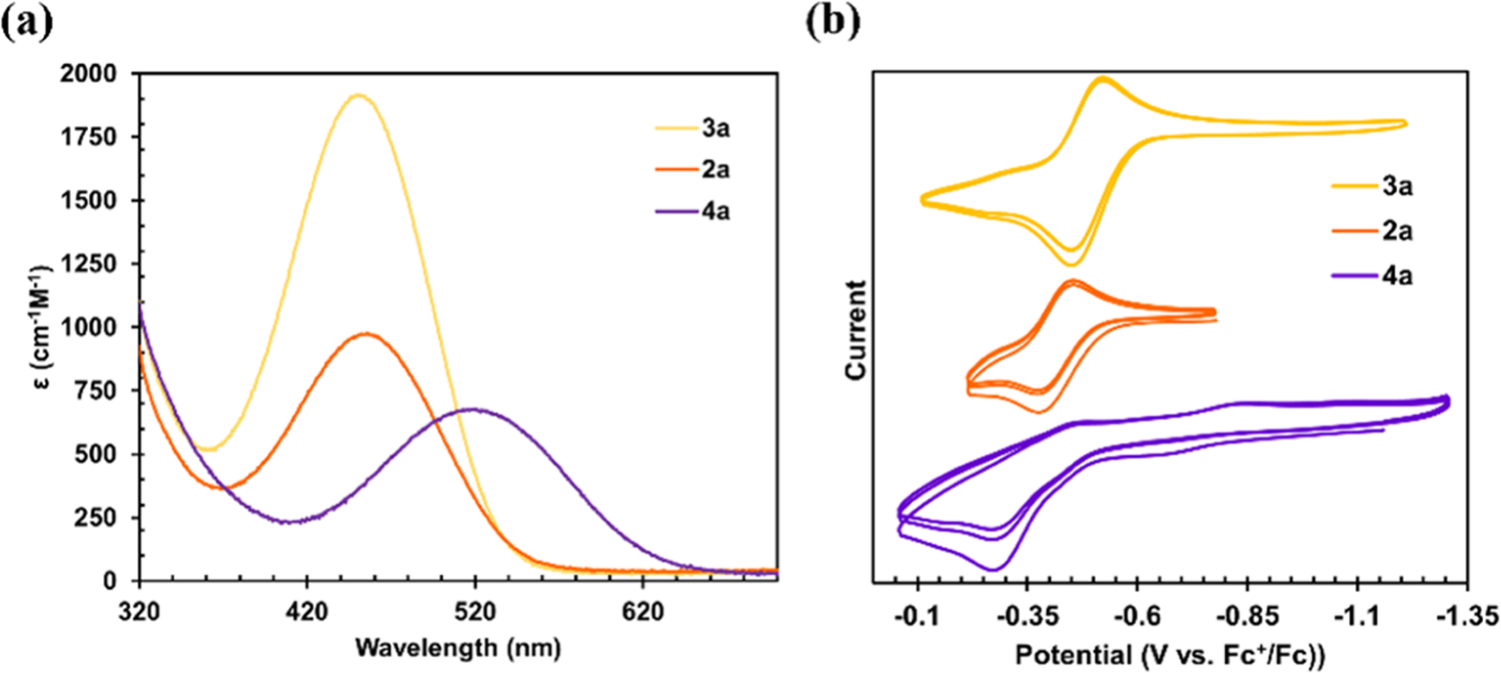
(a) UV–vis spectra for [(Py)_2_Fe(^Ph^Dbf)] (**2a**), [(*p*-*^t^*Bu-Py)_2_Fe(^Ph^Dbf)] (**3a**), and [(*p*-CF_3_–Py)_2_Fe(^Ph^Dbf)] (**4a**). (b) Cyclic voltammograms showing the oxidation wave for [(Py)_2_Fe(^Ph^Dbf)] (**2a**), [(*p*-*^t^*Bu-Py)_2_Fe(^Ph^Dbf)] (**3a**), and [(*p*-CF_3_–Py)_2_Fe(^Ph^Dbf)] (**4a**).

As noted above, unlike **1a** which is a gray-white solid, all pyridine-coordinated iron complexes display intense colors in the range of yellow/orange–pink/purple. UV–vis spectra were collected in benzene and display bands at 450 nm (**2a**), 452 nm (**3a**), 517 nm (**4a**), 515 nm (**5a**), and 491 nm (**6a**) (Figures 4a and S1), with molar absorption coefficients ranging between 500 and 2000 M^–1^ cm^–1^. Interestingly, no significant difference between **2a** and **3a** can be determined, albeit the molar extinction coefficient of **3a** is the highest among the series. To better understand the observed electronic transitions, we carried out time-dependent density functional theory (TD-DFT) calculations on the three monomeric complexes for which we had crystallographic data. Results showed normal deviations between the calculated and experimental electronic transitions typically observed in the literature.^40,43^ In all cases, two major transitions are noted in the visible region: 506.5 and 497.8 nm (**2a**), 493.1 and 485.1 nm (**3a**), 594.5 and 580 nm (**4a**). Given the nearly identical λ_max_ for **2a** and **3a** and the error often noted for calculated absorption spectra, we can only make a comment on the trend that as the pyridine ligand becomes more electron-rich, the observed λ_max_ decreases, which is indeed what the TD-DFT results predict. Nevertheless, examination of the two major transitions identified suggests that these are primarily metal-to-ligand charge transfer (MLCT) in character. Analysis of the natural transition orbitals reveals that the MLCT occurs from the nonbonding *d*_*yz*_ orbital into the lowest pyridine π* orbital for each of the pyridine ligands (Figures 3b, S70, and S71). Assuming no major geometrical changes, the nonbonding *d*_*yz*_ orbital is expected to remain relatively unperturbed upon changes of the pyridine substituent as it does not participate in bonding interactions. The antibonding orbitals of pyridine are, however, impacted by the group introduced at the *para* position, with lower energies being expected for electron-withdrawing substitution and higher energies for more electron-rich pyridine donors. As such, the energy of the MLCT band is directly correlated to the electron-richness of the pyridine ligands, with the lowest absorption energy, and thus the highest absorption wavelength, predicted for the most electron-poor donors. This observation is consistent with the experimental results showing the trend in λ_max_ to be **4a** > **2a** ∼ **3a**, indicating the similarity of **2a** and **3a**, both in experiment and DFT.

**Figure 5:**
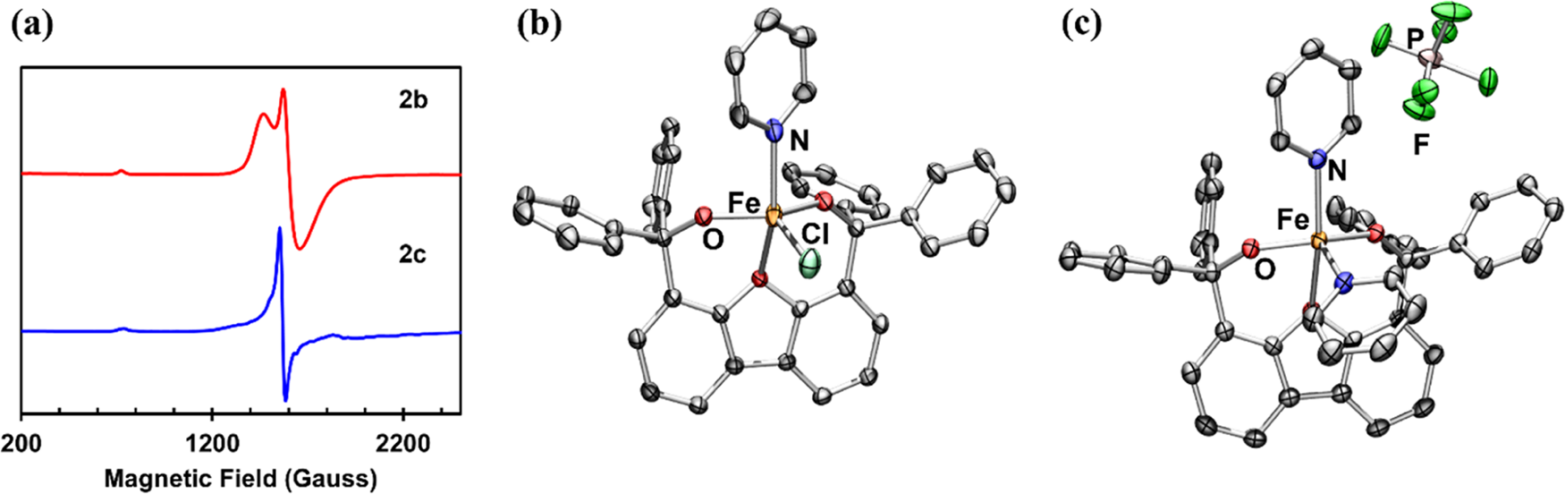
(a) Frozen toluene EPR spectra of [PyFe(^Ph^Dbf)Cl] (**2b**, red) and [Py_2_Fe(^Ph^Dbf)][PF_6_] (**2c**, blue) collected at 80 K. Solid-state molecular structures for (b) [PyFe(^Ph^Dbf)Cl] (**2b**) and (c) [Py_2_Fe(^Ph^Dbf)][PF_6_] (**2c**) with anisotropic displacement ellipsoids at 50% probability level. Color scheme: Fe, orange; N, blue; O, red; F, green; Cl, aquamarine; P, pink. Hydrogens, solvent molecules, and disordered pyridine and chloride groups in **2b** are omitted for clarity.

To further understand the impact of the substituted pyridine ligand on the electronic properties of the complexes, cyclic voltammetry (CV) studies were carried out for each of the isolated species in 1,2-difluorobenzene. Two irreversible oxidation events can be observed for **1a** at *E*_pa_: −0.210 and +1.01 V (Figures S52 and S53), suggesting that a chemical reaction distorts the dimeric motif or that a dimeric structure may not be stable as the iron centers become more electrophilic. The CV spectra of **5a** and **6a** reveal similar features (Figures S62–S67), consistent with their dimeric nature. Indeed, the second irreversible oxidation around +1 V is absent for all monomeric species analyzed. Instead, each of the monomers shows a single oxidation event (Figures 4b and S54–S61), which is reversible for **2a** and **3a** (*E*_1/2_ = −0.412 V (**2a**); −0.487 V (**3a**)), but irreversible for **4a** (*E*_pa_ = −0.275 V) (Figure 4b). As expected, the oxidative reduction potential becomes more positive as the pyridine becomes more electron-poor. Further, no reductive waves could be detected for any of these complexes, an observation which is consistent with our failed attempts to chemically reduce **1a**–**6a** with strong reductants such as potassium graphite or sodium naphthalene. If reactivity occurs, it is likely that the resulting complexes are very unstable. Unfortunately, due to the presence of small impurities in the complexes, additional waves are noted in the CV spectra, albeit of lower intensity, which suggests they are likely not directly associated with the main species. We think that all of the waves noted in the reduction scan are a result of the lower-intensity oxidative waves given that they are not observed if the scan is performed reductively first or does not go past the respective oxidative waves.

### Reactivity of **1a**–**6a**

**Scheme 2:**
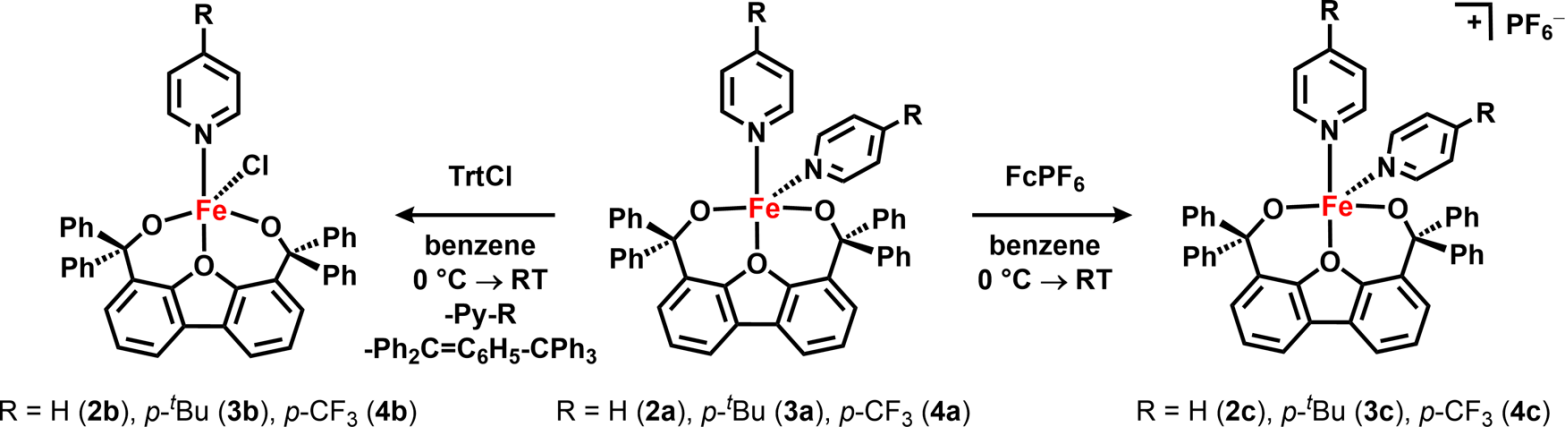
Oxidation of Iron Alkoxide Bis-Pyridine Complexes

Inspired by the CV data and motivated by our interest in pursuing oxidative chemistry with these iron alkoxide complexes, we sought to chemically explore the redox chemistry of **1a**–**6a**. The addition of trityl chloride to any of the monomeric iron complexes results in the formation of new species (**2b**–**4b**, Scheme 2). ^1^H NMR analysis reveals commonalities in the spectra of the products (Figure S32), suggesting that the oxidation proceeds similarly regardless of what pyridine ligand is bound to the iron center. Likewise, outer-sphere oxidation of **2a**–**4a** can also be accomplished in the presence of ferrocenium hexafluorophosphate to afford **2c**–**4c** (Scheme 2). Evaluating these new series of complexes by electron paramagnetic resonance (EPR) spectroscopy reveals similar spectra (Figure 5a, S41–S43, and S46–S48) indicative of high-spin ferric centers with high |*E*/*D*| (0.245–0.255 for **2b**–**4b** and 0.27**–**0.29 for **2c**–**4c**, Table S10).^44^ Diffusing hexanes into a frozen benzene solution of **2b** or a solution of **2c** in trifluorotoluene at −35 °C afforded yellow and orange X-ray quality crystals, respectively, which were probed via X-ray diffraction. In both instances, five-coordinate iron alkoxide complexes were identified, [PyFe(^Ph^Dbf)Cl] (**2b**) and [Py_2_Fe(^Ph^Dbf)][PF_6_] (**2c**), respectively, with one of the pyridine ligands being displaced by a chloride in **2b** (Figure 5b,c). A comparison of the bond metrics of **2b**, **2c**, and **2a** shows shorter Fe–O (**2a**: 2.3380(8) Å; **2b**: 2.2354(14) Å; **2c**: 2.2117(12) Å) and Fe–N (**2a**: 2.1517(10) Å; **2b**: 2.046(10) Å, **2c**: 2.0796(14) Å) bond lengths for the ferric centers, consistent with the tighter metal–ligand binding expected upon oxidation of the iron center. The overall geometry of **2c** is not significantly altered, however, the distortion from square planar is noticeable in **2b**, with the iron center being pulled above the OOON square plane.

Attempts to oxidize **1a**, **5a**, and **6a** to probe the formation of dimeric ferric species were similarly pursued. Addition of two equivalents of trityl chloride to **1a** gives rise to a new species (**1b**) as detected by ^1^H NMR spectroscopy. Interestingly, the proton resonances are similar to those observed for inner-sphere oxidation of the monomeric iron complexes (Figure S33) and the EPR spectrum observed for **1b** (Figure S40) displays signals almost identical to those found for **2b**–**4b**. These findings suggest that chloride anions coordinate to each of the iron centers and the bridging alkoxide now serves as the equivalent L-type donor ligand. Alternatively, formation of a bis-alkoxide diiron complex featuring bridging chlorides cannot be excluded. We do not think dissociation into a monomeric iron species is occurring due to the absence of an additional coordinating ligand to fully stabilize the iron center. Similarly, examination of the ^1^H (Figures S26, S28, and S33) and ^19^F (Figures S27 and S29) NMR spectra of the complexes formed upon the reaction of **5a** and **6a** with two equivalents of trityl chloride (**5b** and **6b**) reveals formation of species analogous to **4b**. Nevertheless, a single ferric EPR signal is detected (Figures S44 and S45), arguing against the presence of two distinct ferric species that could result if dissociation into monomeric iron complexes occurred. The EPR spectrum of **5b** does show the presence of an organic radical as well, which could be due to the Gomberg’s dimer byproduct which is very difficult to wash away due to solubility issues or could be indicative of decomposition with this particular complex. Further, a zero-field ^57^Fe Mössbauer spectrum of an in situ generated **5b** (Figure S13) revealed a single Fe^III^ center (66% δ = 0.46 mm/s, |Δ*E*_Q_| = 1.19 mm/s), along with a 34% of a species indicative of a ferrous center (δ = 1.05 mm/s, |Δ*E*_Q_| = 2.68 mm/s). Unfortunately, the broadness of the signal cannot exclude the presence of multiple ferric centers and we are uncertain at this time if the Fe^II^ center is a result of decomposition or otherwise generated during the reaction.

Reactions of the dimeric complexes with outer-sphere oxidants afford different results. We find that exposure of **1a** to ferrocenium hexafluorophosphate provides an EPR spectrum that features a significant amount of an organic radical along with a small ferric signal (Figure S49). Analysis of the ^1^H NMR spectrum indicates formation of a new species, albeit it is unclear whether the dimer retains its structure. If the oxidation is carried out in a coordinating solvent such as THF, the respective monomeric iron bis-THF complex [(THF)_2_Fe(^Ph^Dbf)][PF_6_] (**7**) is obtained (Figure S80), supporting the hypothesis that additional stabilization is needed as the iron centers become more electron-poor. Addition of one equivalent of Lewis base does not appear to be sufficient to completely stabilize an oxidized dimeric species. However, when **5a** or **6a** is exposed to ferrocenium hexafluorophosphate, the EPR reveals a significant amount of a high-spin Fe^III^ species along with an organic radical (Figures S50 and S51). Interestingly, the ferric signal is almost identical to that of **4c** (Figure S50), suggesting that dissociation into a monomeric species could be favored upon oxidation. That would also explain the formation of the organic radical resulting from the oxidized **1a** that would be formed upon dissociation. These observations are consistent with the irreversible oxidations noted in the CV spectra of **1a**, **5a**, and **6a**. Unfortunately, attempts to crystallographically characterize these species were unsuccessful.

## Conclusions

We have synthesized a series of alkoxide-supported bimetallic and monometallic iron complexes. All complexes display high-spin configurations owing to the weak-field nature of the alkoxide ligand. Results showed that the dimeric form is favored in the presence of weak Lewis bases, whereas addition of electron-rich pyridine ligands encourages conversion to monomeric complexes. Interestingly, we have identified that electron-poor pyridines such as 4-trifluoromethylpyridine and likely 3-trifluoromethylpyridine enable isolation of diiron complexes coordinating a single pyridine ligand, but only the former can support both the monomeric and dimeric complexes. Further, whereas the diiron alkoxide complex is colorless, the respective pyridine-bound complexes are intensely colored due to MLCT transitions which were identified using TD-DFT. The complexes can participate in oxidative reactivity with both inner and outer-sphere oxidants. Diiron complexes, however, do not appear to be stable upon oxidation in the absence of additional coordinating ligands, with either generation of organic radicals or possible dissociation into monomers taking place. Overall, these results illustrate the utility of alkoxide ligands to support well-defined bi- and monometallic iron complexes as well as the tunability of the nuclearity of these systems using auxiliary pyridine ligands. Such fine-tuning in coordination environment could have implications in reactivity control. Investigations regarding the reactivity of these complexes with [NR] and [O] atom donors are currently being explored.

## Experimental Section

### General Considerations

All manipulations of metal complexes were carried out in the absence of water and dioxygen using standard Schlenk techniques, or in a Vigor inert atmosphere dry box under a dinitrogen atmosphere. The bis-alkoxide ligand was synthesized as previously reported.^39^ All glassware was oven-dried for a minimum of 3 h and cooled in an evacuated antechamber prior to use in the dry box. Benzene, diethyl ether, hexane, pentane, toluene, tetrahydrofuran, 1,2-difluorobenzene, and trifluorotoluene were dried over 4 Å molecular sieves (Research Catalysts) prior to use. Benzene-*d*_6_ was purchased from Cambridge Isotope Laboratories and was degassed and stored over 4 Å molecular sieves prior to use. Pyridine, *n*-butyllithium, and benzophenone were purchased from Aldrich. Dibenzofuran, 3-(trifluoromethyl)pyridine, 4-(trifluoromethyl)pyridine, and 4-*tert*-butylpyridine were purchased from Oakwood Chemical. Triphenylmethyl chloride was purchased from Acros Organics. Ferrocenium hexafluorophosphate was synthesized following a previously reported procedure.^45^ Fe_2_Mes_4_ was synthesized as previously reported.^46^ Celite 545 (J. T. Baker) was dried in a Schlenk flask for 24 h under dynamic vacuum while heating to at least 150 °C prior to use in a dry box. Silica gel 32–63 μ (AIC, Framingham, MA) was used as received.

### Characterization and Physical Measurements

^1^H NMR spectra were recorded on Bruker Avance III 600 MHz with a TCI LN2 Prodigy probe, a Bruker Avance II 500 MHz with a BBO LN2 Prodigy probe, or a JEOL ECZL400S 400 MHz with a Royal HFX probe system. ^19^F NMR spectra were recorded on Bruker Avance III 600 MHz with a TCI LN2 Prodigy probe or a JEOL ECZL400S 400 MHz with a Royal HFX probe system. ^1^H and ^13^C NMR chemical shifts are reported relative to SiMe_4_ using the chemical shift of residual solvent peaks as reference. ^19^F NMR chemical shifts are reported relative to an internal standard of trifluorotoluene.^47^ All ^1^H and ^19^F NMR spectra were recorded at room temperature. Elemental analyses were carried out by Midwest Microlab (Indianapolis, IN). Zero-field ^57^Fe Mössbauer spectra were measured with a constant acceleration spectrometer (SEE Co, Minneapolis, MN) at 90 K. Isomer shifts are quoted relative to Fe foil at room temperature. Data was analyzed and simulated with Igor Pro 6 software (WaveMetrics, Portland, OR) using Lorentzian fitting functions. Samples were prepared by suspending 25–50 mg of compound in sufficient paratone oil and immobilizing by rapid freezing in liquid nitrogen. EPR spectra were obtained on a Bruker EMX-Plus CW-EPR spectrometer. Spectra were measured as frozen toluene glasses at a microwave power of 0.6325–2 mW, at 80 K. Spectral simulations incorporating spin state and rhombicity were performed using VisualRhombo.^44^ UV/visible spectra were recorded on an Agilent Cary 60 UV/visible spectrometer using quartz cuvettes and a slow scan rate in benzene. Extinction coefficients were determined from a minimum of five concentrations per sample and were calculated by a linear regression fit of the absorbance vs concentration data. Solution magnetic susceptibilities were determined by the method of Evans^48,49^ (room temperature) using trifluorotoluene as an internal reference with diamagnetic corrections applied as described in the work of Bain et al.^50^

### X-ray Diffraction Techniques

All structures were collected on a Rigaku Oxford Diffraction Synergy-S diffractometer equipped with a HyPix6000HE detector and operating with a Cu Kα (1.54184 Å) or Mo Kα (0.71073 Å) radiation source. Data collection, unit cell refinement, and data processing were carried out with CrysAlisPro,^51^ while structures were solved utilizing SHELXT^52^ and refined using SHELXL^53^ via Olex2.^54^ Olex2, PovRay,^55^ and ORTEP^56^ applications were used to generate structure graphics. Crystals were mounted on a cryoloop or glass fiber pin using Paratone N oil. Structures were collected at 100 K. All nonhydrogen atoms were refined anisotropically. Hydrogen atoms were placed at idealized positions and refined using a riding model. The isotropic displacement parameters of all hydrogen atoms were fixed to 1.2 times the atoms they are linked to (1.5 times for methyl groups). Further details on particular structures are noted in the Supporting Information.

### Computational Methods

Computations were carried out utilizing the ORCA 4.2.1^57^ program package. The B3LYP^58,59^ functional was used with the def2-TZVP (Fe, O, N, Cl) and def2-SV(P) (C, H) basis sets.^60−62^ For single-point and property calculations, the def2-TZVP/J (Fe, O, N, Cl) and def2-SVP/J (C, H) auxiliary basis sets^63^ were employed to utilize the expedient RIJCOSX^64^ approximation. All coordinates were taken from X-ray structures.

### Mössbauer

Mössbauer parameters were obtained from the single-point calculations, following methods described by Neese.^65,66^ Quadrupole splittings (Δ*E*_Q_) were calculated from electric field gradient, eq 1.
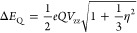
1The nuclear quadruple moment Q(^57^Fe) was taken to be 0.16 barn.^65^ The principal tensor components of the EFG are *V*_*xx*_, *V*_*yy*_, and *V*_*zz*_, from which the asymmetry parameter η = (*V*_*xx*_ – *V*_*yy*_)/*V*_*zz*_ can be defined.

Isomer shifts (δ) were calculated from the electron density at the nucleus ρ_0_, using a linear equation, eq 2,^65^ with constants determined by fitting the calculated densities to experimental isomer shifts for a series of iron alkoxide complexes synthesized in the lab. The basis sets and functionals described above were used, coordinates were obtained from the X-ray structures, and spin states were assigned based on experimental Mössbauer data.
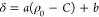
2For this series of compounds, the parameters were determined to be *C* = 11580 au^–3^, *a* = −0.359 au^3^ mm s^–1^, and *b* = 1.295 mm.

### TD-DFT

Geometry optimizations were performed using ORCA 4.2.1 in the gas phase with standard cutoffs using the same method and basis sets as described above. The optimized structures were then probed to be minima by frequency calculations (zero imaginary frequencies) using Gaussian09^67^ at the same level of theory and further optimized as necessary until no imaginary frequencies were found. Optical excitations were then obtained using TD-DFT calculations with the same method and basis set and implicit solvation (CPCM, benzene) using Gaussian09.^67^

### Safety Statement

*Caution*! Fe_2_Mes_4_ is air-sensitive and pyrophoric. Upon synthesis, all glassware and residual material is quenched with acetonitrile inside the glovebox before removal from the glovebox. This is sufficient to ensure the material will not ignite once exposed to air–caution should be taken, nevertheless.

*Caution*! Extreme care should be taken both in the handling of the cryogen liquid nitrogen and its use in the Schlenk line trap or glovebox vacuum trap to avoid the condensation of oxygen from air.

### Metal Complexes Syntheses

#### [Fe_2_(^Ph^Dbf)_2_] (**1a**)

Fe_2_Mes_4_ (110.5 mg, 0.188 mmol) was frozen in minimal benzene. ^**Ph**^**Dbf** (2 equiv; 200 mg, 0.376 mmol) was weighed and suspended in benzene before addition to the frozen Fe_2_Mes_4_ solution. The reaction mixture was stirred at room temperature for 2 h (blue). The eluted solution was lyophilized, the resulting solid was washed over a Celite plug with hexanes to remove unreacted ligand and impurities (blue solution), and the remaining solid was eluted with benzene. Upon lyophilization, a gray/white powder was obtained (**1a**) in 70% yield (140 mg). Crystals suitable for X-ray diffraction were grown from a concentrated ether solution at room temperature. ^**1**^**H NMR** (600 MHz, C_6_D_6_): δ (ppm) 42.48 (br. s), 25.69 (br. s), 19.94 (br. s), 17.37 (br. s), 10.03 (br. s), 6.54 (br. d). Anal. calcd for C_76_H_52_Fe_2_O_6_: C 77.82; H 4.47, N 0; Found C 73.29, H 4.78, N 0. Zero-field ^57^Fe Mössbauer (90 K) δ = 0.92 mm/s, |Δ*E*_Q_| = 1.12 mm/s.

#### [Py_2_Fe(^Ph^Dbf)] (**2a**)

Fe_2_Mes_4_ (55.2 mg, 0.093 mmol) was frozen in minimal benzene. Pyridine (4 equiv; 26.7 mg, 0.372 mmol) was diluted in 1 mL of benzene and was added to the frozen Fe_2_Mes_4_ solution, and the solution was stirred at room temperature for 10 min (light orange/red solution) and refrozen. The ^**Ph**^**Dbf** ligand (2 equiv; 99.1 mg, 0.186 mmol) was suspended in benzene (partially soluble). The ligand was added to the frozen solution and stirred at room temperature for 1 h resulting in an orange solution. The reaction mixture was lyophilized and washed over a Celite pipet with hexanes to remove organic impurities and unreacted ligand. The complex was then eluted with THF and triturated with hexanes to remove any excess pyridine. The complex was dissolved in benzene and lyophilized to obtain **2a** as an orange powder in 88% yield (123 mg). Crystals suitable for X-ray diffraction were grown from a benzene solution at room temperature. ^**1**^**H NMR** (600 MHz, C_6_D_6_): δ (ppm) 37.41 (br. s), 33.16 (br. s), 22.24 (br. s), 17.03(br. s), 9.72 (br. s), 8.36 (d). Anal. calcd C_48_H_36_FeN_2_O_3_: C 77.42, H 4.87, N 3.76; Found C 73.43, H 5.01, N 3.07. Zero-field ^57^Fe Mössbauer (90 K) δ = 1.02 mm/s, |Δ*E*_Q_| = 1.43 mm/s. μ_eff_ (298 K, Evans) 4.67 μ_B_.

#### [(R–Py)_2_Fe(^Ph^Dbf)] (**3a**), (**4a**)

##### General Procedure A

Fe_2_Mes_4_ (0.093 mmol) was frozen in minimal benzene. The substituted pyridine (4 equiv; 0.372 mmol) was diluted in 1 mL of benzene before addition to the frozen Fe_2_Mes_4_ solution. The reaction mixture was stirred at room temperature for 10 min, resulting in either a bright orange or dark blue solution which was refrozen. The ^**Ph**^**Dbf** ligand (2 equiv; 0.186 mmol) suspended in benzene (partially soluble) was added to the frozen solution and stirred at room temperature for 1 h. The reaction mixture was lyophilized, and the resulting solid was eluted through a Celite plug in ether to remove unreacted ligand and organic and paramagnetic impurities (gray). The complex was dried and eluted through a Celite pipet in THF to remove other suspected paramagnetic impurities (gray). The complex was triturated with hexanes to remove excess THF or ether solvent before lyophilization to afford a colored powder.

**[(*****p*****-**^***t***^**Bu–Py)**_**2**_**Fe(**^**Ph**^**Dbf)] (3a)** was synthesized according to general procedure A to obtain a yellow/orange powder in 88% yield (142 mg). ^**1**^**H NMR** (600 MHz, C_6_D_6_): δ (ppm) 35.44 (br. s), 22.49 (br. s), 17.20 (br. s), 9.17 (d), 8.26 (d), −2.02 (br. s). Anal. calcd C_56_H_52_FeN_2_O_3_: C 78.50, H 6.12, N 3.27; Found C 75.31, H 6.07, N 3.14. Zero-field ^57^Fe Mössbauer (90 K) δ = 1.01 mm/s, |Δ*E*_Q_| = 1.5 mm/s. μ_eff_ (298 K, Evans) 4.88 μ_B_. Crystals suitable for X-ray diffraction were grown from hexanes diffusing into a THF solution of **3a** at −35 °C.

**[(*****p*****-CF**_**3**_**–Py)**_**2**_**Fe(**^**Ph**^**Dbf)] (4a)** was synthesized according to general procedure A to obtain a purple powder in 75% yield (124.3 mg). ^**1**^**H NMR** (600 MHz, C_6_D_6_): δ (ppm) 35.90 (br. s), 23.31 (br. s), 17.27 (br. s), 12.75 (br. s), 9.46 (br. s), 8.55 (br. s). ^**19**^**F NMR** (564 MHz, C_6_D_6_): δ (ppm) −140.47 (br. s). Anal. calcd C_50_H_34_F_6_FeN_2_O_3_: C 68.19, H 3.89, N 3.18; Found C 68.47, H 4.37, N 2.54. Zero-field ^57^Fe Mössbauer (90 K) δ = 0.99 mm/s, |Δ*E*_Q_| = 1.24 mm/s. μ_eff_ (298 K, Evans) 5.03 μ_B_. Crystals suitable for X-ray diffraction were grown from hexanes diffusing into a frozen benzene solution of **4a** at −35 °C.

#### [(*p*-CF_3_–Py)Fe_2_(^Ph^Dbf)_2_] (**5a**) and [(*m*-CF_3_–Py)Fe_2_(^Ph^Dbf)_2_] (**6a**)

##### Procedure a

Complex **1a** (50 mg; 0.586 mmol) was diluted in 2 mL of benzene. An aliquot of a *p*-CF_3_Py (86 mg; 0.586 mmol) or *m*-CF_3_Py (86 mg; 0.586 mmol) stock solution in benzene was added to **1a**. An instantaneous color change to purple was observed. The solution was lyophilized to obtain **5a** as a purple powder or **6a** as a pink powder in quantitative yield.

##### Procedure b

Fe_2_Mes_4_ (0.093 mmol) was frozen in minimal benzene. The substituted pyridine (0.5 equiv; 0.047 mmol) was diluted in 1 mL of benzene before addition to the frozen Fe_2_Mes_4_ solution. The reaction mixture was stirred at room temperature for 10 min and refrozen. The ^**Ph**^**Dbf** ligand (2 equiv; 0.186 mmol) suspended in benzene (partially soluble) was added to the frozen solution and stirred at room temperature for 1 h. The reaction mixture was lyophilized, and the resulting solid was eluted through a Celite plug in ether to remove unreacted ligand and organic and paramagnetic impurities (gray). The complex was dried and eluted through a Celite pipet in THF to remove other suspected paramagnetic impurities (gray). The complex was triturated with hexanes to remove excess THF or ether solvent before lyophilization to afford a colored powder ((**5a**)—light purple, (**6a**)—pink) in 72% yield (89 mg) and 59% yield (77 mg), respectively. Crystals suitable for X-ray diffraction were grown from hexanes diffusing into a benzene solution of (**5a**) at −35 °C.

*Note: These complexes will degrade overnight at room temperature in solution. These complexes degrade in the solid state in the freezer over extended periods of time (∼4 weeks) and more rapidly at room temperature.

##### [(*p*-CF_3_–Py)Fe_2_(^Ph^Dbf)_2_] (**5a**)

^**1**^**H NMR** (600 MHz, C_6_D_6_): δ (ppm) 41.64 (br. s), 35.97 (br. s), 31.60 (br. s), 23.09 (br. s), 18.60 (br. s) 17.10 (br. s), 14.50 (br. s), 12.49 (br. s), −3.38 (br. s), −9.01 (br. s), −14.79 (br. s), −31.08 (br. s). ^**19**^**F NMR** (564 MHz, C_6_D_6_): δ (ppm) −119.23 (br. s). Anal. calcd C_82_H_56_F_3_Fe_2_NO_6_: C 74.61, H 4.28, N 1.06; Found C 73.52, H 4.93, N 1.11. μ_eff_ (298 K, Evans) 6.76 μ_B_.

##### [(*m*-CF_3_–Py)Fe_2_(^Ph^Dbf)_2_] (**6a**)

^**1**^**H NMR** (600 MHz, C_6_D_6_): δ (ppm) 41.40 (br. s), 35.74 (br. s), 31.42 (br. s), 22.92 (br. s), 18.64 (br. s), 17.11 (br. s), 14.50 (br. s), 12.76 (br. s), 9.43 (br. s), 8.51 (br. s), −0.38 (br. s), −3.66 (br. s), −8.87 (br. s), −14.67 (br. s), −30.41 (br. s). ^**19**^**F NMR** (564 MHz, C_6_D_6_): δ (ppm) −29.76 (br. s). Anal. calcd C_82_H_56_F_3_Fe_2_NO_6_: C 74.61, H 4.28, N 1.06; Found C 72.12, H 4.92, N 1.12. Zero-field ^57^Fe Mössbauer (90 K) 50% δ = 1.02 mm/s, |Δ*E*_Q_| = 1.35 mm/s and 50% δ = 1.13 mm/s, |Δ*E*_Q_| = 1.93 mm/s.

### Reactions with Trityl Chloride

#### General Procedure B

Trityl chloride (2 equiv) was frozen in benzene. A thawing benzene solution of the corresponding complex (1 equiv; 20 mg) was added and allowed to stir at room temperature for 10 min. Gradual color changes were observed. The reaction mixture was lyophilized and washed over a Celite plug with hexanes to remove Gomberg’s dimer. The resulting solid was recollected in benzene and lyophilized to obtain a powder. Note that the high solubility of the resulting complexes leads to unavoidable loss of product in the hexanes wash.

**[Fe**_**2**_**(**^**Ph**^**Dbf)**_**2**_**(Cl)**_**2**_**]** (**1b**) was synthesized according to general procedure B to afford a pale-yellow powder in 67% yield (14 mg). ^**1**^**H NMR** (600 MHz, C_6_D_6_): δ (ppm), 25.24 (br. s), 19.91 (br. s), 17.08 (br. s), 13.74 (br. s), 5.21 (br. d). Anal. calcd C_76_H_52_Cl_2_Fe_2_O_6_: C 73.39, H 4.21, N 0; Found C 62.31, H 4.15, N 0. EPR (toluene, 80 K): *g*_eff_ = 9.28, 4.46, 4.22.

**[(*****p*****-CF**_**3**_**-Py)Fe**_**2**_**(**^**Ph**^**Dbf)**_**2**_**(Cl)**_**2**_**]** (**5b**) was synthesized according to general procedure B to afford a brown powder in 15% yield (3 mg). ^**1**^**H NMR** (600 MHz, C_6_D_6_): δ (ppm) 31.29, 25.98, 22.87, 21.71, 19.12, 16.49, 14.8, 3.64. ^**19**^**F NMR** (564 MHz, C_6_D_6_): δ (ppm) −52.70 (br. s). Anal. calcd C_82_H_56_Cl_2_F_3_Fe_2_NO_6_ (assuming a dimeric structure): C 70.81, H 4.06, N 1.01; Found C 64.44, H 4.35, N 0.49. Zero-field ^57^Fe Mössbauer (90 K) 66% δ = 0.46 mm/s, |Δ*E*_Q_| = 1.19 mm/s; 34% δ = 1.05 mm/s, |Δ*E*_Q_| = 2.68 mm/s. EPR (toluene, 80 K): *g*_eff_ = 9.74, 4.44, 4.24.

**[(*****m*****-CF**_**3**_**-Py)Fe**_**2**_**(**^**Ph**^**Dbf)**_**2**_**(Cl)**_**2**_**]** (**6b**) was synthesized according to general procedure B on a 50 mg complex scale to afford a brown powder in 27% yield (16 mg). ^**1**^**H NMR** (600 MHz, C_6_D_6_): δ (ppm) 22.86 (br. s), 19.24 (br. s), 18.96 (br. s), 16.47 (br. s), 15.55 (br. s), 14.46 (br. s), 11.06 (br. s), −8.42 (br. s), −8.78 (br. s), −14.43 (br. s), −25.94 (br. s). ^**19**^**F NMR** (564 MHz, C_6_D_6_): δ (ppm) −41.45 (br. s). Anal. calcd C_82_H_56_Cl_2_F_3_Fe_2_NO_6_ (assuming a dimeric structure): C 70.81, H 4.06, N 1.01; Found C 67.60, H 4.54, N 0.92. EPR (toluene, 80 K): *g*_eff_ = 9.5, 4.52, 4.25.

#### General Procedure C

Trityl chloride (1 equiv) was frozen in benzene. A thawing benzene solution of the corresponding complex (1 equiv; 20 mg) was added and allowed to stir at room temperature for 10 min. Gradual color changes were observed. The reaction mixture was lyophilized and washed over a Celite plug with hexanes to remove Gomberg’s dimer. The resulting solid was recollected in benzene and lyophilized to obtain a powder.

**[PyFe(**^**Ph**^**Dbf)Cl]** (**2b**) was synthesized according to general procedure C to afford a yellow powder in 66% yield (30 mg). ^**1**^**H NMR** (600 MHz, C_6_D_6_): δ (ppm) 21.33 (br. s), 16.32 (br. s), 14.67 (br. s), 11.86 (br. s). Anal. calcd C_43_H_31_ClFeNO_3_: C 73.67, H 4.46, N 2.00; Found C 64.59, H 4.45, N 1.67. Zero-field ^57^Fe Mössbauer (90 K) 72% δ = 0.36 mm/s, |Δ*E*_Q_| = 1.1 mm/s; 28% δ = 0.56 mm/s, |Δ*E*_Q_| = 2.51 mm/s. EPR (toluene, 80 K): *g*_eff_ = 9.35, 4.58, 4.18. Crystals suitable for X-ray diffraction were grown from hexanes diffusing into a frozen benzene solution of **2b** at −35 °C.

*Note: we think the species with an isomer shift of 0.36 mm/s corresponds to **2b**, whereas the species with an isomer shift of 0.56 is either an impurity or decomposition of the sample. We have noticed that the sample can decompose easily either during synthesis or during workup—the most noticeable way being the appearance of additional ferric signals in the EPR.

**[(*****p*****-**^***t***^**Bu-Py)Fe(**^**Ph**^**Dbf)Cl]** (**3b**) was synthesized according to general procedure C to afford a yellow powder in 63% yield. ^**1**^**H NMR** (600 MHz, C_6_D_6_): δ (ppm) 21.09 (br. s), 16.41 (br. s), 14.74 (br. s), 12.014 (br. s), 2.31 (br. s). Anal. calcd C_47_H_39_ClFeNO_3_: C 74.56, H 5.19, N 1.85; Found C 72.16, H 5.32, N 1.99. EPR (toluene, 80 K): *g*_eff_ = 9.24, 4.59, 4.16.

**[(*****p*****-CF**_**3**_**-Py)Fe(**^**Ph**^**Dbf)Cl]** (**4b**) was synthesized according to general procedure C to afford a yellow powder in 14.2% yield (6 mg). ^**1**^**H NMR** (600 MHz, C_6_D_6_): δ (ppm) 22.54 (br. s), 18.93 (br. s), 16.29 (br. s), 14.36 (br. s), 11.61 (br. s). ^**19**^**F NMR** (564 MHz, C_6_D_6_): δ (ppm) −53.67 (br. s). Anal. calcd C_44_H_30_ClF_3_FeNO_3_: C 68.72, H 3.93, N 1.82; Found C 67.28, H 4.52, N 1.91. EPR (toluene, 80 K): *g*_eff_ = 9.09, 4.56, 4.24.

*Note that the high solubility of **4b** leads to unavoidable loss of product in the hexanes wash.

### Reactions with Ferrocenium Hexafluorophosphate

#### General Procedure D

FcPF_6_ (2 equiv) was frozen in benzene. A thawing benzene solution of the ferrous complex (1 equiv; 20.0 mg) was added to the frozen solution and allowed to stir at room temperature for 10 min. Gradual color changes were observed. The reaction mixture was lyophilized, and the residue was washed with hexanes over a Celite pipet to remove ferrocene. The product was eluted in benzene and lyophilized to obtain a powder.

*Note that if the reactions are allowed to run for longer than 10 min, the complexes start rapidly decomposing. To avoid this, the complexes are frozen in the coldwell immediately after the color change is noted (∼10 min). Decomposition can be identified by the appearance of a doublet of doublets and a doublet of quintets in the ^19^F NMR, as opposed to the silent ^19^F NMR signal for the complex. Severe decomposition is noted by the appearance of many small paramagnetic peaks in the ^1^H NMR.

General procedure D was applied to oxidation of **1a**, **5a**, and **6a**. In all cases, EPR data revealed formation of an organic radical, with a minimal component of a metal-based species, suggesting potential decomposition. Unfortunately, we were unable to crystallographically characterize any of the species and further spectroscopic characterization was not pursued. EPR and ^1^H NMR data are provided in the Supporting Information (Figures S37–S39 and S49–S51).

#### [[(THF)_2_Fe(^Ph^Dbf)]PF_6_] (**7**)

FcPF_6_ (2 equiv) was frozen in tetrahydrofuran. A thawing tetrahydrofuran solution of **1a** (1 equiv) was added to the frozen solution and allowed to stir at room temperature for 10 min. Gradual color changes were observed, and a precipitate was noted in the solution. The reaction mixture was filtered through Celite. The precipitate was washed with hexanes, eluted with 1,2-difluorobenzene, and concentrated to a gray powder. Crystals suitable for X-ray diffraction were grown from diffusing pentane into a solution of the complex in 1,2-difluorobenzene at −35 °C. Although the structure model was of poor quality, it clearly identified the complex as [(THF)_2_Fe(^Ph^Dbf)][PF_6_] (Figure S80). However, due to the insolubility of the complex in typical solvents, additional characterization was not pursued.

#### General Procedure E

FcPF_6_ (1 equiv) was frozen in benzene. A thawing benzene solution of the complex (1 equiv; 20 mg) was added to the frozen solution and allowed to stir at room temperature for 10 min. Gradual color changes were observed. The reaction mixture was lyophilized and the residue was washed over a Celite pipet to remove ferrocene. The product was eluted in benzene and lyophilized to obtain a powder.

*Note that if the reactions are allowed to run for longer than 10 min, the complexes start rapidly decomposing. To avoid this, the complexes are frozen in the coldwell immediately after the color change is noted (∼10 min). Decomposition can be identified by the appearance of a doublet of doublets and a doublet of quintets in the ^19^F NMR, as opposed to the silent ^19^F NMR signal for the complex. Severe decomposition is noted by the appearance of many small paramagnetic peaks in the ^1^H NMR.

**[[Py**_**2**_**Fe(**^**Ph**^**Dbf)]PF**_**6**_**]** (**2c**) was synthesized according to general procedure E on a 50 mg complex scale and was obtained as a yellow powder in 39% yield (24 mg). ^**1**^**H NMR** (600 MHz, C_6_D_6_): no distinguishable peaks. Anal. calcd C_48_H_36_F_6_FeN_2_O_3_P: C 64.80, H 4.08, N 3.15; Found C 60.61, H 4.22, N 2.84. EPR (toluene, 80 K): *g*_eff_ = 9.2, 5.0, 4.27. Crystals suitable for X-ray diffraction were grown from diffusing hexanes into a trifluorotoluene solution of **2c**.

**[[(*****p*****-**^***t***^**Bu-Py)**_**2**_**Fe(**^**Ph**^**Dbf)]PF**_**6**_**]** (**3c**) was synthesized according to general procedure E and was obtained as a yellow powder in 35% yield (8 mg). ^**1**^**H NMR** (600 MHz, C_6_D_6_): no distinguishable peaks. Anal. Calc. C_56_H_52_F_6_FeN_2_O_3_P: C 67.14, H 5.23, N 2.80; Found C 67.14, H 5.45, N 2.81. EPR (toluene, 80 K): *g*_eff_ = 9.13, 4.25.

**[[(*****p*****-CF**_**3**_**-Py)**_**2**_**Fe(**^**Ph**^**Dbf)]PF**_**6**_**]** (**4c**) was synthesized according to general procedure E on a 50 mg complex scale and was obtained as a yellow powder in 59% yield (34 mg). ^**1**^**H NMR** (600 MHz, C_6_D_6_): no distinguishable peaks. ^**19**^**F NMR** (564 MHz, C_6_D_6_): no signal. Anal. calcd C_50_H_34_F_12_FeN_2_O_3_P: C 58.55, H 3.34, N 2.73; Found C 62.77, H 4.11, N 2.11. EPR (toluene, 80 K): *g*_eff_ = 9.23, 4.28.

### Reactions with KC_8_

KC_8_ (20 mg, 0.148 mmol) was frozen in benzene. A thawing benzene solution of the complex (1 equiv; 0.148 mmol) was added to the frozen solution and allowed to stir at room temperature for 10 min to 1 h. Gradual color changes were observed. The reaction mixture was eluted through a Celite pipet to remove graphite. Complexes were lyophilized to obtain a powder. No clean ^1^H NMR spectra could be obtained, so the reactions were not pursued further.

### Reactions Testing Py Ligand Lability

The iron complex (10 mg) was dissolved in deuterated benzene and transferred to an NMR tube. Substituted pyridine (pyridine, 4*-tert*-butylpyridine, or 4-trifluormethylpyridine) was then added in slight excess (2.1 equiv), and an NMR spectrum was immediately obtained. Instantaneous color change upon addition of the new pyridine was noted. The reaction mixture was then dried under vacuum and triturated with hexanes three times before a final NMR spectrum was obtained.

### Reaction with Lutidine

The iron complex (**1a**; 10 mg) was dissolved in minimal deuterated benzene and transferred to an NMR tube. Lutidine (4.1 equiv) was added to the NMR tube with minimal deuterated benzene. An NMR spectrum was obtained immediately and after 10 min. No obvious color change was observed upon addition of lutidine or after significant time.

Note: Elemental analysis results do not match well with the expected values, likely a result of decomposition as the complexes have varying stability at room temperature in solution and in solid state. The complexes that were observed to be the most unstable were the complexes bearing *p-*CF_3_–Py and *m-*CF_3_–Py, as well as all Fe(III) complexes oxidized with FcPF_6_. In the glovebox and in sealed NMR tubes outside the glovebox, these samples degrade overnight in solution and show evidence of decomposition in the ^19^F NMR if left at room temperature overnight in the glovebox. EA was repeated in duplicate for those complexes that had values far-off from expected in order to support the claim that decomposition is likely contributing to these complexes deviating from expected values. As noted in the Mössbauer spectra, the complexes do show a small amount of impurity that could be a result of decomposition upon shipping the sample, which would agree with the EA not giving expected values. Furthermore, the Fe(III) complexes that were oxidized in the presence of FcPF_6_ were notably unstable (as evident by ^1^H NMR and EPR) and did not survive shipping for Mössbauer studies as well, suggesting that these complexes are very susceptible to degradation.

## Supporting Information Available

UV–vis spectra for **2a**–**6a**; ^1^H NMR spectra for all complexes synthesized; CV spectra for **1a**–**6a**; EPR spectra for **1b**–**6b** and **2c**–**4c**; ^57^Fe Mössbauer spectra for **1a**–**4a**, **6a**, **2b**, and **5b**; X-ray crystallography tables and figures for **1a**–**4a**, **5a**, **2b**, **2c**, and **7**; frontier molecular orbitals for **2a**; TD-DFT electronic transitions for **2a**, **3a**, and **4a**; and optimized *XYZ* coordinates for **2a**–**4a** (PDF)
