# Supplementary material for: Synthesis, Characterization, and the Effect of Lewis Bases on the Nuclearity of Iron Alkoxide Complexes
Source: Inorg Chem. 2024 Apr 12;63(17):7692–704. doi: 10.1021/acs.inorgchem.3c04538 (PMC11061831; doi:10.1021/acs.inorgchem.3c04538)
Supplement: Supplementary file 1 — ic3c04538_si_001.pdf [file ic3c04538_si_001.pdf]

Synthesis, Characterization, and the Effect of Lewis Bases on the Nuclearity of Iron Alkoxide Complexes

Reilly K. Gwinn,[1] Matthew Williams,[1] Trevor P. Latendresse,[2] Carla Slebodnick,[1] Diego Troya,[1] Tasnema Tarannum[1] and Diana A. Thornton*[1]

1. Department of Chemistry, Virginia Polytechnic Institute and State University, Blacksburg, VA 24061

2. Department of Chemistry and Chemical Biology, Harvard University, Cambridge, MA 02138

Email: diovan@vt.edu

Page

UV-Vis Spectra and Calibration Curves 6

Figure S-1. Stacked UV-Vis spectra 6

Figure S-2. Calibration curve for [$Py_2Fe(^{Ph}Dbf)$] (2a) 7

Figure S-3. Calibration curve for [$(p$-^tBu-$Py)_2Fe(^{Ph}Dbf)$] (3a) 8

Figure S-4. Calibration curve for [$(p$-CF_3-$Py)_2Fe(^{Ph}Dbf)$] (4a) 9

Figure S-5. Calibration curve for [$(p$-CF_3-$Py)Fe_2(^{Ph}Dbf)_2$] (5a) 10

Figure S-6. Calibration curve for [$(m$-CF_3-$Py)Fe_2(^{Ph}Dbf)_2$] (6a) 11

Zero-field ^{57}Fe Mössbauer Spectra 12

Figure S-7. Zero-field ^{57}Fe Mössbauer of [$Fe_2(^{Ph}Dbf)_2$] (1a) 12

Figure S-8. Zero-field ^{57}Fe Mössbauer of [$Py_2Fe(^{Ph}Dbf)$] (2a) 13

Figure S-9. Zero-field ^{57}Fe Mössbauer of [$(^tBuPy)_2Fe(^{Ph}Dbf)$] (3a) 14

Figure S-10. Zero-field ^{57}Fe Mössbauer of [$(p$-$CF_3Py)_2Fe(^{Ph}Dbf)$] (4a) 15

Figure S-11. Zero-field ^{57}Fe Mössbauer of [$(m$-$CF_3Py)Fe_2(^{Ph}Dbf)_2$] (6a) 16

Figure S-12. Zero-field ^{57}Fe Mössbauer of [$Py_2Fe(^{Ph}Dbf)Cl$] (2b) 17

Figure S-13. Zero-field ^{57}Fe Mössbauer of the reaction of [$(p$-CF_3-$Py)Fe_2(^{Ph}Dbf)_2$] (5a) with trityl chloride 18

Nuclear Magnetic Resonance Spectra 19

Figure S-14. ^1H NMR spectra for paramagnetic [$Fe_2(^{Ph}Dbf)_2$] (1a) 19

Figure S-15. ^{1}H NMR spectra for paramagnetic [$Py_2Fe(^{Ph}Dbf)$] (2a) 20
Figure S-16. ^{1}H NMR spectra for paramagnetic [(p-^{t}Bu-Py)$_2$Fe(^{Ph}Dbf)] (3a) 21
Figure S-17. ^{1}H NMR spectra for paramagnetic [(p-CF_3-Py)$_2$Fe(^{Ph}Dbf)] (4a) 22
Figure S-18. ^{19}F NMR spectra for paramagnetic [(p-CF_3-Py)$_2$Fe(^{Ph}Dbf)] (4a) 23
Figure S-19. ^{1}H NMR spectra for paramagnetic [(p-CF_3-Py)Fe_2(^{Ph}Dbf)$_2$] (5a) 24
Figure S-20. ^{19}F NMR spectra for paramagnetic [(p-CF_3-Py)Fe_2(^{Ph}Dbf)$_2$] (5a) 25
Figure S-21. ^{1}H NMR spectra for paramagnetic [(m-CF_3-Py)Fe_2(^{Ph}Dbf)$_2$] (6a) 26
Figure S-22. ^{19}F NMR spectra for paramagnetic [(m-CF_3-Py)Fe_2(^{Ph}Dbf)$_2$] (6a) 27
Figure S-23. ^{1}H NMR spectra for paramagnetic [Fe_2(^{Ph}Dbf)$_2$$Cl_2$] (1b) 28
Figure S-24. ^{1}H NMR spectra for paramagnetic [PyFe(^{Ph}Dbf)Cl] (2b) 29
Figure S-25. ^{1}H NMR spectra for paramagnetic [(p-^{t}Bu-Py)Fe(^{Ph}Dbf)Cl] (3b) 30
Figure S-26. ^{1}H NMR spectra for paramagnetic [(p-CF_3-Py)Fe(^{Ph}Dbf)Cl] (4b) 31
Figure S-27. ^{19}F NMR spectra for paramagnetic [(p-CF_3-Py)Fe(^{Ph}Dbf)Cl] (4b) 32
Figure S-28. ^{1}H NMR spectra for paramagnetic [(p-CF_3-Py)Fe_2(^{Ph}Dbf)$_2$(Cl)$_2$] (5b) 33
Figure S-29. ^{19}F NMR spectra for paramagnetic [(p-CF_3-Py)Fe_2(^{Ph}Dbf)$_2$Cl] (5b) 34
Figure S-30. ^{1}H NMR spectra for paramagnetic [m-CF_3-Py)Fe_2(^{Ph}Dbf)$_2$(Cl)$_2$] (6b) 35
Figure S-31. ^{19}F NMR spectra for paramagnetic [(m-CF_3-Py)Fe_2(^{Ph}Dbf)$_2$(Cl)$_2$] (6b) 36
Figure S-32. Stacked ^{1}H NMR spectra for paramagnetic [(Py)Fe(^{Ph}Dbf)Cl] (2b), [(p-^{t}Bu-Py)Fe(^{Ph}Dbf)Cl] (3b), and [(p-CF3$_3$-Py)Fe(^{Ph}Dbf)Cl] (4b) 37
Figure S-33. Stacked ^{1}H NMR spectra for paramagnetic [(Py)Fe(^{Ph}Dbf)Cl] (2b) and [Fe_2(^{Ph}Dbf)$_2$$Cl_2$] (1b) 38
Figure S-34. ^{1}H NMR spectra for paramagnetic [Py_2Fe(^{Ph}Dbf)][PF_6] (2c) 39
Figure S-35. ^{1}H NMR spectra for paramagnetic [(p-^{t}Bu-Py)$_2$Fe(^{Ph}Dbf)][PF_6] (3c) 40
Figure S-36. ^{1}H NMR spectra for paramagnetic [(p-CF_3-Py)$_2$Fe(^{Ph}Dbf)][PF_6] (4c) 41
Figure S-37. ^{1}H NMR spectra for the reaction of [Fe_2(^{Ph}Dbf)$_2$] (1a) with FcPF_6 42
Figure S-38. ^{1}H NMR spectra for the reaction of [(p-CF_3-Py)Fe_2(^{Ph}Dbf)$_2$] (5a) with FcPF_6 43
Figure S-39. ^{1}H NMR spectra for the reaction of [(m-CF_3-Py)Fe_2(^{Ph}Dbf)$_2$] (6a) with FcPF_6 44
Electron Paramagnetic Resonance (EPR) 45
Figure S-40. Frozen toluene EPR spectrum of [Fe_2(^{Ph}Dbf)$_2$(Cl)$_2$] (1b) at 80 K 45
Figure S-41. Frozen toluene EPR spectrum of [PyFe(^{Ph}Dbf)Cl] (2b) at 80 K 46
Figure S-42. Frozen toluene EPR spectrum of [(p-^{t}Bu-Py)Fe(^{Ph}Dbf)Cl] (3b) at 80 K 47

Figure S-43. Frozen toluene EPR spectrum of [(*p*-CF_3-Py)Fe(^{Ph}Dbf)Cl] (4b) at 80 K 48

Figure S-44. Frozen toluene EPR spectrum of [(*p*-CF_3-Py)Fe_2^{Ph}Dbf)$_2$(Cl)$_2$] (5b) at 80 K 49

Figure S-45. Frozen toluene EPR spectrum of [(*m*-CF_3-Py)Fe_2(^{Ph}Dbf)$_2$(Cl)$_2$] (6b) at 80 K 50

Figure S-46. Frozen toluene EPR spectrum of [Py_2Fe(^{Ph}Dbf)][PF_6] (2c) at 80 K 51

Figure S-47. Frozen toluene EPR spectrum of [(*p*-^{t}Bu-Py)$_2$Fe(^{Ph}Dbf)][PF_6] (3c) at 80 K 52

Figure S-48. Frozen toluene EPR spectrum of [(*p*-CF_3-Py)$_2$Fe(^{Ph}Dbf)][PF_6] (4c) at 80 K 53

Figure S-49. Frozen toluene EPR spectrum of the reaction of [Fe_2(^{Ph}Dbf)$_2$] (1a) with 2 equiv. FcPF_6 at 80 K. 54

Figure S-50. Frozen toluene EPR spectrum of the reaction of [(*p*-CF_3-Py)Fe_2(^{Ph}Dbf)$_2$] (5a) with 2 equiv. FcPF_6 at 80 K 55

Figure S-51. Frozen toluene EPR spectrum of the reaction of [(*m*-CF_3-Py)Fe_2(^{Ph}Dbf)$_2$] (6a) with 2 equiv. FcPF_6 at 80 K 56

Electrochemistry - Cyclic Voltammetry (CV) 57

Figure S-52. Cyclic voltammetry spectra for [Fe_2(^{Ph}Dbf)$_2$] (1a) 57

Figure S-53. Cyclic voltammetry spectra for [Fe_2(^{Ph}Dbf)$_2$] (1a) 57

Figure S-54. Cyclic voltammetry spectra for [Py_2Fe(^{Ph}Dbf)] (2a) 58

Figure S-55. Cyclic voltammetry spectra for [Py_2Fe(^{Ph}Dbf)] (2a) 58

Figure S-56. Cyclic voltammetry spectra for [(*p*-^{t}Bu-Py)$_2$Fe(^{Ph}Dbf)] (3a) 59

Figure S-57. Cyclic voltammetry spectra for [(*p*-^{t}Bu-Py)$_2$Fe(^{Ph}Dbf)] (3a) 59

Figure S-58. Cyclic voltammetry spectra for [(*p*-^{t}Bu-Py)$_2$Fe(^{Ph}Dbf)] (3a) 60

Figure S-59. Cyclic voltammetry spectra for [(*p*-CF_3-Py)$_2$Fe(^{Ph}Dbf)] (4a) 60

Figure S-60. Cyclic voltammetry spectra for [(*p*-CF_3-Py)$_2$Fe(^{Ph}Dbf)] (4a) 61

Figure S-61. Cyclic voltammetry spectra for [(*p*-CF_3-Py)$_2$Fe(^{Ph}Dbf)] (4a) 61

Figure S-62. Cyclic voltammetry spectra for [(*p*-CF_3-Py)Fe_2(^{Ph}Dbf)$_2$] (5a) 62

Figure S-63. Cyclic voltammetry spectra for [(*p*-CF_3-Py)Fe_2(^{Ph}Dbf)$_2$] (5a) 62

Figure S-64. Cyclic voltammetry spectra for [(*p*-CF_3-Py)Fe_2(^{Ph}Dbf)$_2$] (5a) 63

Figure S-65. Cyclic voltammetry spectra for [(*m*-CF_3Py)Fe_2(^{Ph}Dbf)$_2$] (6a) 63

Figure S-66. Cyclic voltammetry spectra for [(*m*-CF_3Py)Fe_2(^{Ph}Dbf)$_2$] (6a) 64

Figure S-67. Cyclic voltammetry spectra for [(*m*-CF_3Py)Fe_2(^{Ph}Dbf)$_2$] (6a) 64

Computational results 65

Figure S-68. Frontier molecular orbitals for [Py_2Fe(^{Ph}Dbf)] (2a) 65

Figure S-69. Spin density plot for [Py_2Fe(^{Ph}Dbf)] (2a) 65

Figure S-70. Natural transition orbitals for [(p-^{t}Bu-Py)$_2$Fe(^{Ph}Dbf)] (3a) illustrating the MLCT transitions from the non-bonding d_{yz} orbital into the 4-*tert*-butylpyridine lowest π* orbital. 66

Figure S-71. Natural transition orbitals for [(p-CF$_3$-Py)$_2$Fe(^{Ph}Dbf)] (4a) illustrating the MLCT transitions from the non-bonding d_{yz} orbital into the 4-trifluoromethylpyridine lowest π* orbital. 67

Table S-1. Representative bond metrics in 2a – 4a. 68

Table S-2. Pyridine bond metrics in 2a compared to those for free pyridine (reported in Å) 68

Table S-3. Optimized XYZ coordinates for [Py$_2$Fe(^{Ph}Dbf)] (2a) (in Å) 69

Table S-4. Optimized XYZ coordinates for [(p-^{t}Bu-Py)$_2$Fe(^{Ph}Dbf)] (3a) (in Å) 71

Table S-5. Optimized XYZ coordinates for [(p-CF$_3$-Py)$_2$Fe(^{Ph}Dbf)] (4a) (in Å) 73

Table S-6. Optimized XYZ coordinates for free pyridine (in Å) 75

Table S-7. Optimized XYZ coordinates for free pyridine radical anion (in Å) 75

X-Ray Diffraction Techniques. 77

Table S-8. X-ray diffraction experimental details 79

Figure S-72. Solid-state molecular structure for [Fe$_2$(^{Ph}Dbf)$_2$] (1a) 83

Figure S-73. Space-filling model of the solid-state molecular structure for [Fe$_2$(^{Ph}Dbf)$_2$] (1a) 83

Figure S-74. Solid-state molecular structure for [Py$_2$Fe(^{Ph}Dbf)] (2a) 84

Figure S-75. Solid-state molecular structure for [(p-^{t}Bu-Py)$_2$Fe(^{Ph}Dbf)] (3a) 85

Figure S-76. Solid-state molecular structure for [(p-CF$_3$-Py)$_2$Fe(^{Ph}Dbf)] (4a) 86

Figure S-77. Solid-state molecular structure for [(p-CF$_3$-Py)Fe$_2$(^{Ph}Dbf)$_2$] (5a) 87

Figure S-78. Solid-state molecular structure for [PyFe(^{Ph}Dbf)Cl] (2b) 88

Figure S-79. Solid-state molecular structure for [Py$_2$Fe(^{Ph}Dbf)][PF$_6$] (2c) 89

Figure S-80. Solid-state molecular structure for [(THF)$_2$Fe(^{Ph}Dbf)][PF$_6$] (7) 90

Table S-9. Relevant bond metrics for [Fe$_2$(^{Ph}Dbf)$_2$] (1a) 91

Table S-10. Reported g_{eff} values obtained via low temperature (80 K) EPR for Fe(III) species 91

Pyridine Lability Studies 92

Figure S-81. ^{1}H NMR of the isolated product (yellow) of the reaction of [Fe(^{Ph}Dbf)(Py)$_2$] (2a) and 4-*tert*-butylpyridine. 92

Figure S-82. ^{1}H NMR of the isolated product (purple) of the reaction of [Fe(^{Ph}Dbf)(Py)$_2$] (2a) and 4-trifluoromethylpyridine. 93

Figure S-83. ^{1}H NMR of the isolated product (yellow) of the reaction of [Fe(^{Ph}Dbf)(p-^{t}Bu-Py)$_2$] (3a) and pyridine. 94

Figure S-84. ^{1}H NMR of the isolated product (yellow) of the reaction of [Fe(^{Ph}Dbf)(p-^{t}Bu-Py)$_2$] (3a) and 4-trifluoromethylpyridine. 95

Figure S-85. ^{1}H NMR of the isolated product (orange) of the reaction of [Fe(^{Ph}Dbf)(p-CF_3-Py)$_2$] (4a) and pyridine. 96

Figure S-86. ^{1}H NMR of the isolated product (orange) of the reaction of [Fe(^{Ph}Dbf)(p-CF_3-Py)$_2$] (4a) and 4-*tert*-butylpyridine. 97

Figure S-87. ^{1}H NMR of the isolated product (orange) of the reaction of [Fe_2(^{Ph}Dbf)$_2$(m-CF_3-Py)] (6a) and pyridine. 98

Figure S-88. ^{1}H NMR of the isolated product (orange) of the reaction of [Fe_2(^{Ph}Dbf)$_2$(m-CF_3-Py)] (6a) and 4-*tert*-butylpyridine. 99

Figure S-89. ^{1}H NMR of the isolated product (orange) of the reaction of [Fe_2(^{Ph}Dbf)$_2$(m-CF_3-Py)] (6a) and 4-trifluoromethylpyridine. 100

Figure S-90. ^{1}H NMR of the product of the reaction of [Fe_2(^{Ph}Dbf)$_2$] (1a) and lutidine. 101

UV-Vis Spectra and Calibration Curves

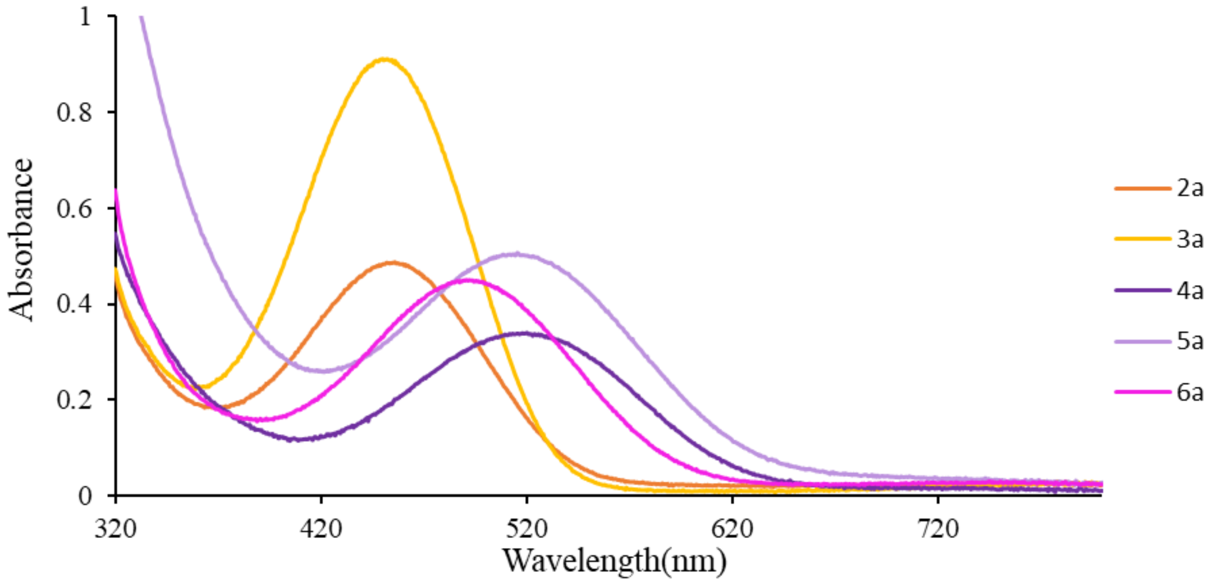

Figure S-1. Stacked UV-Vis spectra for [$Py_2Fe(^{Ph}Dbf)$] (**2a**), [(p-^tBu-Py)$_2Fe(^{Ph}Dbf)$] (**3a**), [(p-CF_3-Py)$_2Fe(^{Ph}Dbf)$] (**4a**), [(p-CF_3-Py)$Fe_2(^{Ph}Dbf)_2$] (**5a**), and [(m-CF_3-Py)$Fe_2(^{Ph}Dbf)_2$] (**6a**) collected in benzene with background and blank corrections. λmax for each complex is reported as 452 nm (**2a**, Orange), 450 nm (**3a**; Yellow), 517 nm (**4a**; Purple), 515 nm (**5a**; Purple) and 491 nm (**6a**; Pink).

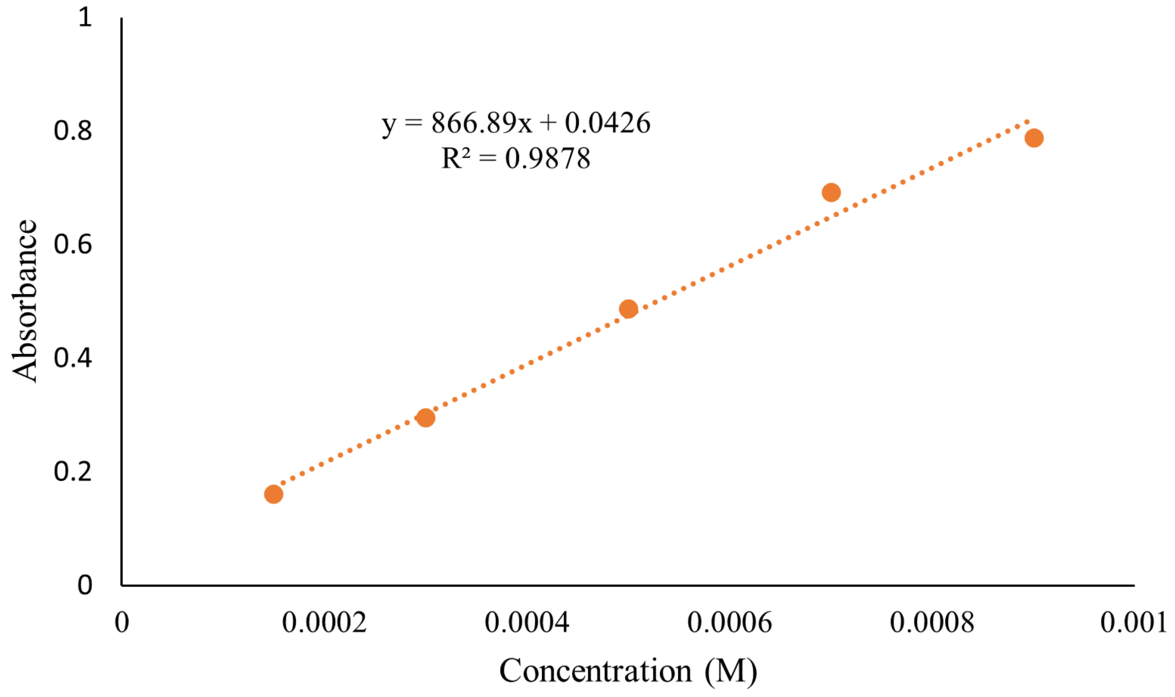

Figure S-2. Calibration curve for [Py$_2$Fe(^{Ph}Dbf)] (**2a**) collected in benzene with background and blank corrections. The R squared value of 0.9878 reported suggests a strong linear relationship between concentration and absorbance. The molar extinction coefficient derived from the slope of the linear regression line was calculated as 866.89 $M^{-1}cm^{-1}$.

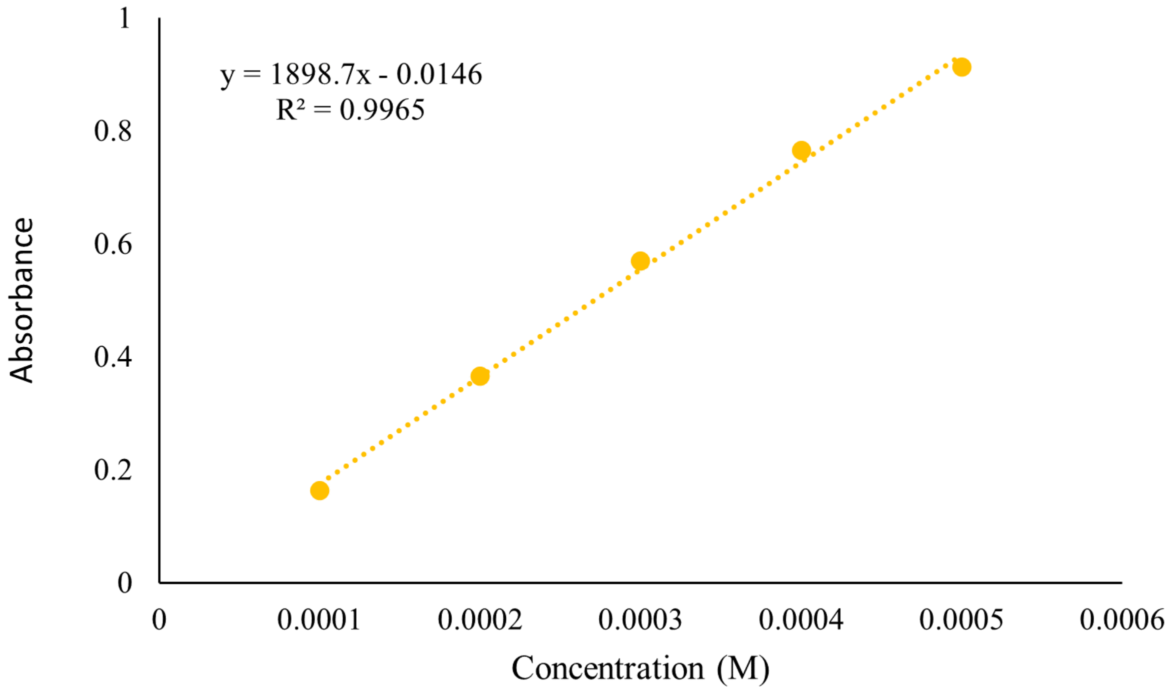

Figure S-3. Calibration curve for [(*p*-^{t}Bu-Py)$_2$Fe(^{Ph}Dbf)] (**3a**) collected in benzene with background and blank corrections. The R squared value of 0.9965 reported suggests a strong linear relationship between concentration and absorbance. The molar extinction coefficient derived from the slope of the linear regression line was calculated as 1898.7 $M^{-1}cm^{-1}$.

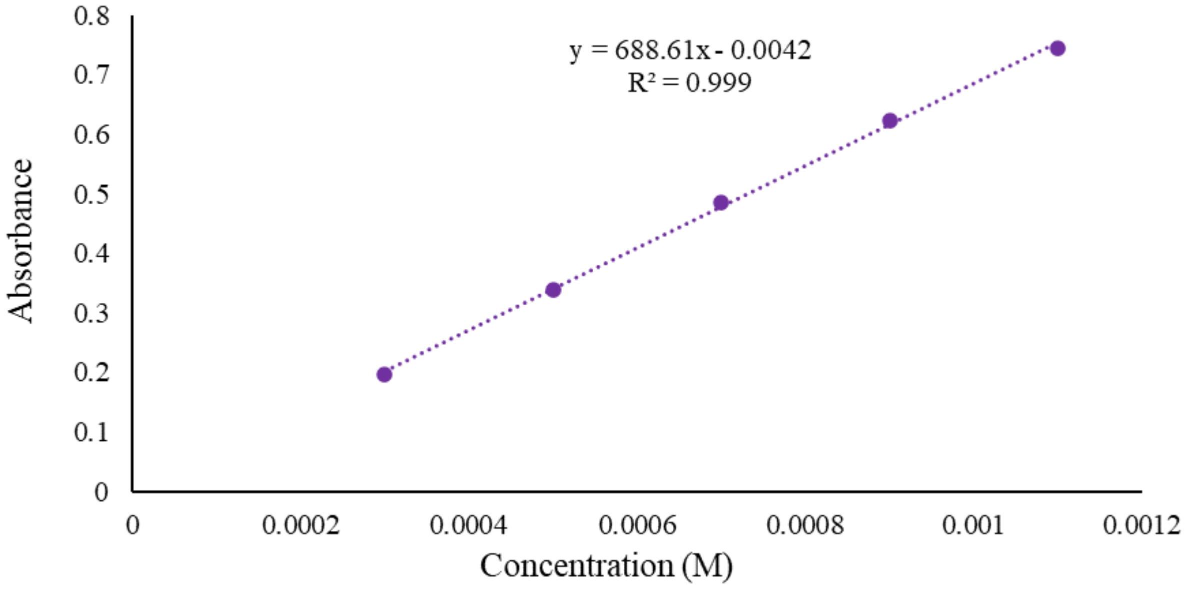

Figure S-4. Calibration curve for [(*p*-CF_3-Py)$_2$Fe(^{Ph}Dbf)] (**4a**) collected in benzene with background and blank corrections. The R squared value of 0.999 reported suggests a strong linear relationship between concentration and absorbance. The molar extinction coefficient derived from the slope of the linear regression line was calculated as 688.61 $M^{-1}cm^{-1}$.

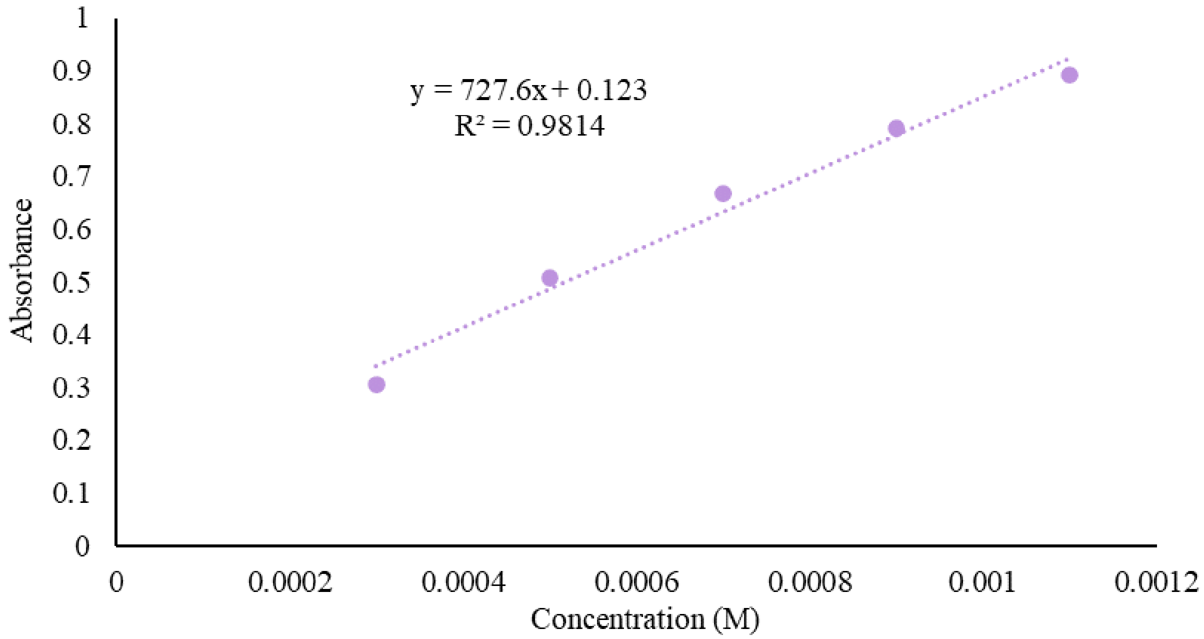

Figure S-5. Calibration curve for [(*p*-CF_3-Py)Fe_2(^{Ph}Dbf)$_2$] (**5a**) collected in benzene with background and blank corrections. The R squared value of 0.9814 reported suggests a strong linear relationship between concentration and absorbance. The molar extinction coefficient derived from the slope of the linear regression line was calculated as 727.6 $M^{-1}cm^{-1}$.

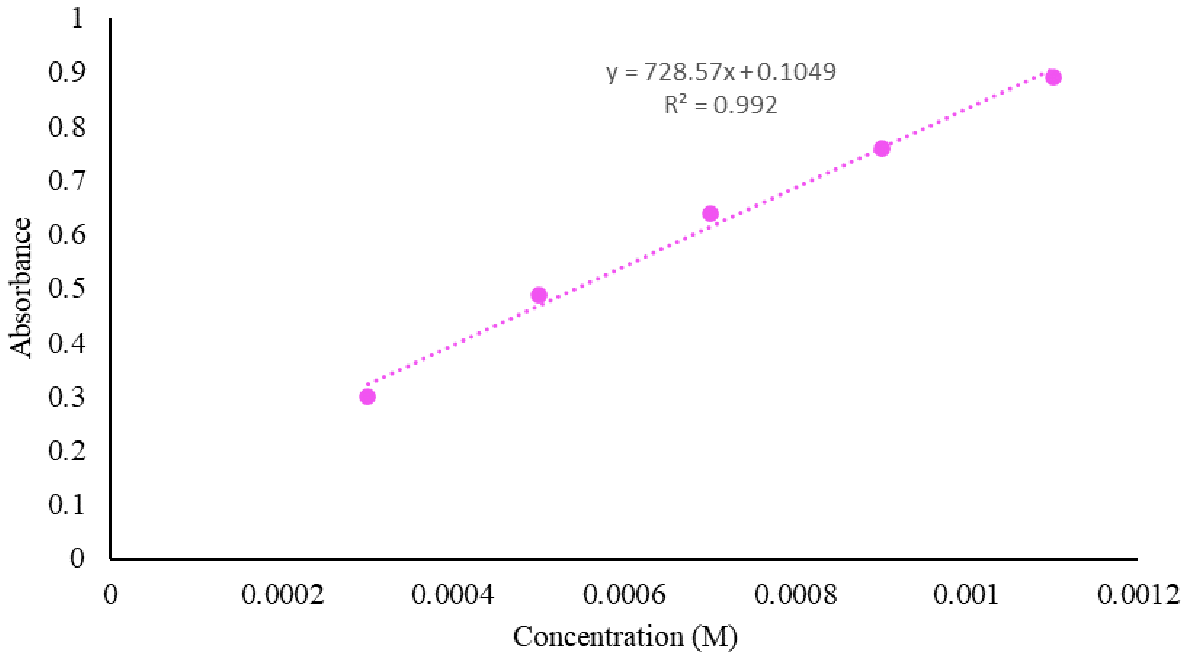

Figure S-6. Calibration curve for [(*m*-CF_3-Py)Fe_2(^{Ph}Dbf)$_2$] (**6a**) collected in benzene with background and blank corrections. The R squared value of 0.992 reported suggests a strong linear relationship between concentration and absorbance. The molar extinction coefficient derived from the slope of the linear regression line was calculated as 728.57 $M^{-1}cm^{-1}$.

Zero-field ^{57}Fe Mössbauer Spectra

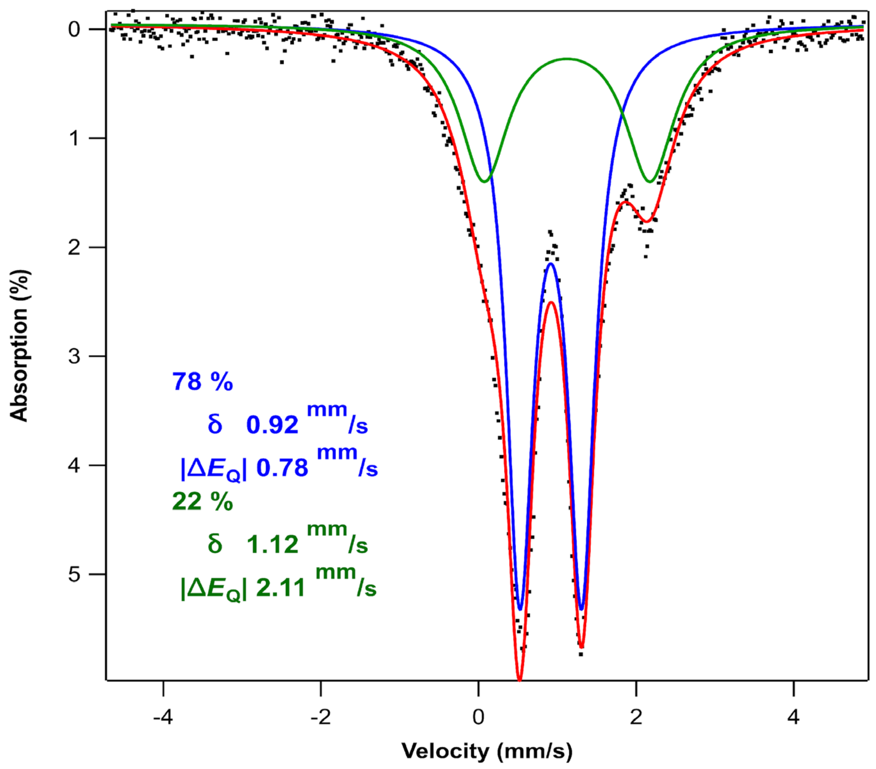

Figure S-7. Zero-field ^{57}Fe Mössbauer of [$Fe_2(^{Ph}Dbf)_2$] (**1a**) collected at 90 K. Isomer shift and quadrupole splitting are referenced to Fe foil at room temperature. Experimental parameters 78 % δ = 0.92 mm/s, $|\Delta E_Q|$ = 0.78 mm/s and 22% δ = 1.12 mm/s, $|\Delta E_Q|$ = 2.11 mm/s. Parameters calculated via single-point DFT: 50% δ = 0.87 mm/s, ΔE_Q = 0.601 mm/s; 50% δ = 1.00 mm/s, ΔE_Q = –1.099 mm/s

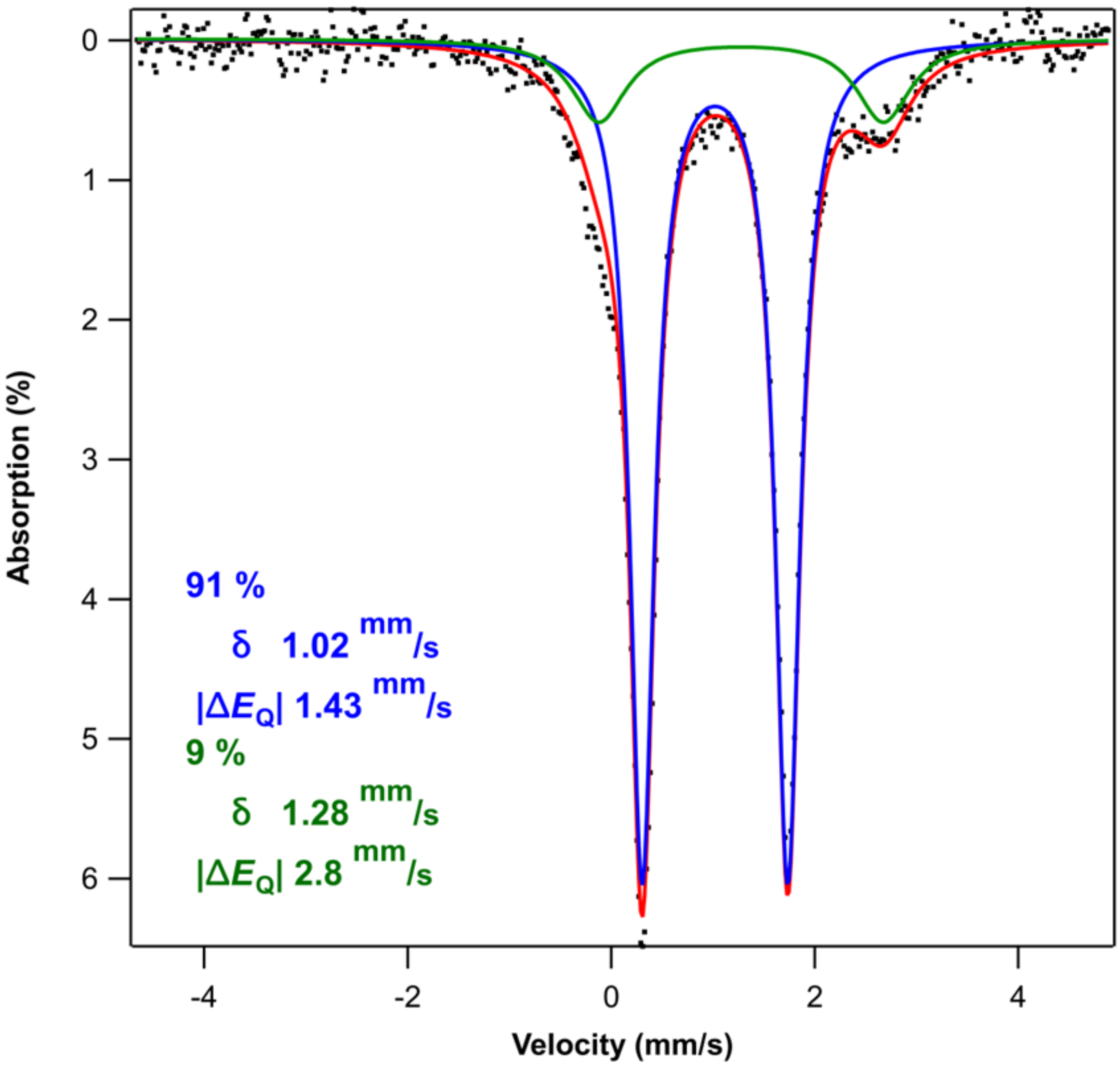

Figure S-8. Zero-field ^{57}Fe Mössbauer of [$Py_2Fe(^{Ph}Dbf)$] (**2a**) collected at 90 K. Isomer shift and quadrupole splitting are referenced to Fe foil at room temperature. Experimental parameters 91% δ = 1.02 mm/s, $|\Delta E_Q|$ = 1.43 mm/s and 9% δ = 1.28 mm/s, $|\Delta E_Q|$ = 2.8 mm/s. Parameters calculated via single-point DFT: 100% δ = 1.00 mm/s, ΔE_Q = 1.082 mm/s.

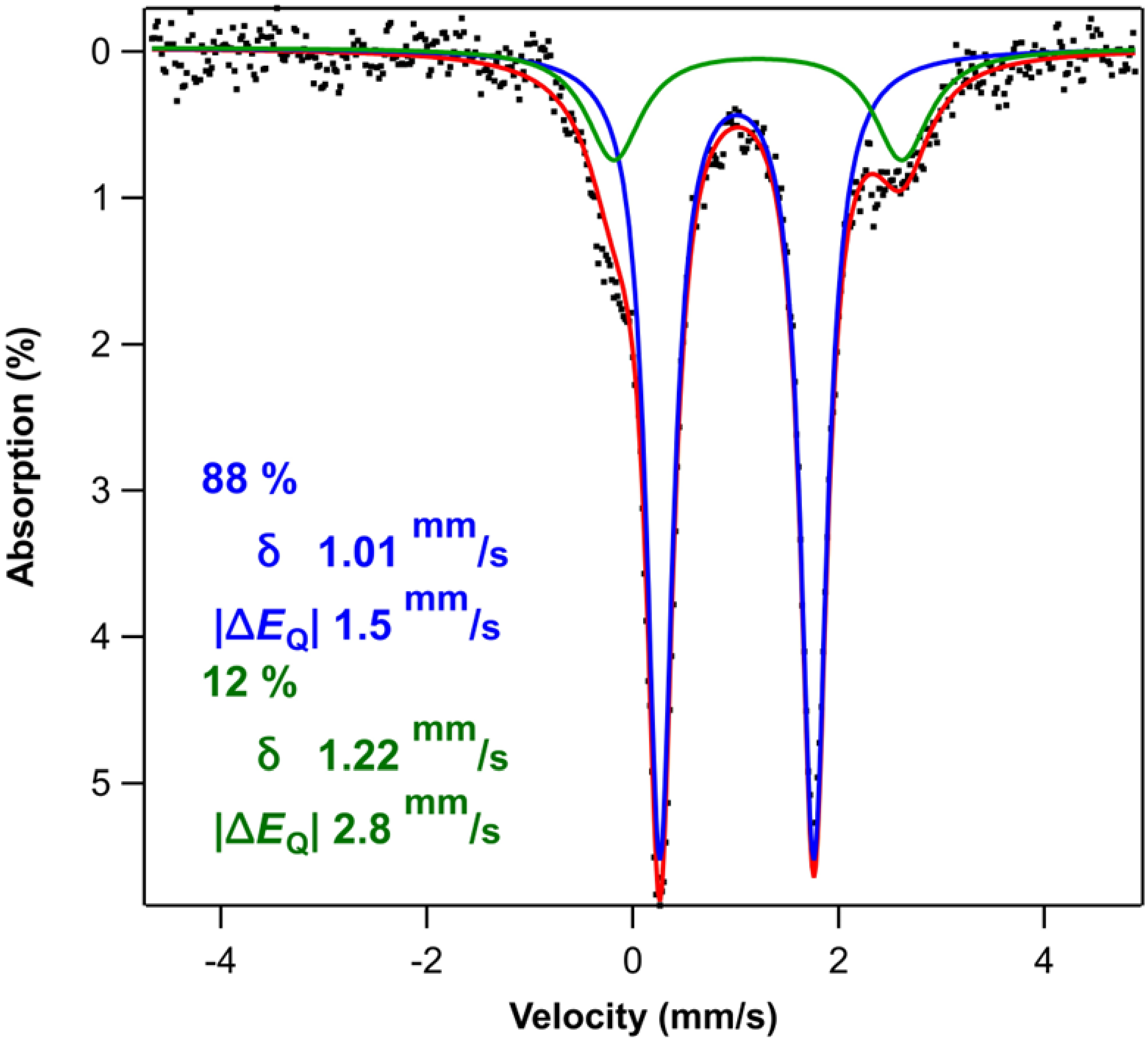

Figure S-9. Zero-field ^{57}Fe Mössbauer of [(^{t}BuPy)$_2$Fe(^{Ph}Dbf)] (**3a**) collected at 90 K. Isomer shift and quadrupole splitting are referenced to Fe foil at room temperature. Experimental parameters 88% δ = 1.01 mm/s, $|\Delta E_Q|$ = 1.5 mm/s and 12% δ = 1.22 mm/s, $|\Delta E_Q|$ = 2.8 mm/s. Parameters calculated via single-point DFT: 100% δ = 1.00 mm/s, ΔE_Q = 1.093 mm/s.

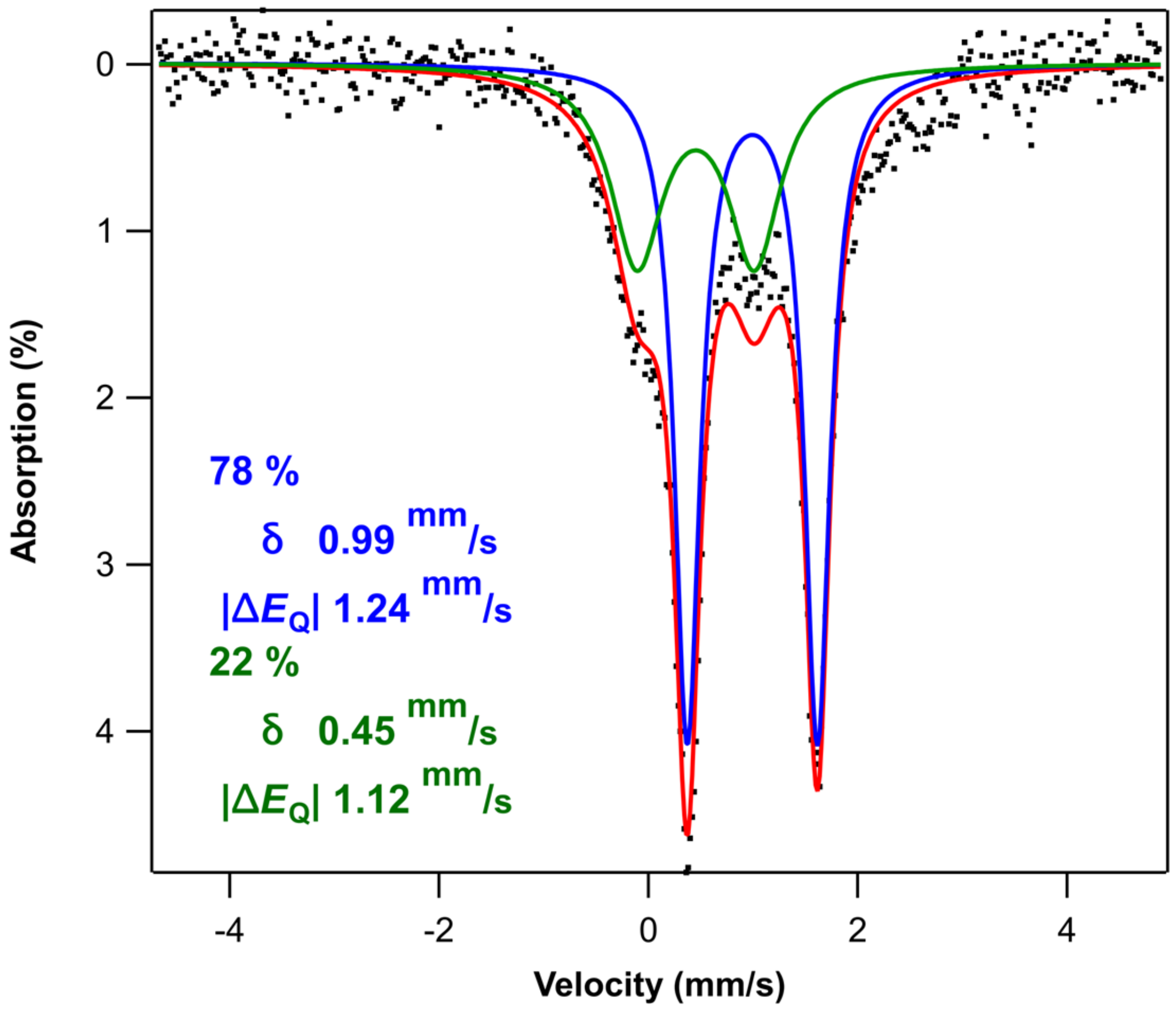

Figure S-10. Zero-field ^{57}Fe Mössbauer of [(*p*-$CF_3Py)_2Fe(^{Ph}Dbf)$] (**4a**) collected at 90 K. Isomer shift and quadrupole splitting are referenced to Fe foil at room temperature. Experimental parameters 78% $\delta = 0.99$ mm/s, $|\Delta E_Q| = 1.24$ mm/s and 22% $\delta = 0.45$ mm/s, $|\Delta E_Q| = 1.12$ mm/s. Parameters calculated via single-point DFT: 100% $\delta = 0.97$ mm/s, $\Delta E_Q = 0.862$ mm/s.

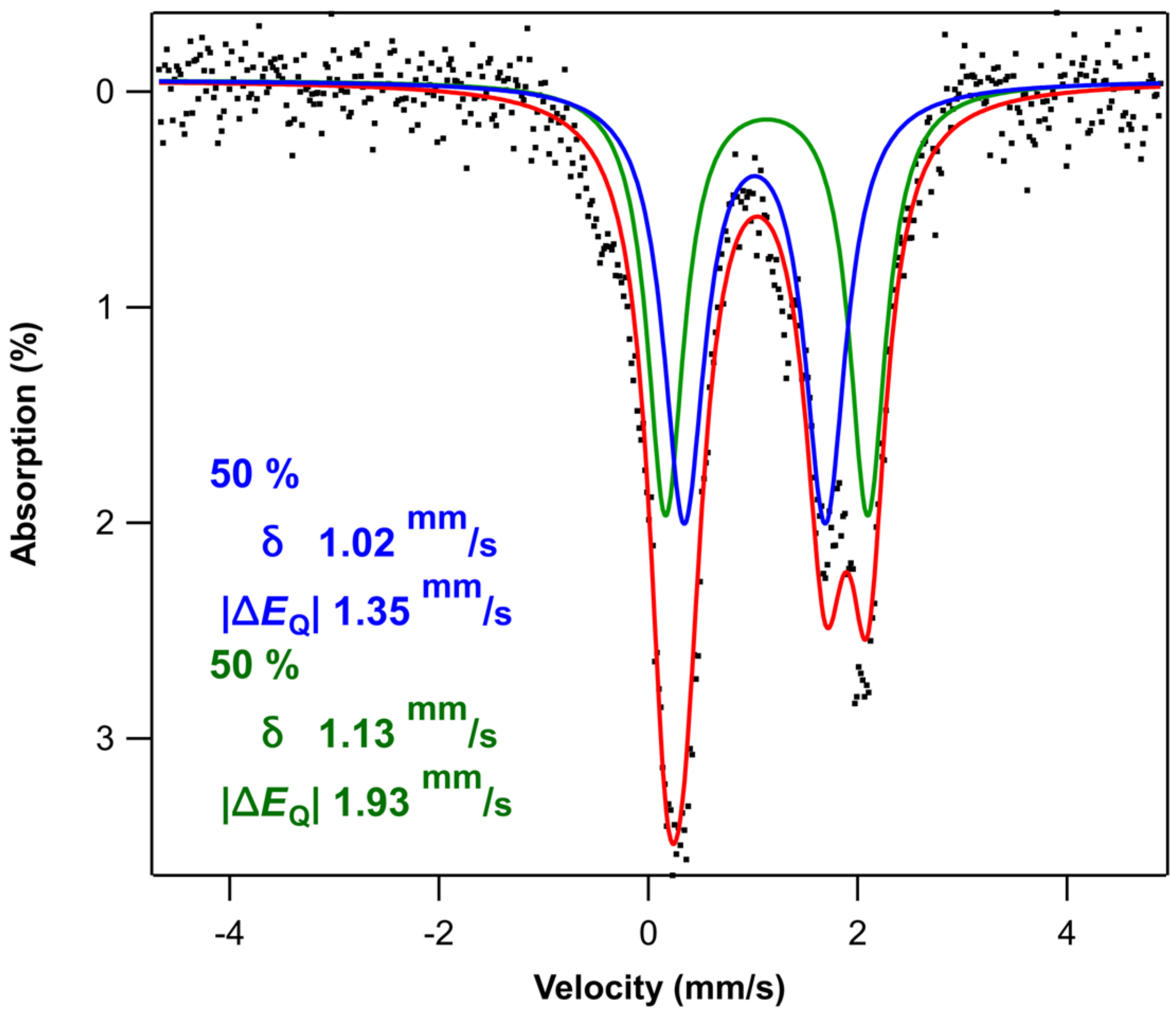

Figure S-11. Zero-field ^{57}Fe Mössbauer of [(*m*-CF_3Py)Fe_2(^{Ph}Dbf)$_2$] (**6a**) collected at 90 K. Isomer shift and quadrupole splitting are referenced to Fe foil at room temperature. Experimental parameters 50% δ = 1.02 mm/s, |ΔE_Q| = 1.35 mm/s and 50% δ = 1.13 mm/s, |ΔE_Q| = 1.93 mm/s.

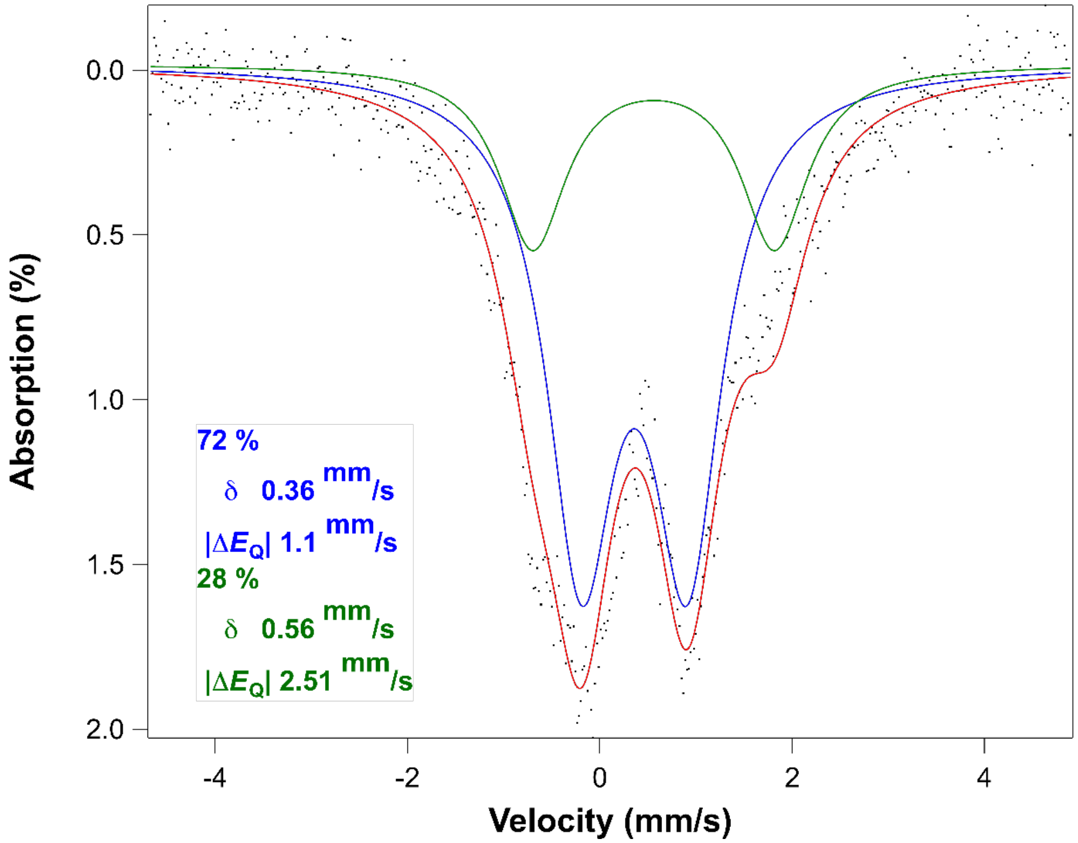

Figure S-12. Zero-field ^{57}Fe Mössbauer of [Py$_2$Fe(^{Ph}Dbf)Cl] (**2b**) collected at 90 K. Isomer shift and quadrupole splitting are referenced to Fe foil at room temperature. Experimental parameters 72% δ = 0.36 mm/s, $|\Delta E_Q|$ = 1.1 mm/s and 28% δ = 0.56 mm/s, $|\Delta E_Q|$ = 2.51 mm/s. Parameters calculated via single-point DFT: 100% δ = 0.34 mm/s, ΔE_Q = –1.982 mm/s.

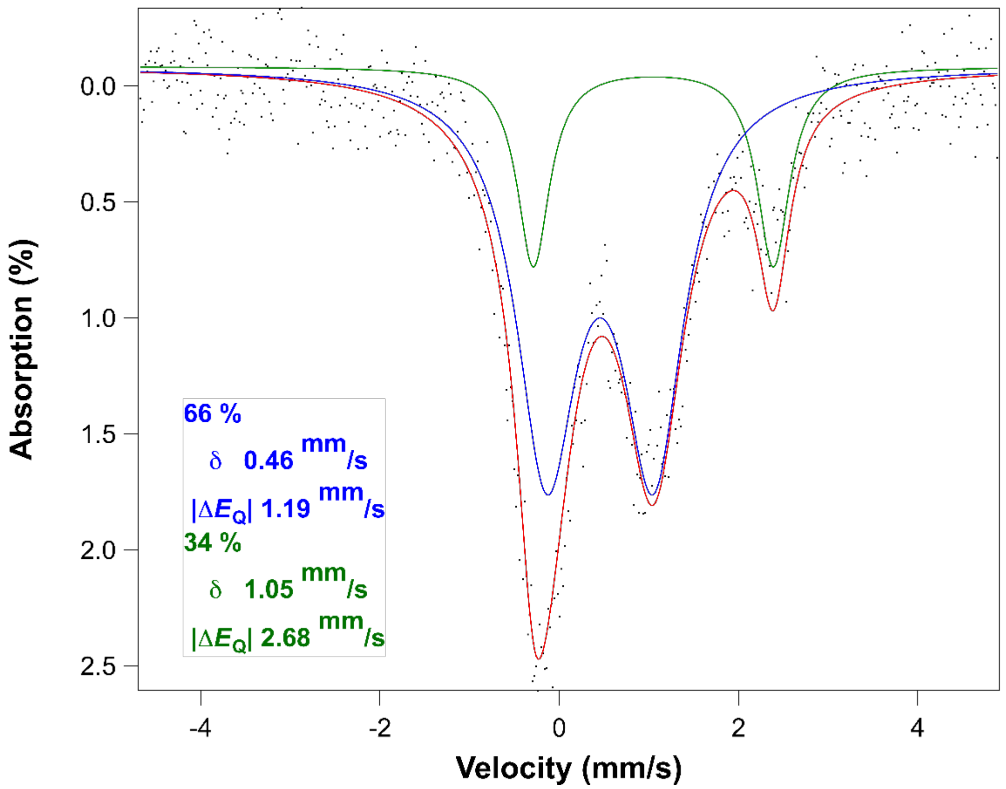

Figure S-13. Zero-field ^{57}Fe Mössbauer of the reaction of [(*p*-CF_3-Py)Fe_2(^{Ph}Dbf)$_2$] (**5a**) with trityl chloride collected at 90 K. Isomer shift and quadrupole splitting are referenced to Fe foil at room temperature. Experimental parameters 66% δ = 0.46 mm/s, $|\Delta E_Q|$ = 1.19 mm/s and 34% δ = 1.05 mm/s, $|\Delta E_Q|$ = 2.68 mm/s.

Nuclear Magnetic Resonance Spectra

All ^{19}F NMR were processed with the improved arPLS baseline correction in MestreNova in order to diminish the inherent signal of the Teflon probe to improve the signal to noise ratio.

.

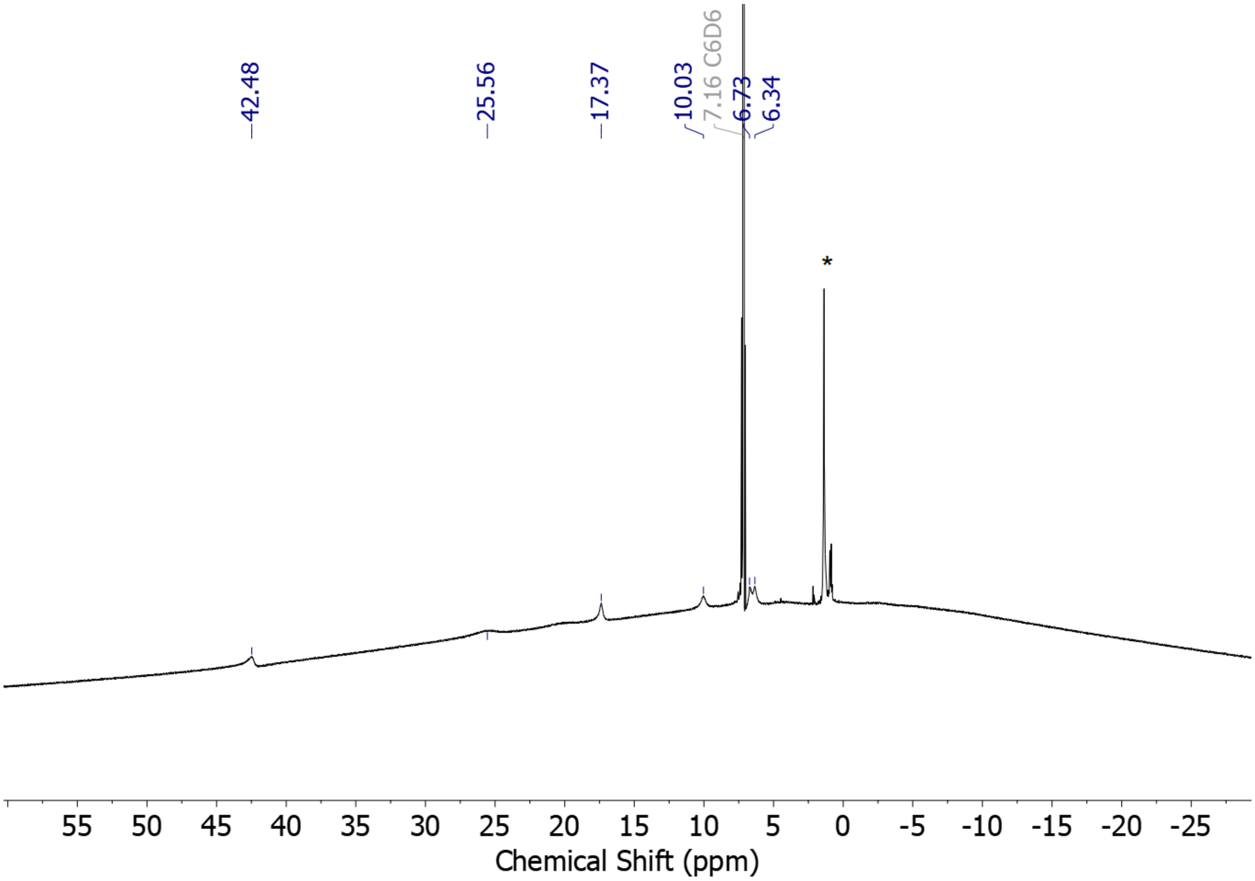

Figure S-14. ^{1}H NMR spectra for paramagnetic $[Fe_2(^{Ph}Dbf)_2]$ (**1a**). * indicates hexanes remaining from the wash (hexanes; δ 1.28 (br. s) and 0.88 (br. s)).

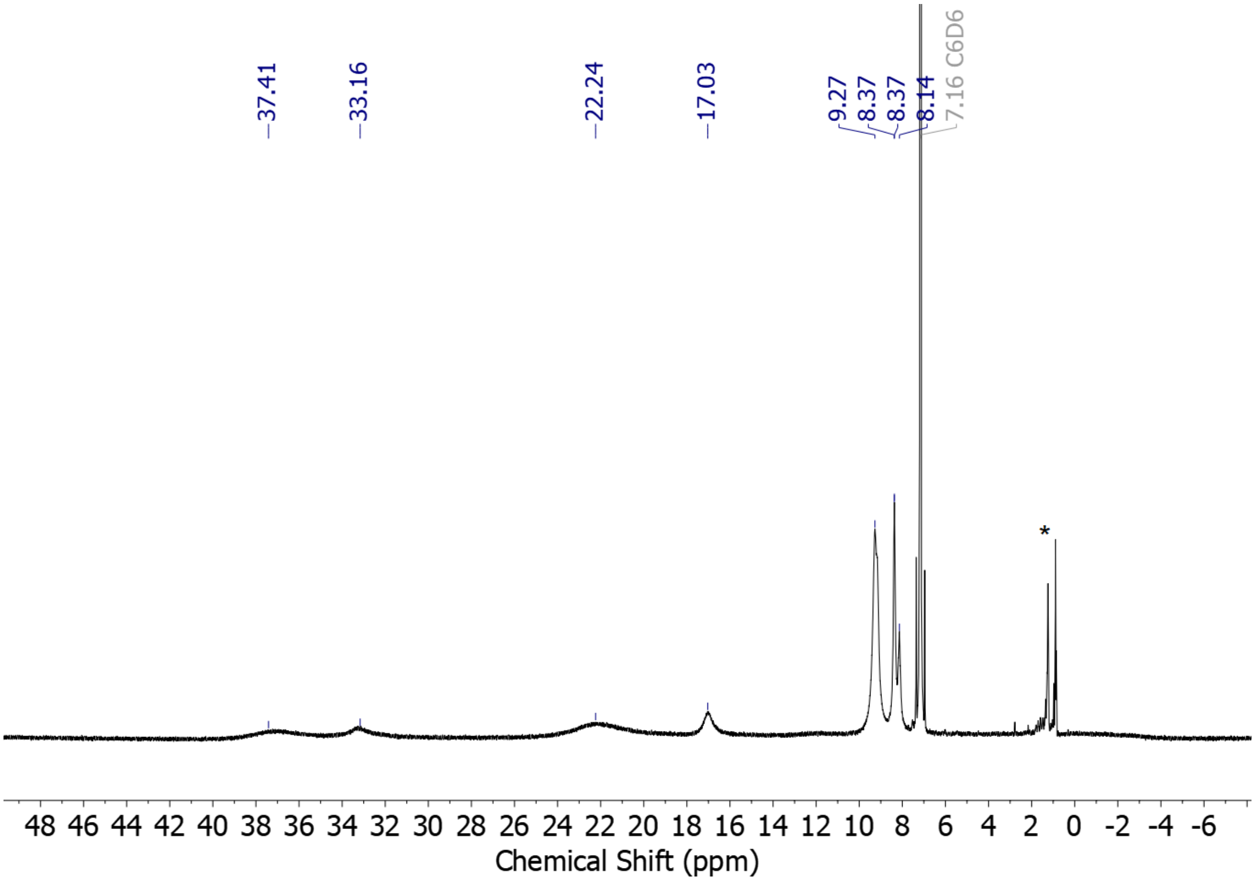

Figure S-15. ^{1}H NMR spectra for paramagnetic [$Py_2Fe(^{Ph}Dbf)$] (**2a**). *indicates minor organic impurities remaining from the wash (hexanes).

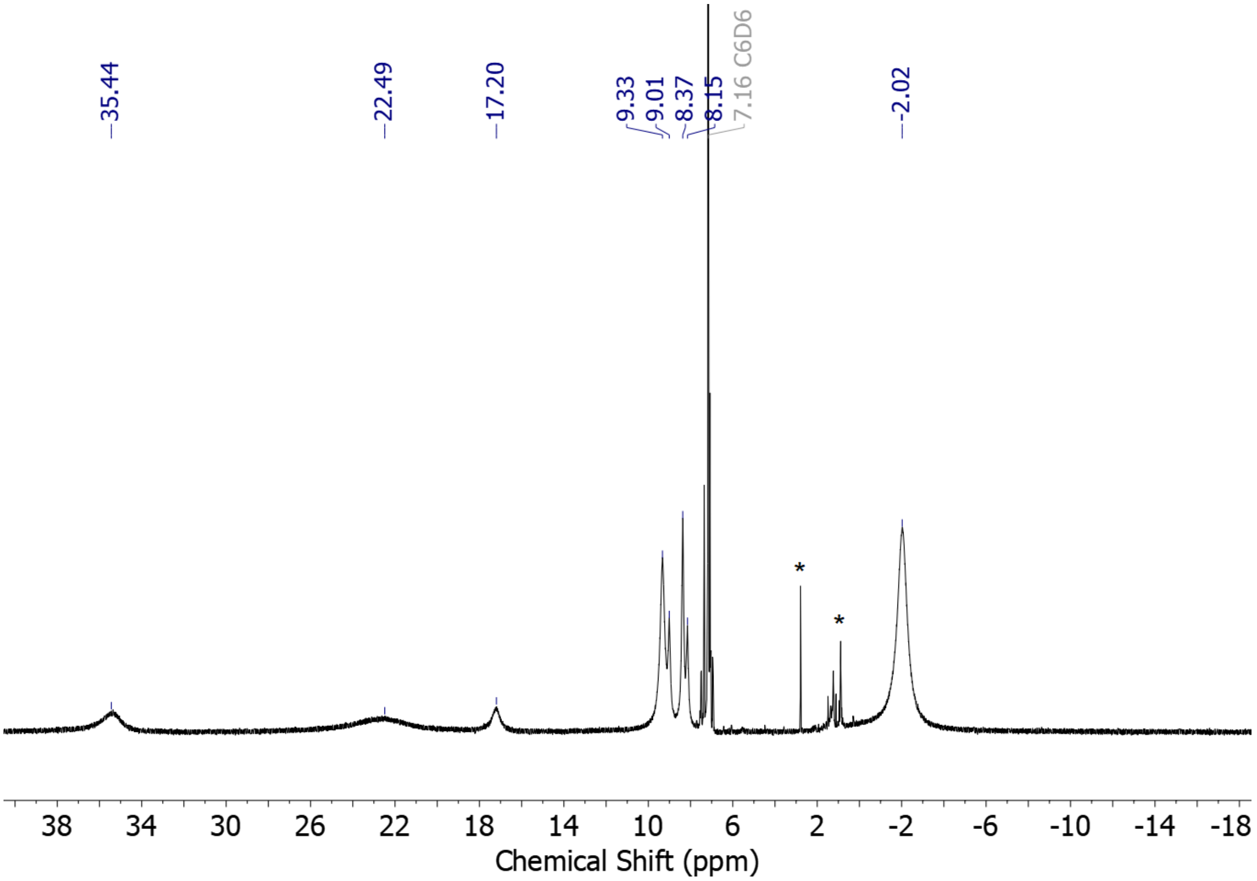

Figure S-16. ^{1}H NMR spectra for paramagnetic [(*p*-^{t}Bu-Py)$_2$Fe(^{Ph}Dbf)] (**3a**). *indicates minor organic impurities remaining from the wash (free ligand/hexanes). Note aromatic ligand peaks near the C_6D_6 signal were not marked for clarity.

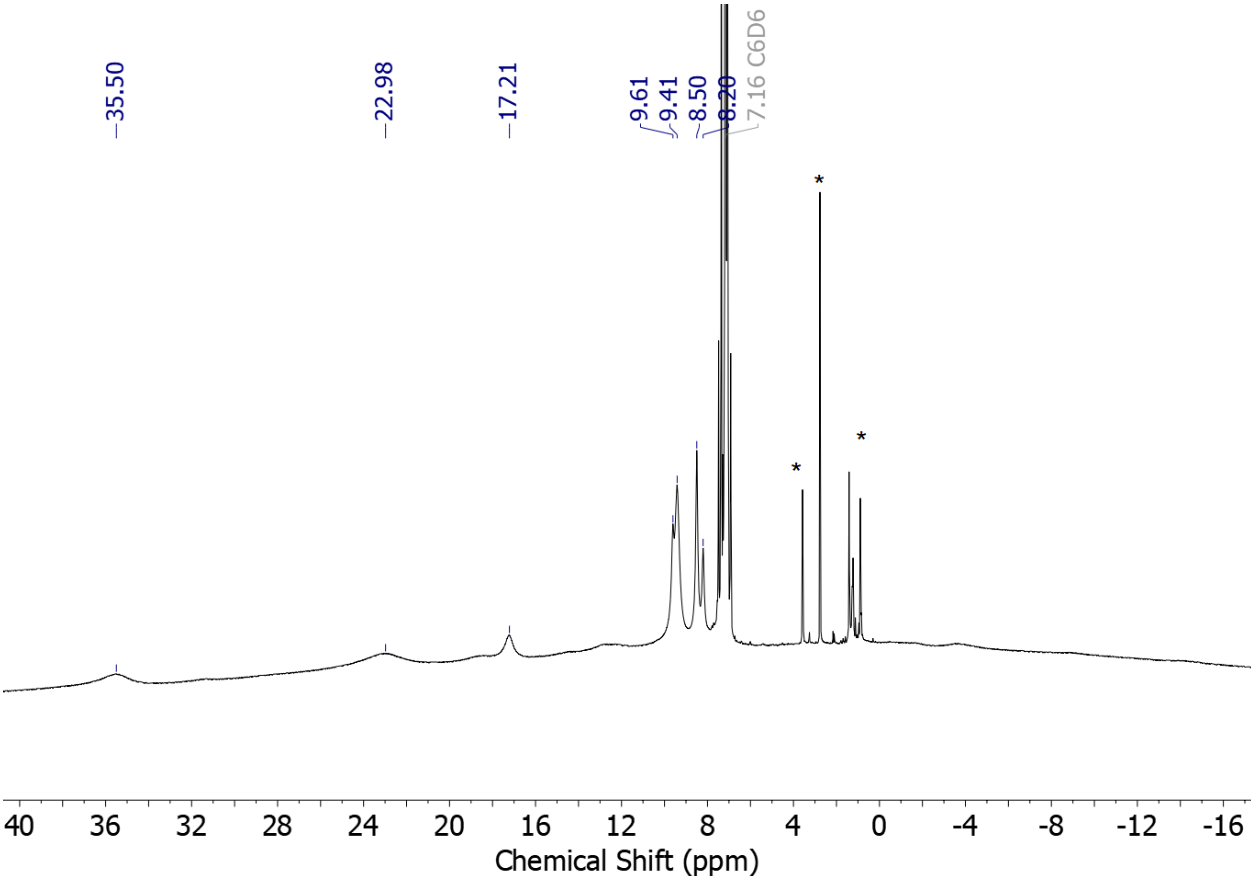

Figure S-17. ^{1}H NMR spectra for paramagnetic [(*p*-CF_3-Py)$_2$Fe(^{Ph}Dbf)] (**4a**). * indicates minor organic impurities remaining from the wash (free ligand/THF/hexanes). Note aromatic ligand peaks near the C_6D_6 signal were not marked for clarity.

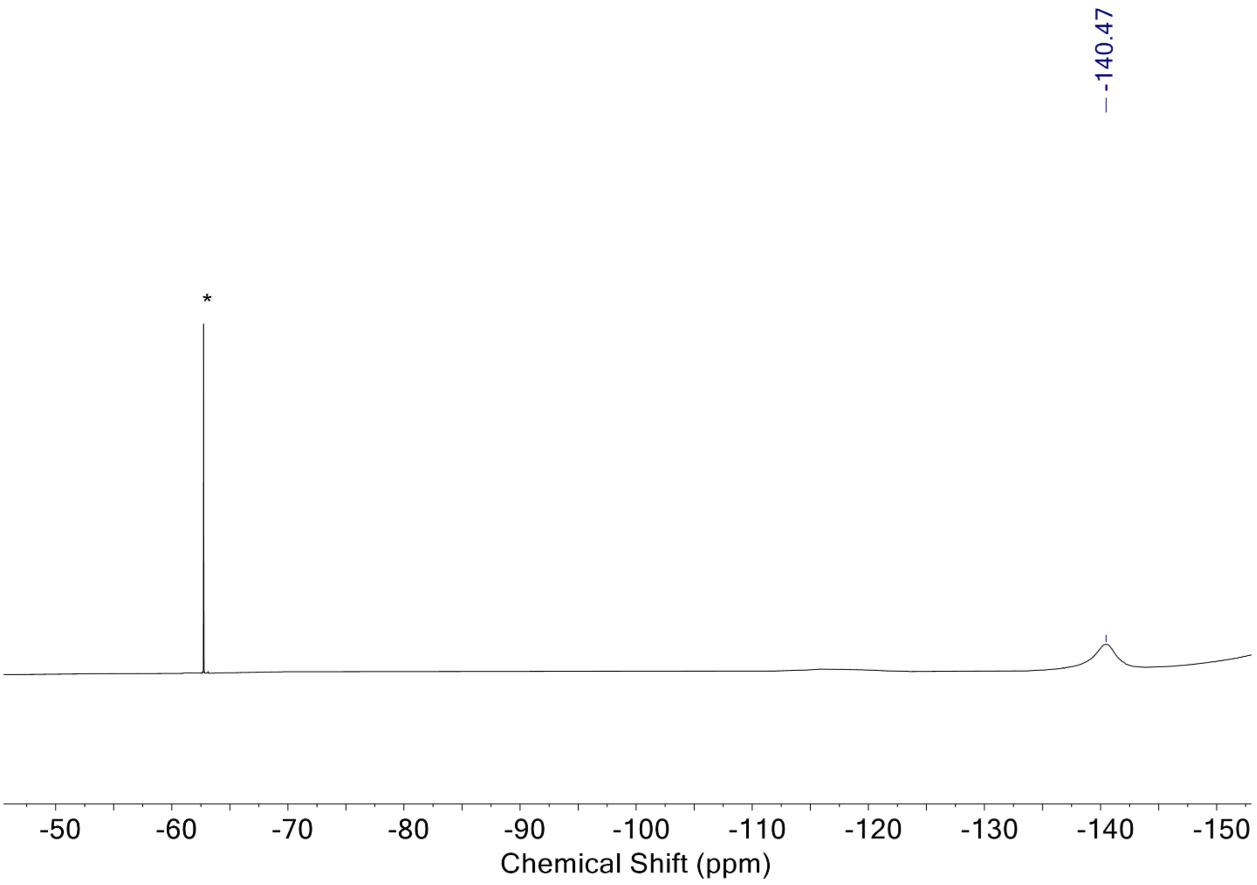

Figure S-18. ^{19}F NMR spectra for paramagnetic [(*p*-CF_3-Py)$_2$Fe(^{Ph}Dbf)] (**4a**) referenced to trifluorotoluene (-62.74 ppm).

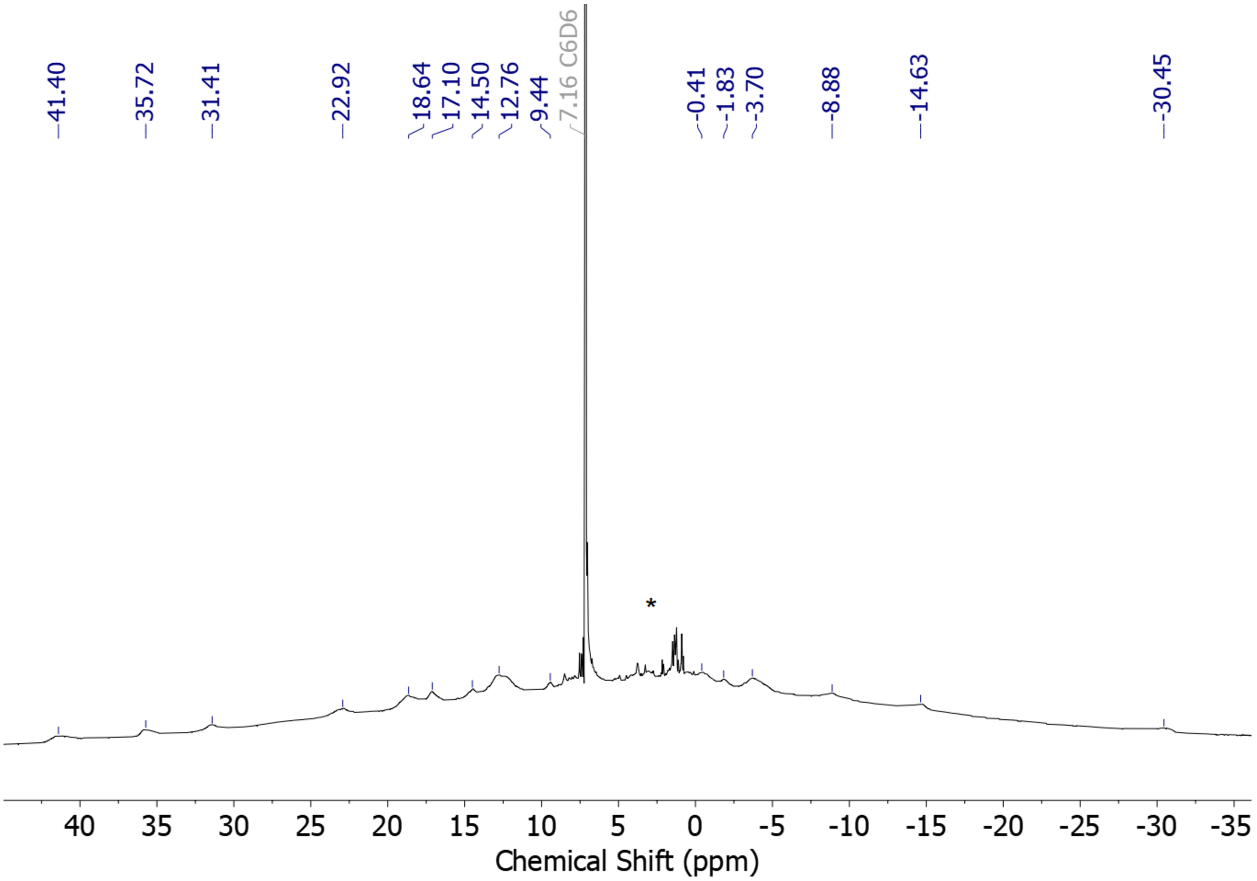

Figure S-19. ^{1}H NMR spectra for paramagnetic [(p-CF_3-Py)Fe_2(^{Ph}Dbf)$_2$] (**5a**). * indicates minor organic impurities remaining from the wash (free ligand/hexanes).

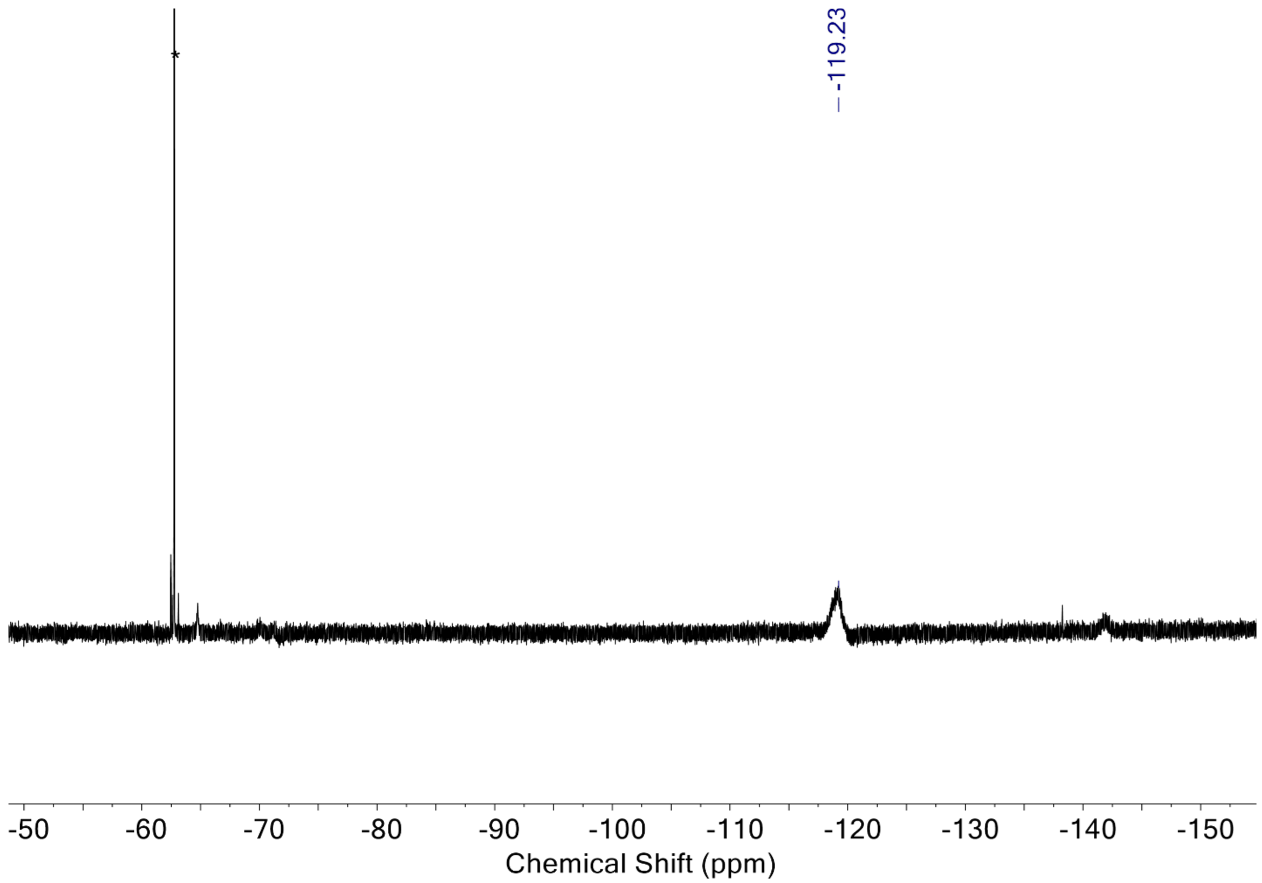

Figure S-20. ^{19}F NMR spectra for paramagnetic [(*p*-CF_3-Py)Fe_2(^{Ph}Dbf)$_2$] (**5a**) referenced to trifluorotoluene (-62.74 ppm).

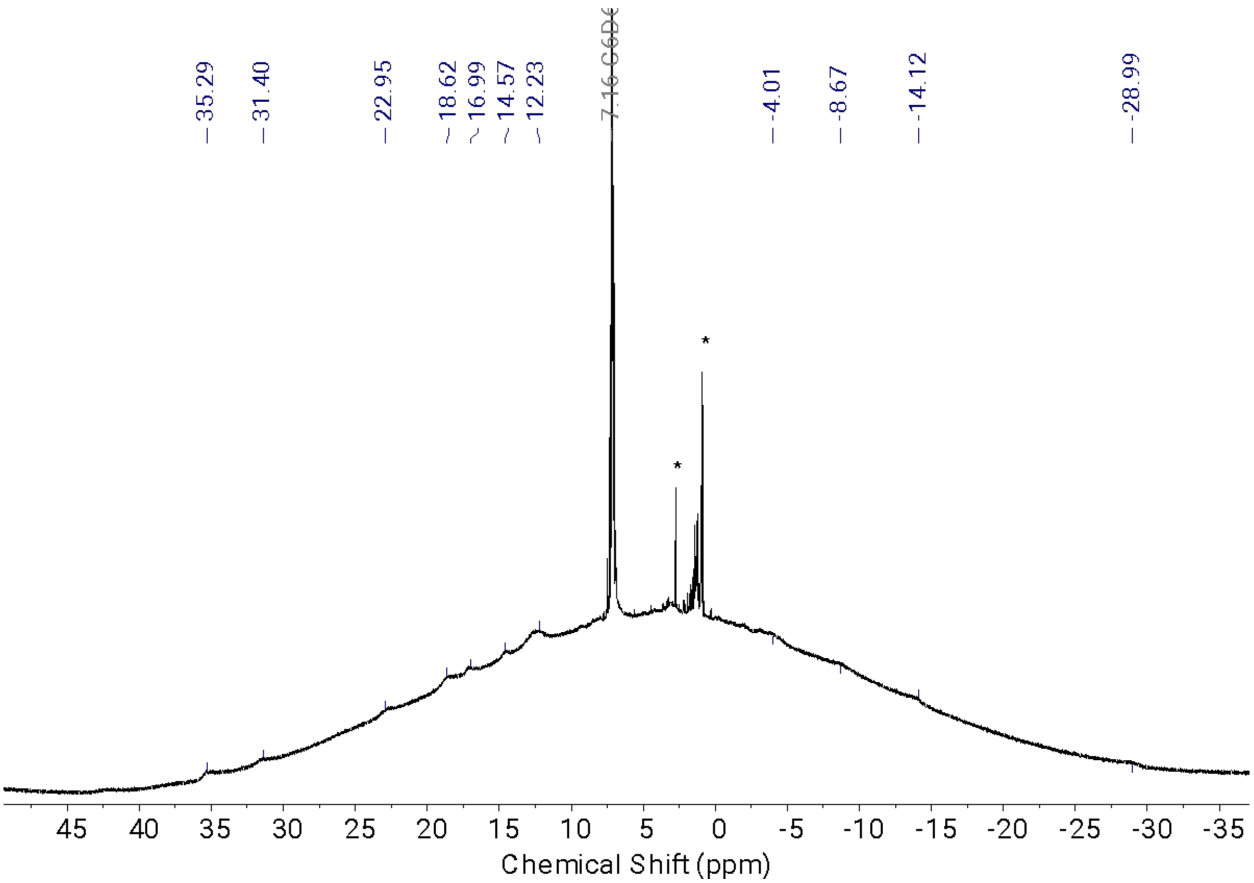

Figure S-21. ^{1}H NMR spectra for paramagnetic [(m-CF_3-Py)Fe_2(^{Ph}Dbf)$_2$] (**6a**). * indicates minor organic impurities remaining from the wash (free ligand/hexanes).

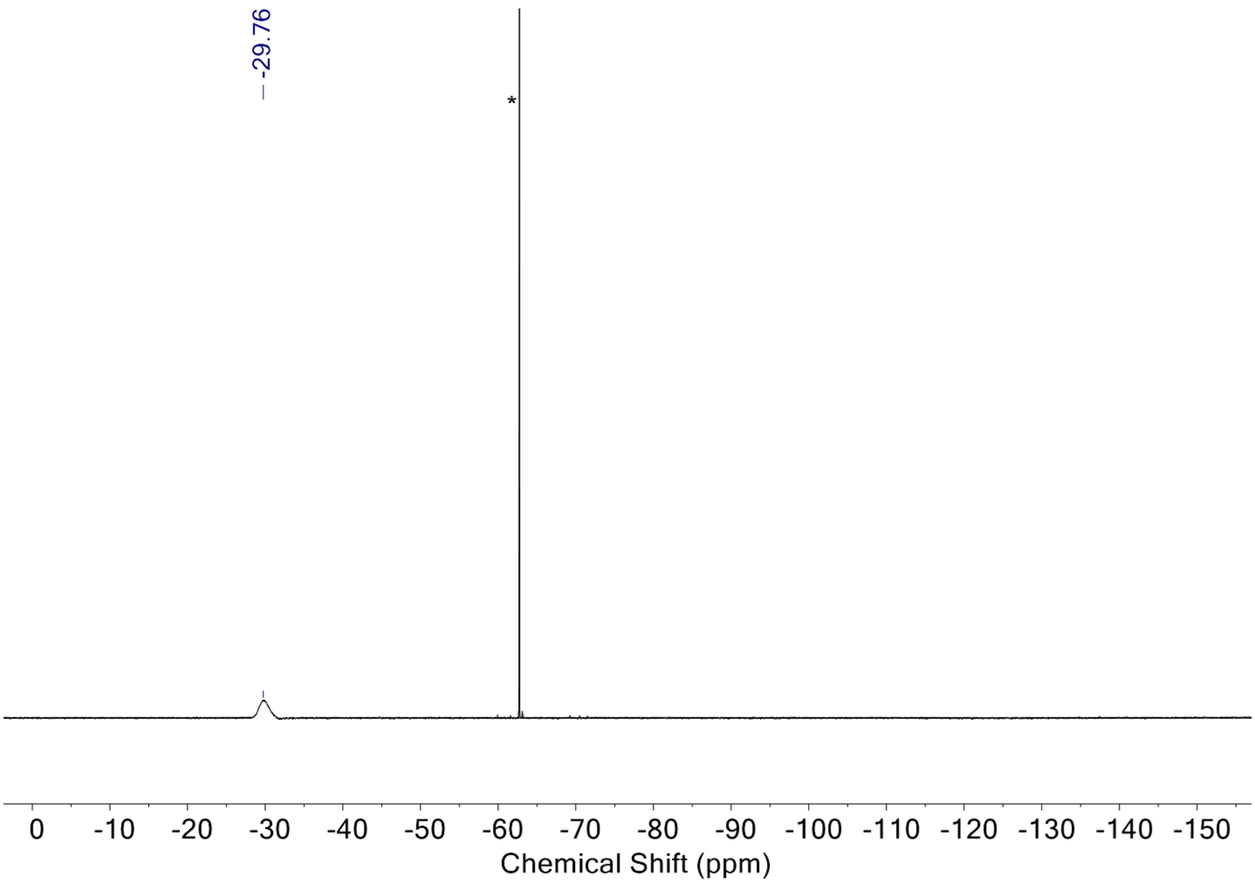

Figure S-22. ^{19}F NMR spectra for paramagnetic [(*m*-CF_3-Py)Fe_2(^{Ph}Dbf)$_2$] (**6a**) referenced to trifluorotoluene (-62.74 ppm).

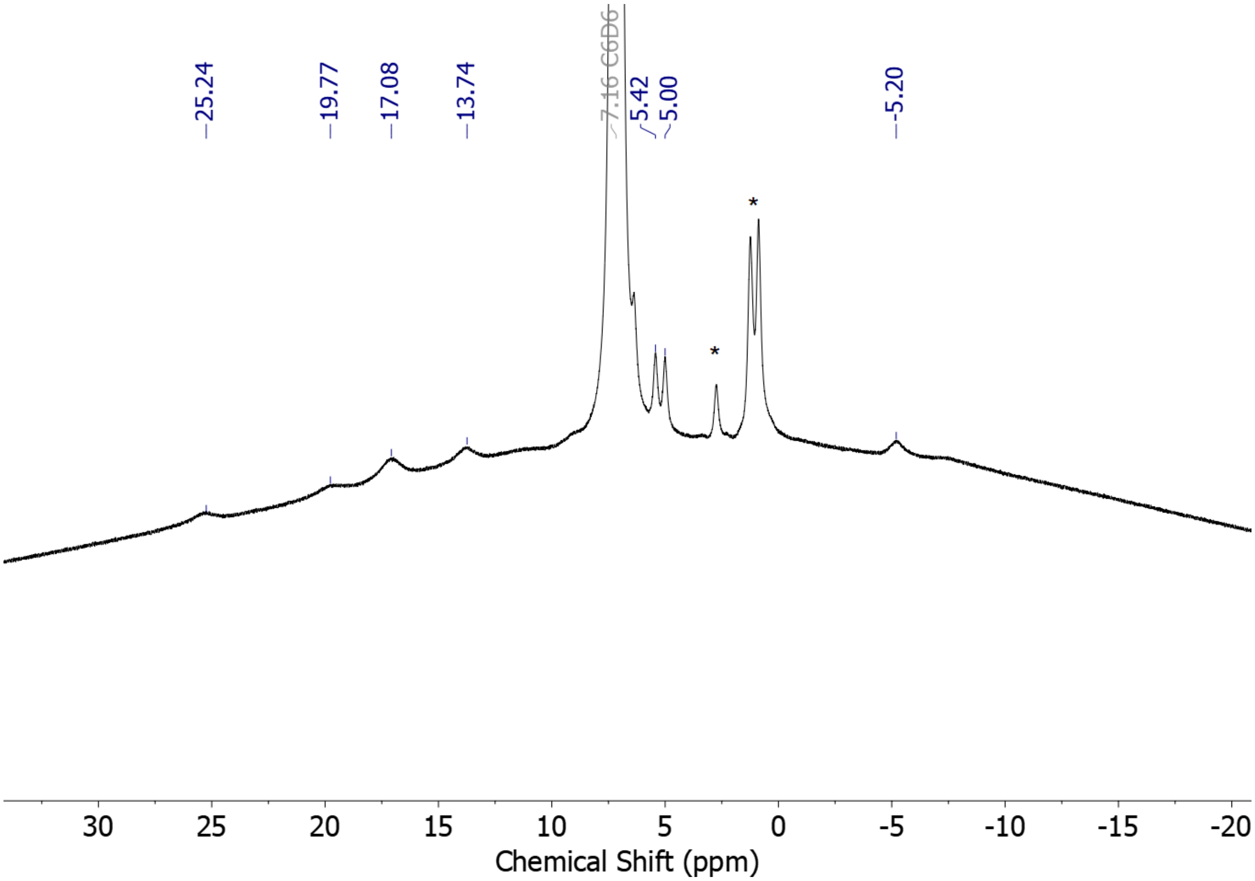

Figure S-23. ^{1}H NMR spectra for paramagnetic $[Fe_2(^{Ph}Dbf)_2Cl_2]$ (**1b**). * indicates minor organic impurities remaining from the wash (free ligand/hexanes).

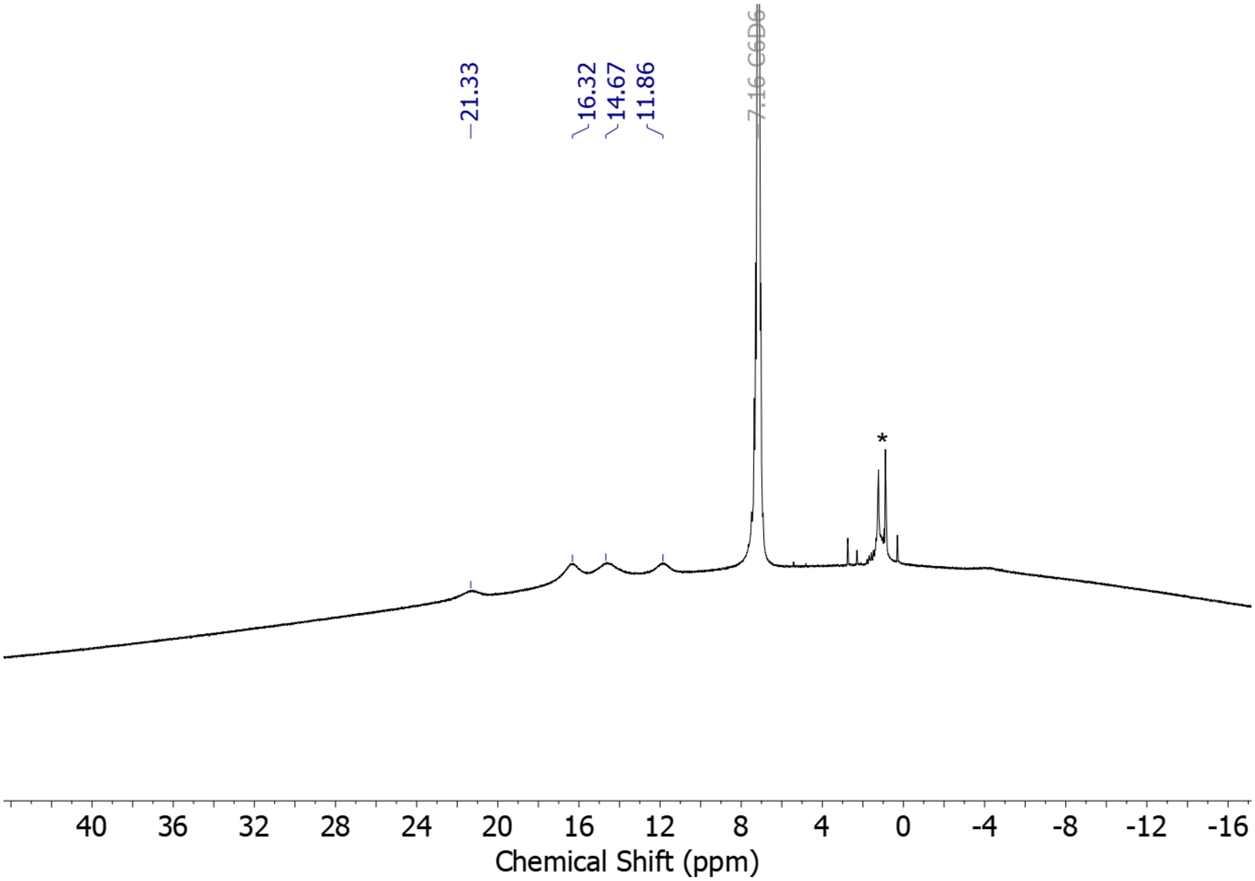

Figure S-24. ^{1}H NMR spectra for paramagnetic [PyFe(^{Ph}Dbf)Cl] (**2b**). * indicates minor organic impurities remaining from the wash (hexanes). Note aromatic ligand peaks near the C_6D_6 signal were not marked for clarity.

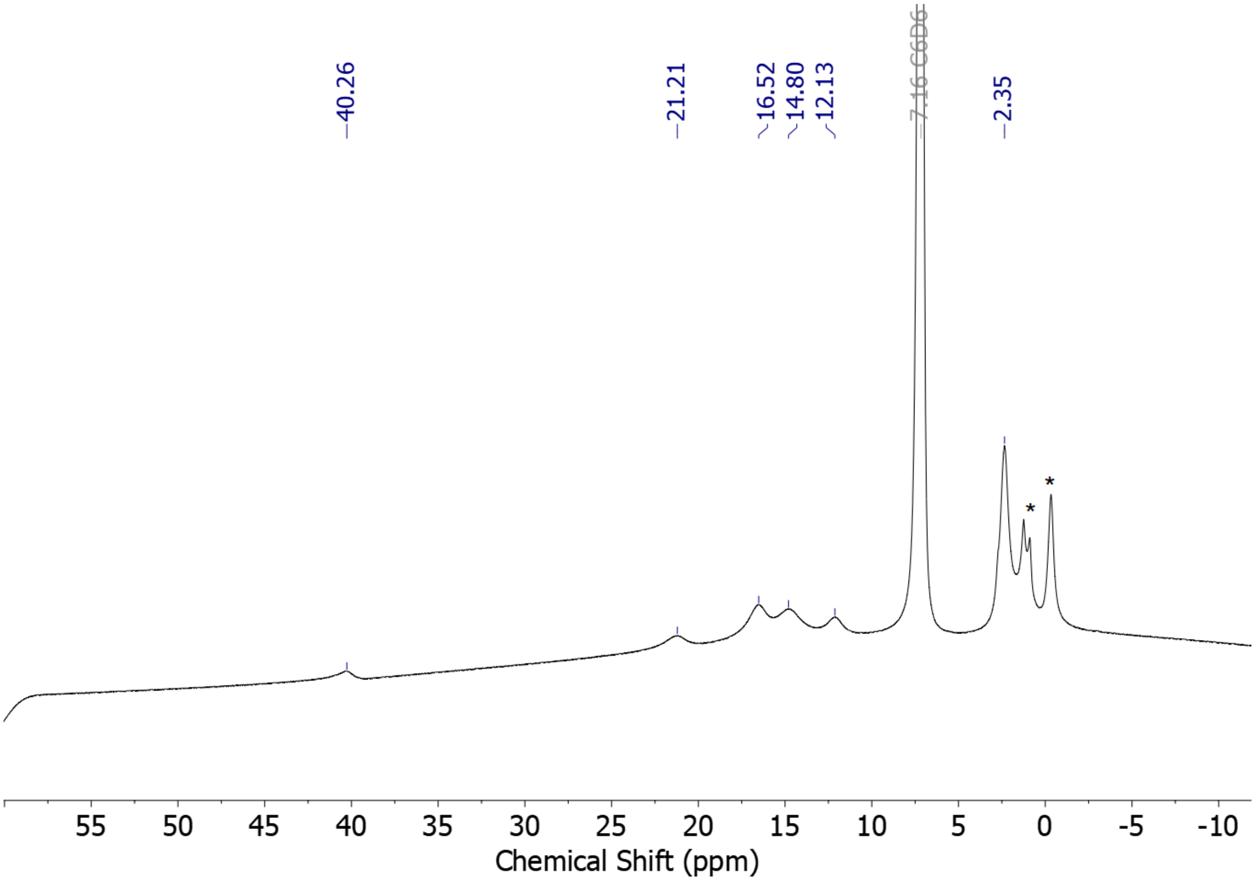

Figure S-25. ^{1}H NMR spectra for paramagnetic [(*p*-^{t}Bu-Py)Fe(^{Ph}Dbf)Cl] (**3b**). * indicates minor organic impurities remaining from the wash (hexanes).

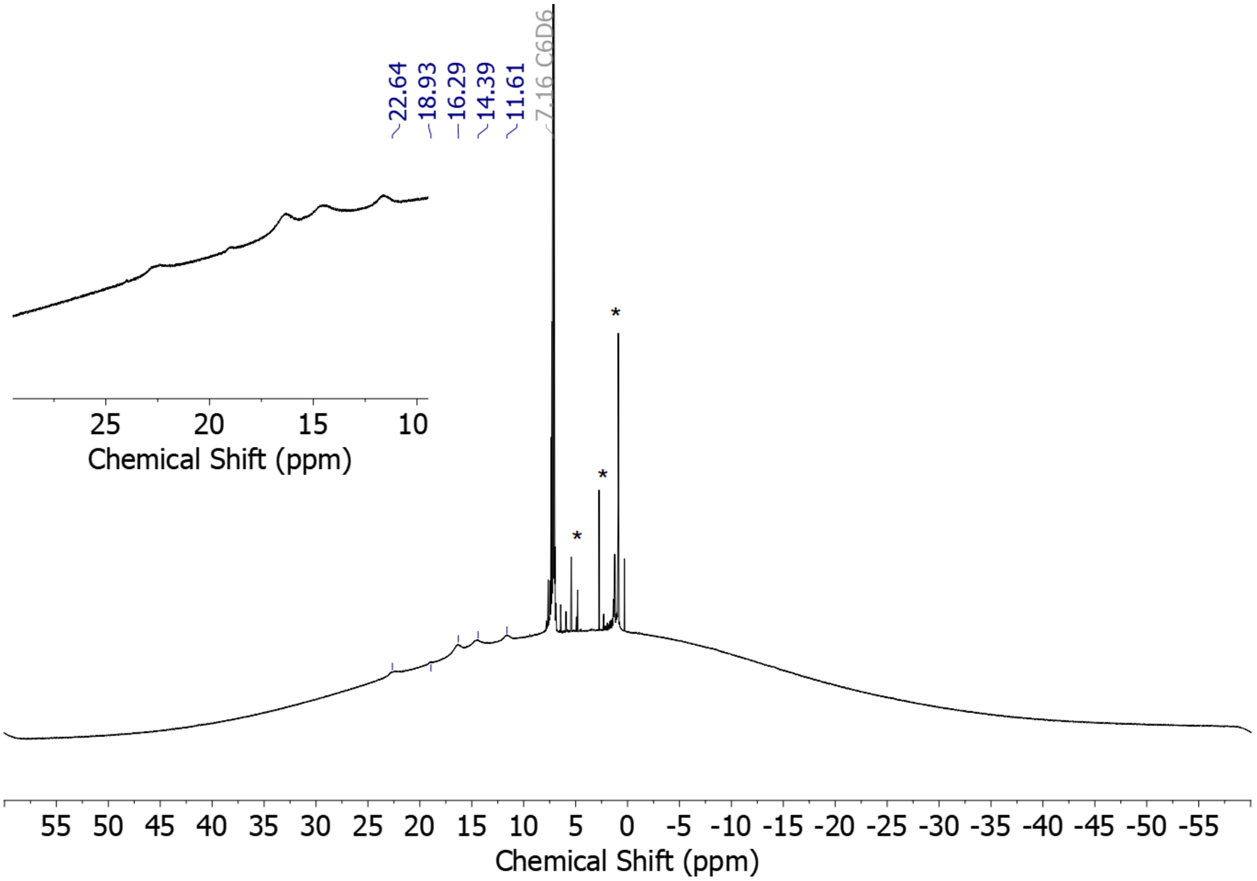

Figure S-26. ^{1}H NMR spectra for paramagnetic [(*p*-CF_3-Py)Fe(^{Ph}Dbf)Cl] (**4b**). * indicates minor organic impurities remaining from the wash (ligand, Gomberg's dimer and hexanes). Note aromatic ligand peaks near the C_6D_6 signal were not marked for clarity.

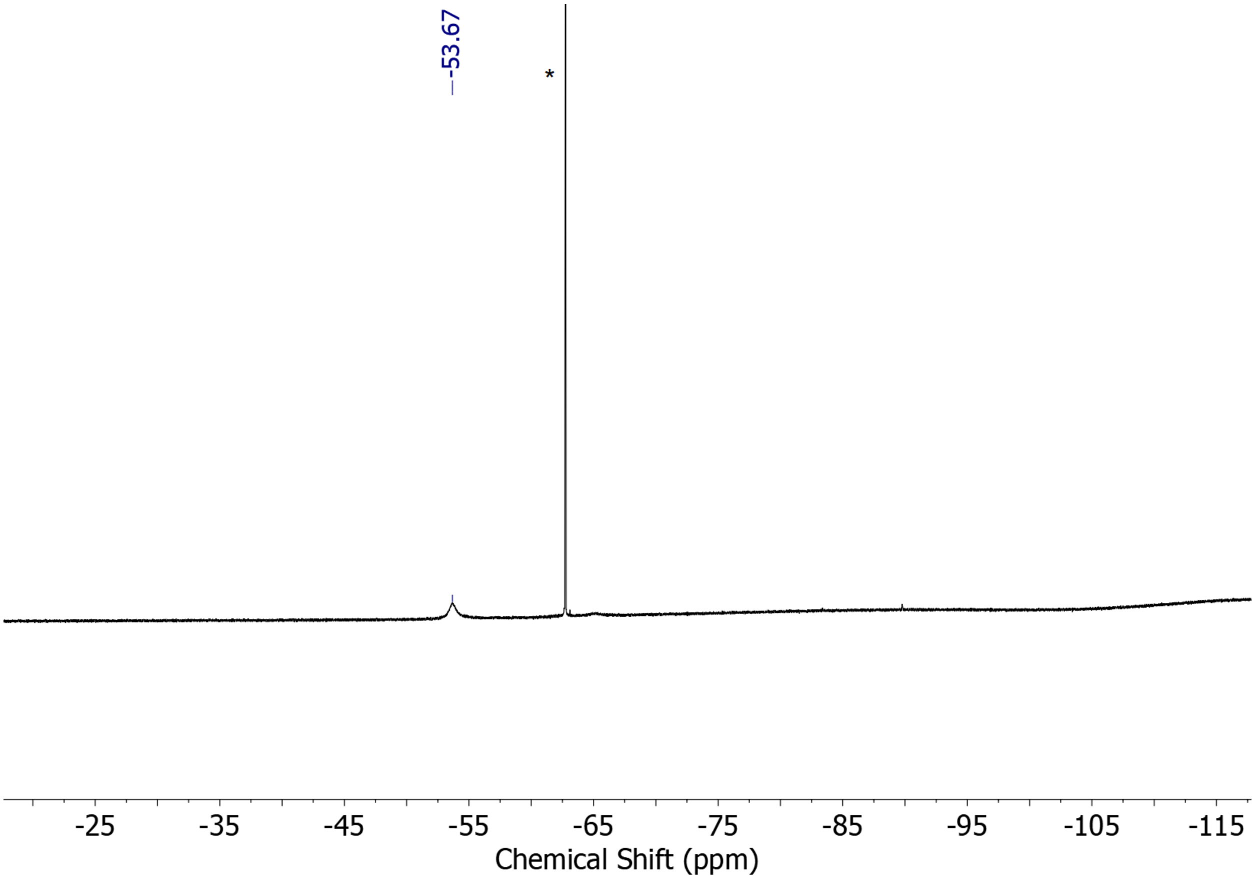

Figure S-27. ^{19}F NMR spectra for paramagnetic [(*p*-CF_3-Py)Fe(^{Ph}Dbf)Cl] (**4b**) referenced to trifluorotoluene (-62.74 ppm).

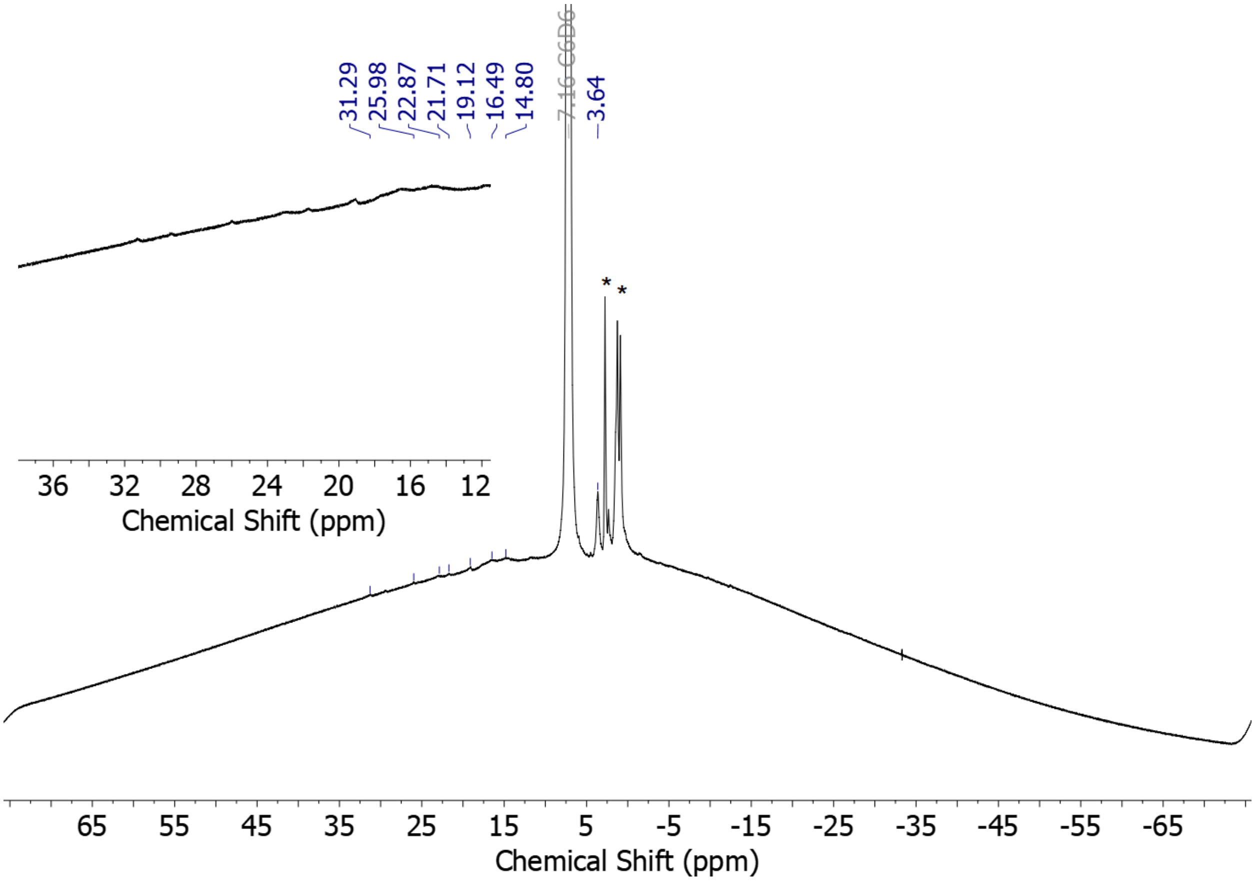

Figure S-28. ^{1}H NMR spectra for paramagnetic [(*p*-CF_3-Py)Fe_2(^{Ph}Dbf)$_2$(Cl)$_2$] (**5b**). Due to the high solubility of this complex in all solvents that led to low yields and weak NMR spectra. Inlaid spectra are to highlight the identifying paramagnetic peaks in the positive (left) regions. * indicates minor organic impurities remaining from the wash (ligand and hexanes).

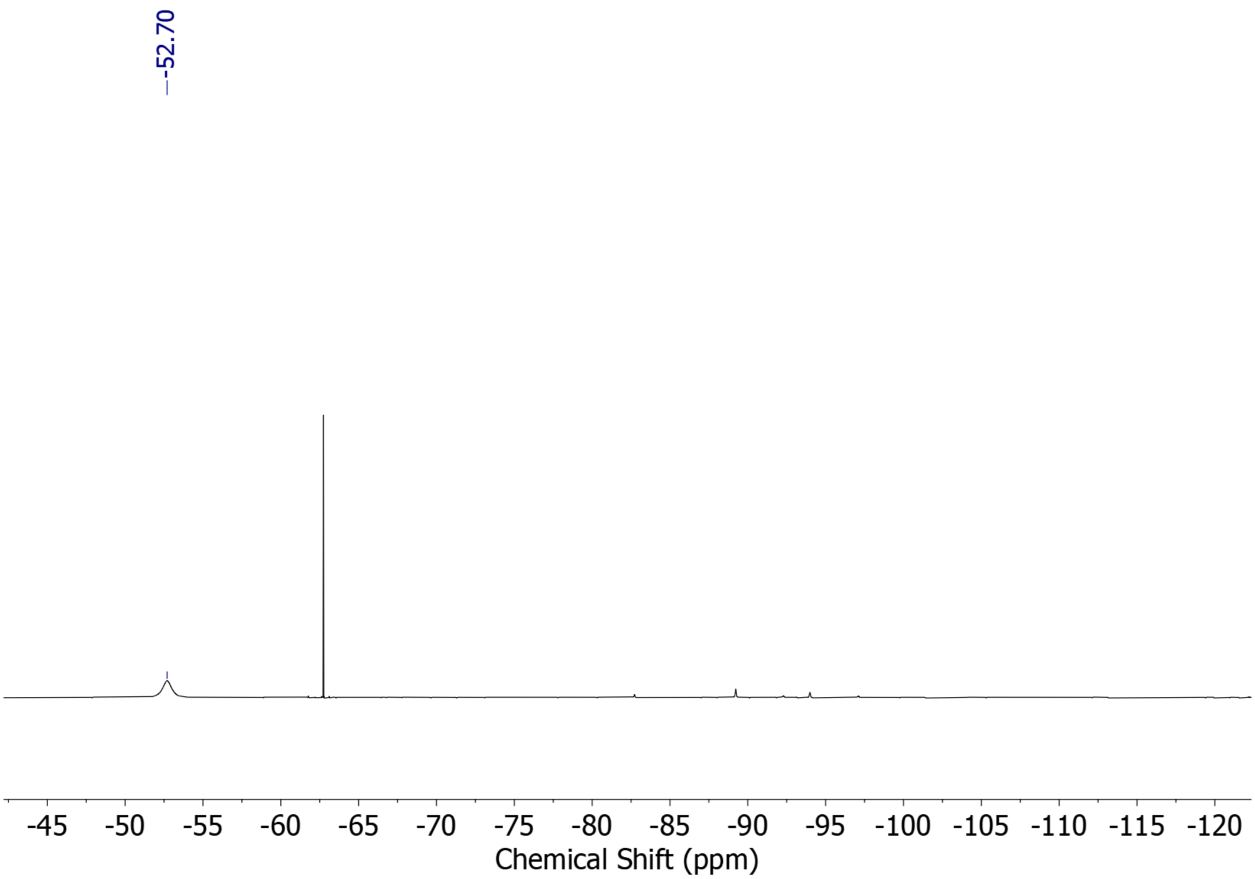

Figure S-29. ^{19}F NMR spectra for paramagnetic [(*p*-CF_3-Py)Fe_2(^{Ph}Dbf)$_2$Cl] (**5b**) referenced to trifluorotoluene (-62.74 ppm).

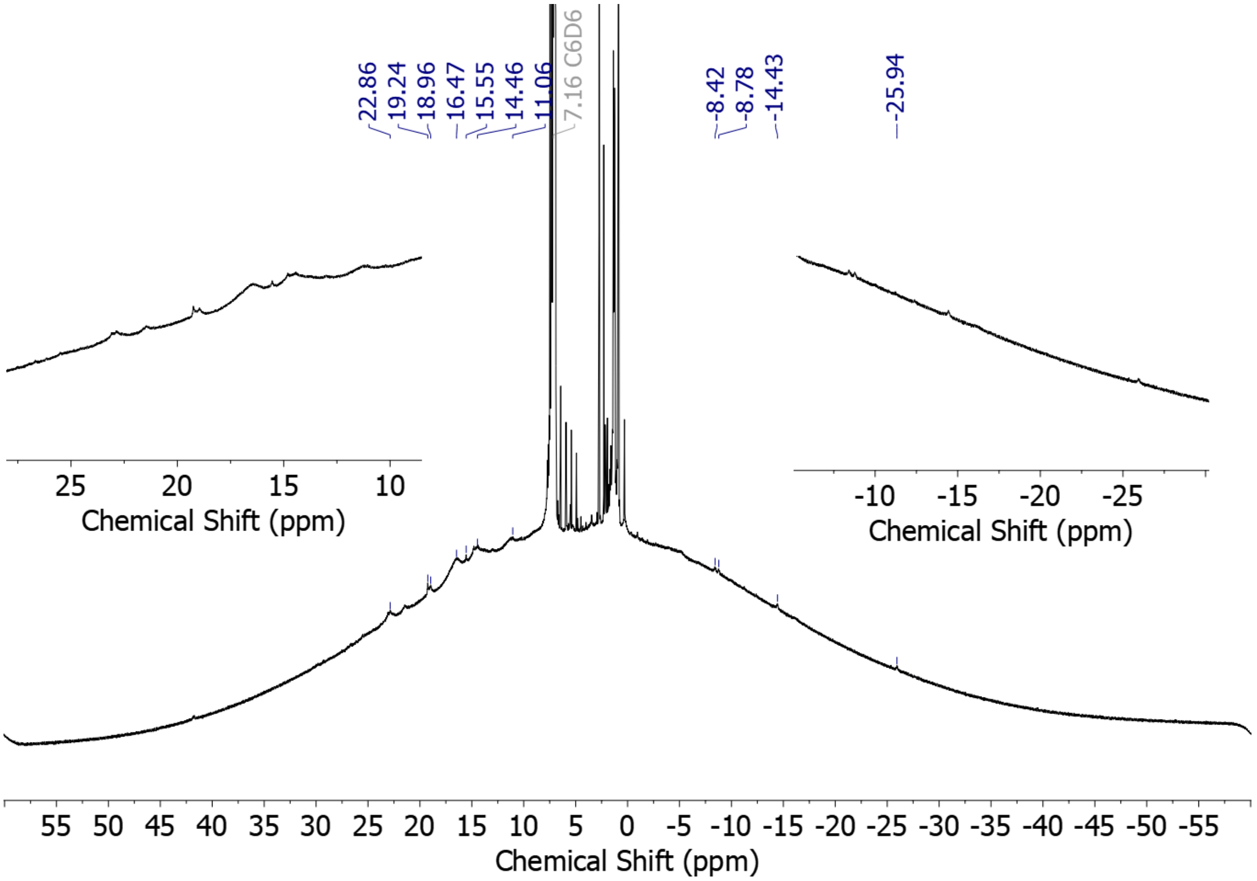

Figure S-30. ^{1}H NMR spectra for paramagnetic [*m*-CF_3-Py)Fe_2(^{Ph}Dbf)$_2$(Cl)$_2$] (**6b**) . Due to the high solubility of this complex in all solvents that led to low yields and weak NMR spectra. Inlaid spectra are to highlight the identifying paramagnetic peaks in the negative (right) and positive (left) regions. Organic impurities remaining from the wash are the unlabeled peaks in the diamagnetic region (ligand, Gomberg's dimer and hexanes). Note aromatic ligand peaks near the C_6D_6 signal were not marked for clarity.

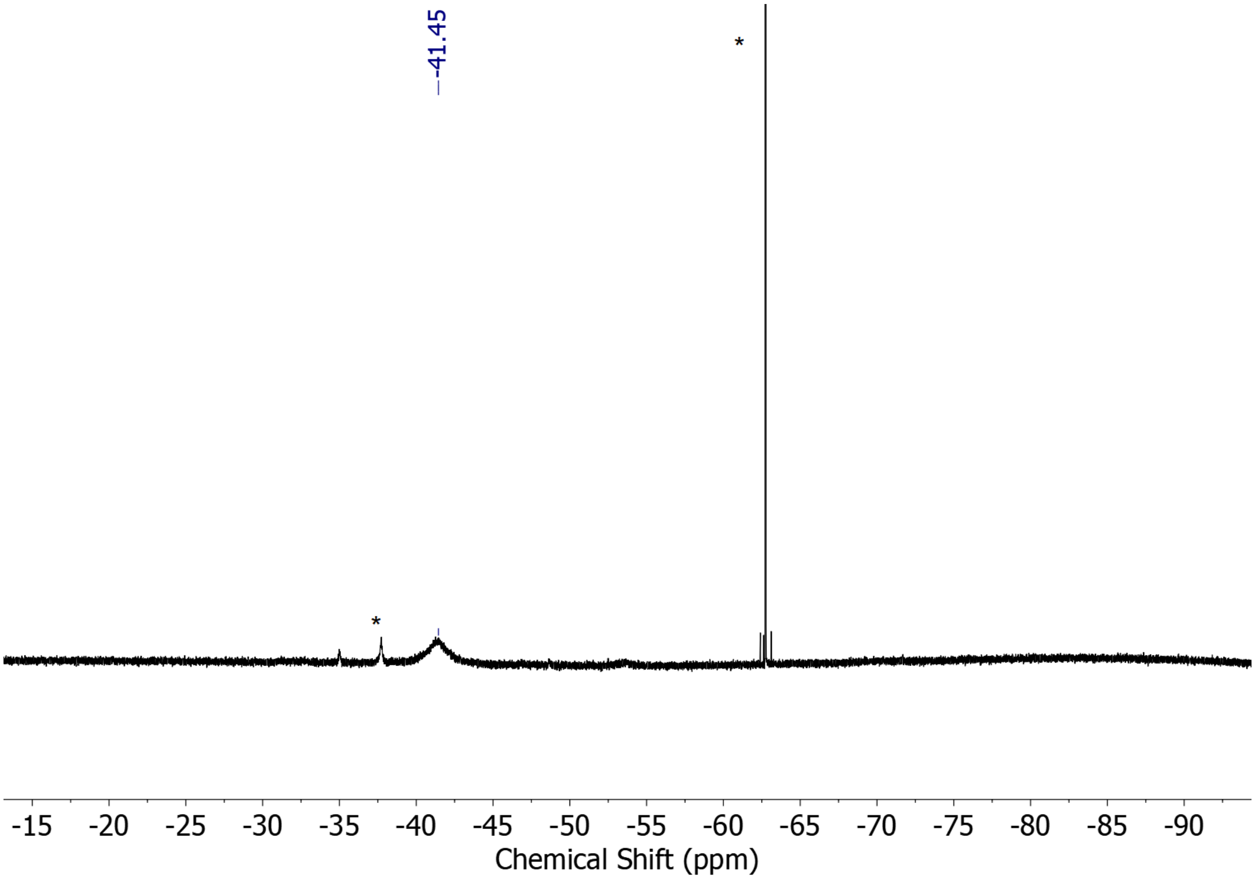

Figure S-31. ^{19}F NMR spectra for paramagnetic [(m-CF_3-Py)Fe_2(^{Ph}Dbf)$_2$(Cl)$_2$] (**6b**) referenced to trifluorotoluene (-63.72 ppm). * indicates the standard (*larger* peak) and minor organic impurity (*smaller peak*) in the ^{19}F standard capillary.

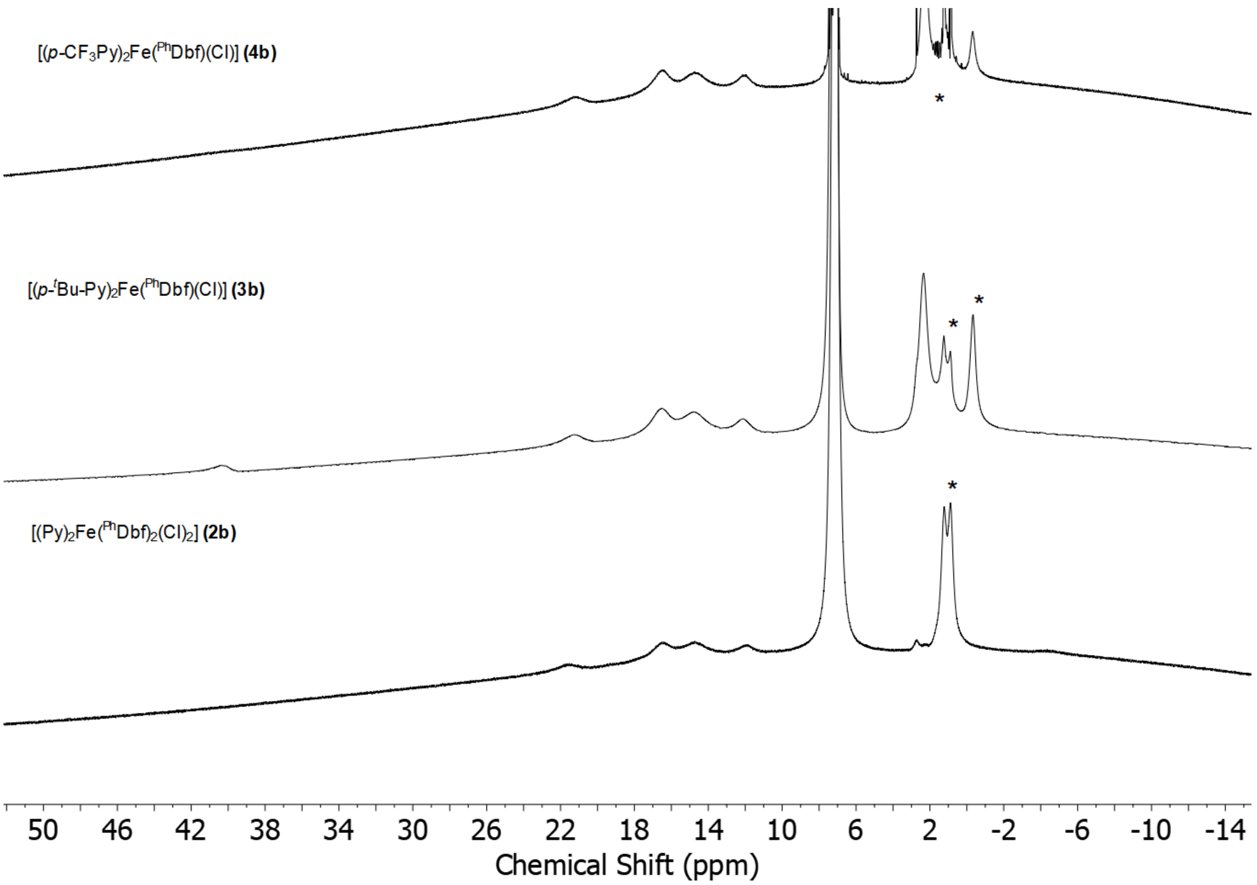

Figure S-32. Stacked ^{1}H NMR spectra for paramagnetic [(Py)Fe(^{Ph}Dbf)Cl] (**2b**), [(*p*-^{t}Bu-Py)Fe(^{Ph}Dbf)Cl] (**3b**), and [(*p*-CF3$_3$-Py)Fe(^{Ph}Dbf)Cl] (**4b**). * indicates minor organic impurities remaining from the wash (hexanes) and free ligand. Note aromatic ligand peaks near the C_6D_6 signal were not marked for clarity.

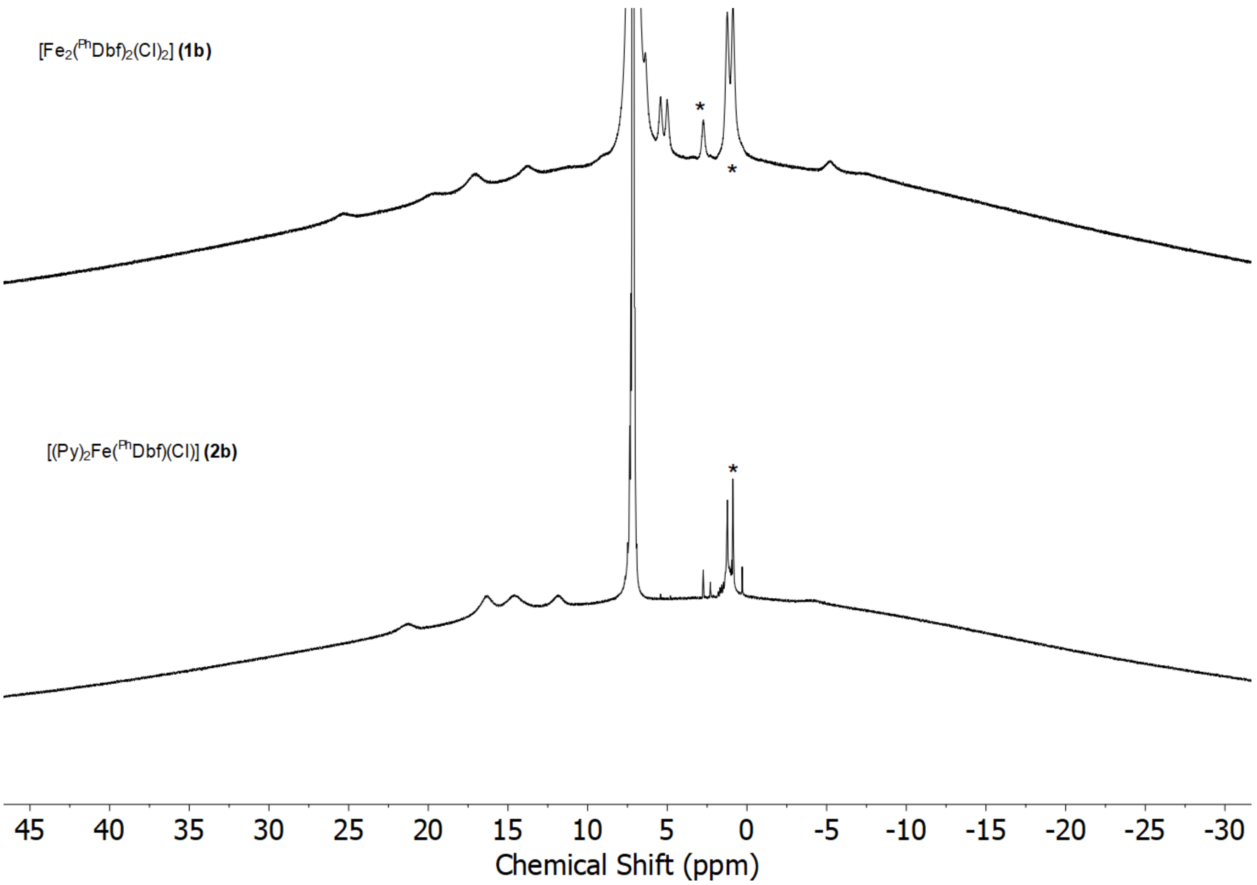

Figure S-33. Stacked ^{1}H NMR spectra for paramagnetic [(Py)Fe(^{Ph}Dbf)Cl] (**2b**) and [Fe_2(^{Ph}Dbf)$_2$$Cl_2$] (**1b**). * indicates minor organic impurities remaining from the wash (hexanes) and free ligand. Note aromatic ligand peaks near the C_6D_6 signal were not marked for clarity.

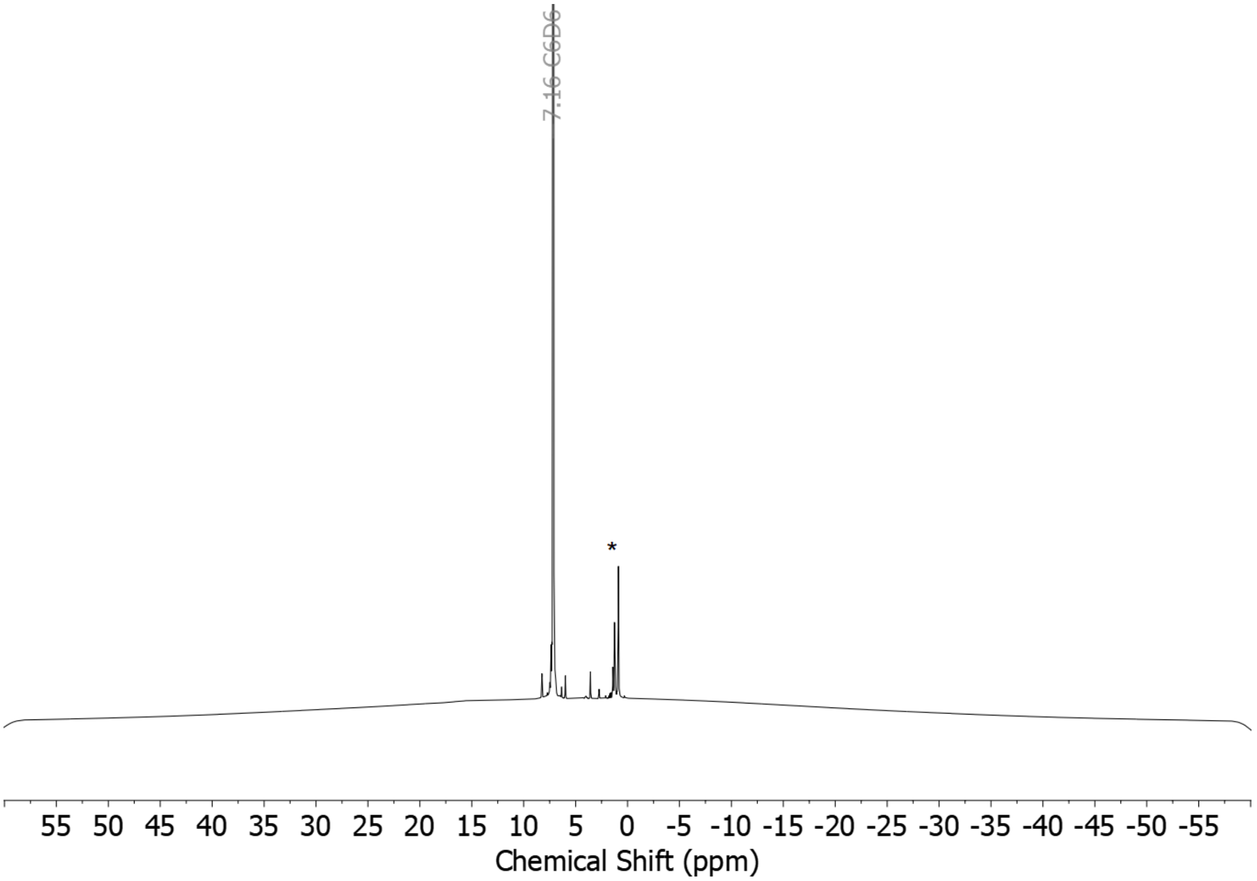

Figure S-34. ^{1}H NMR spectra for paramagnetic [$Py_2Fe(^{Ph}Dbf)$][PF_6] (**2c**). * indicates minor organic impurities remaining from the wash (hexanes) and free ligand. Note aromatic ligand peaks near the C_6D_6 signal were not marked for clarity.

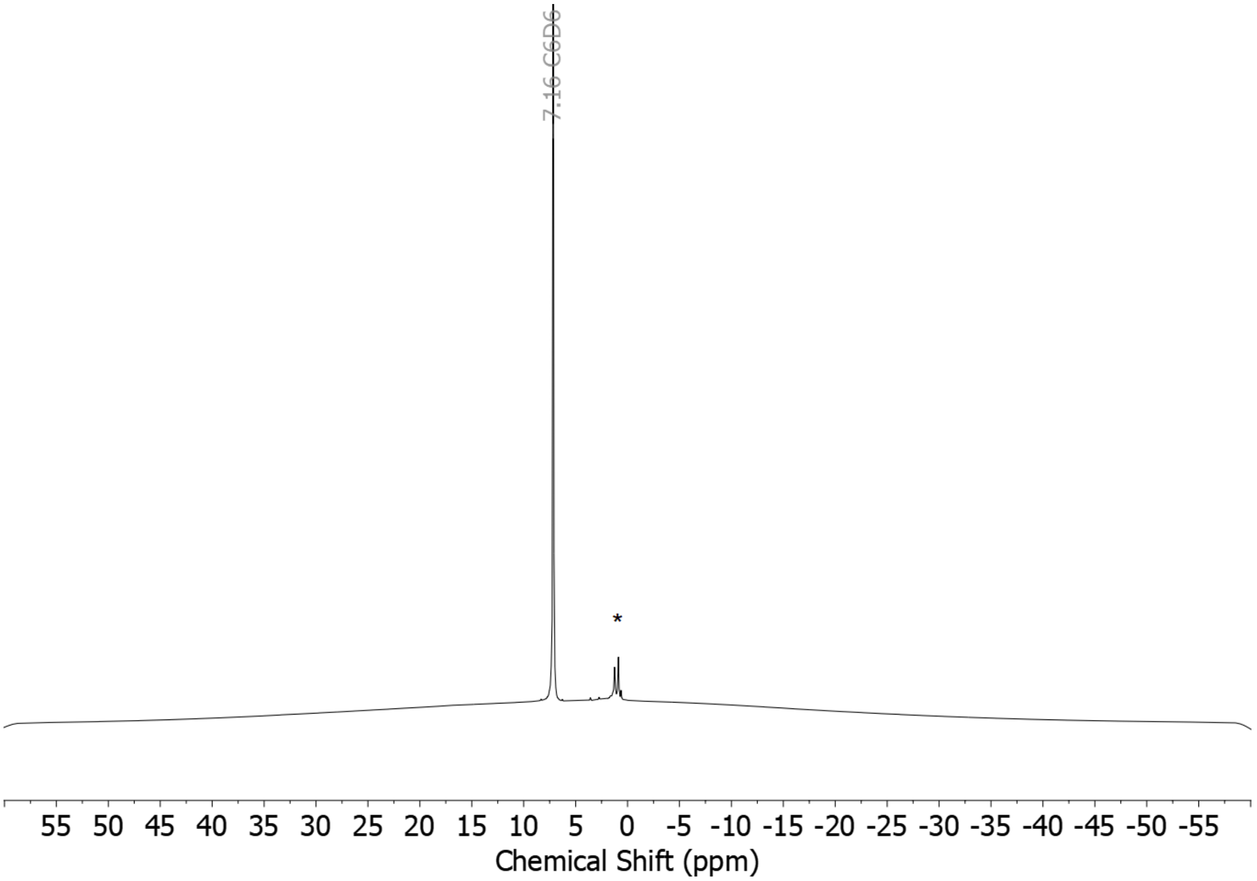

Figure S-35. ^{1}H NMR spectra for paramagnetic [(p-^{t}Bu-Py)$_2$Fe(^{Ph}Dbf)][PF_6] (**3c**). * indicates minor organic impurities remaining from the wash (hexanes) and free ligand. Note aromatic ligand peaks near the C_6D_6 signal were not marked for clarity.

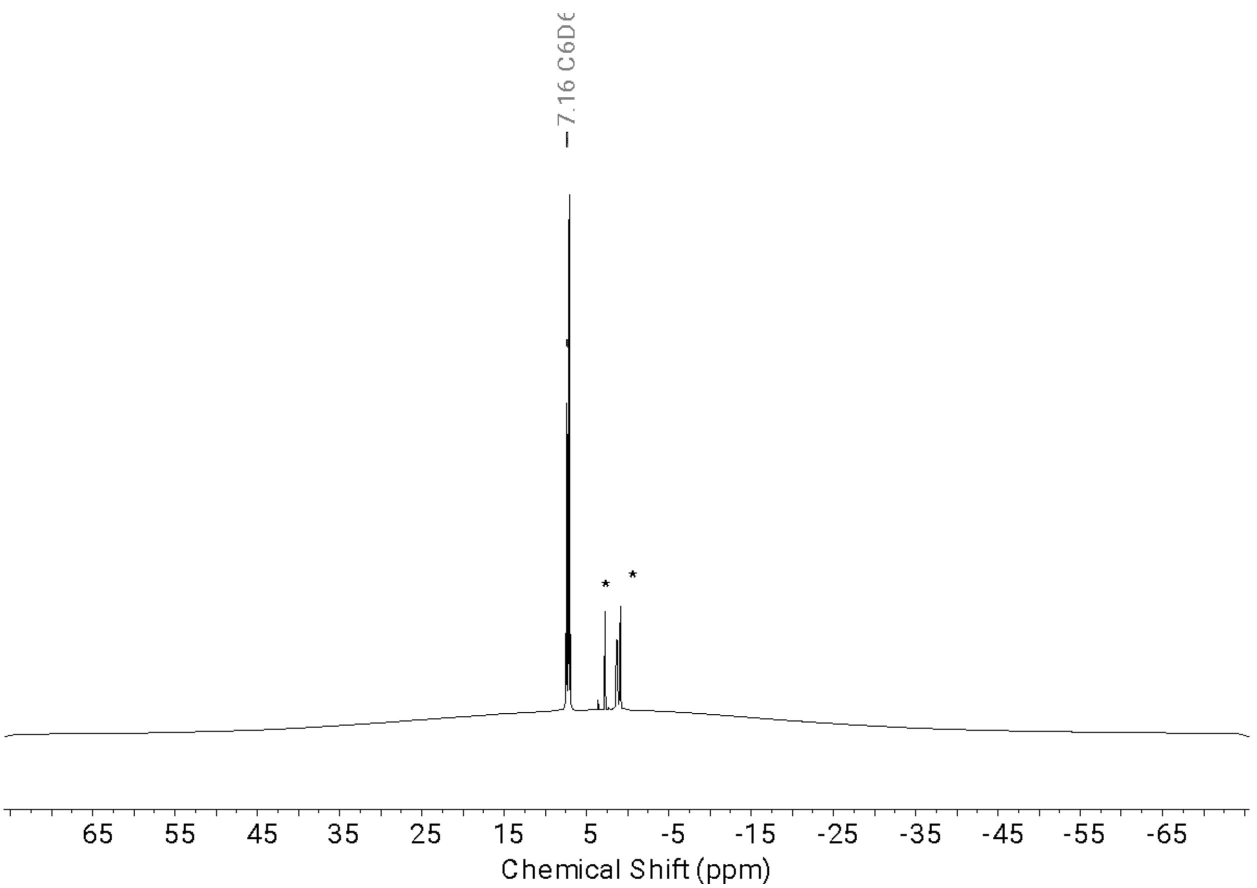

Figure S-36. ^{1}H NMR spectra for paramagnetic [(*p*-CF_3-Py)$_2$Fe(^{Ph}Dbf)][PF_6] (**4c**). * indicates minor organic impurities remaining from the wash (hexanes) and free ligand. Note aromatic ligand peaks near the C_6D_6 signal were not marked for clarity.

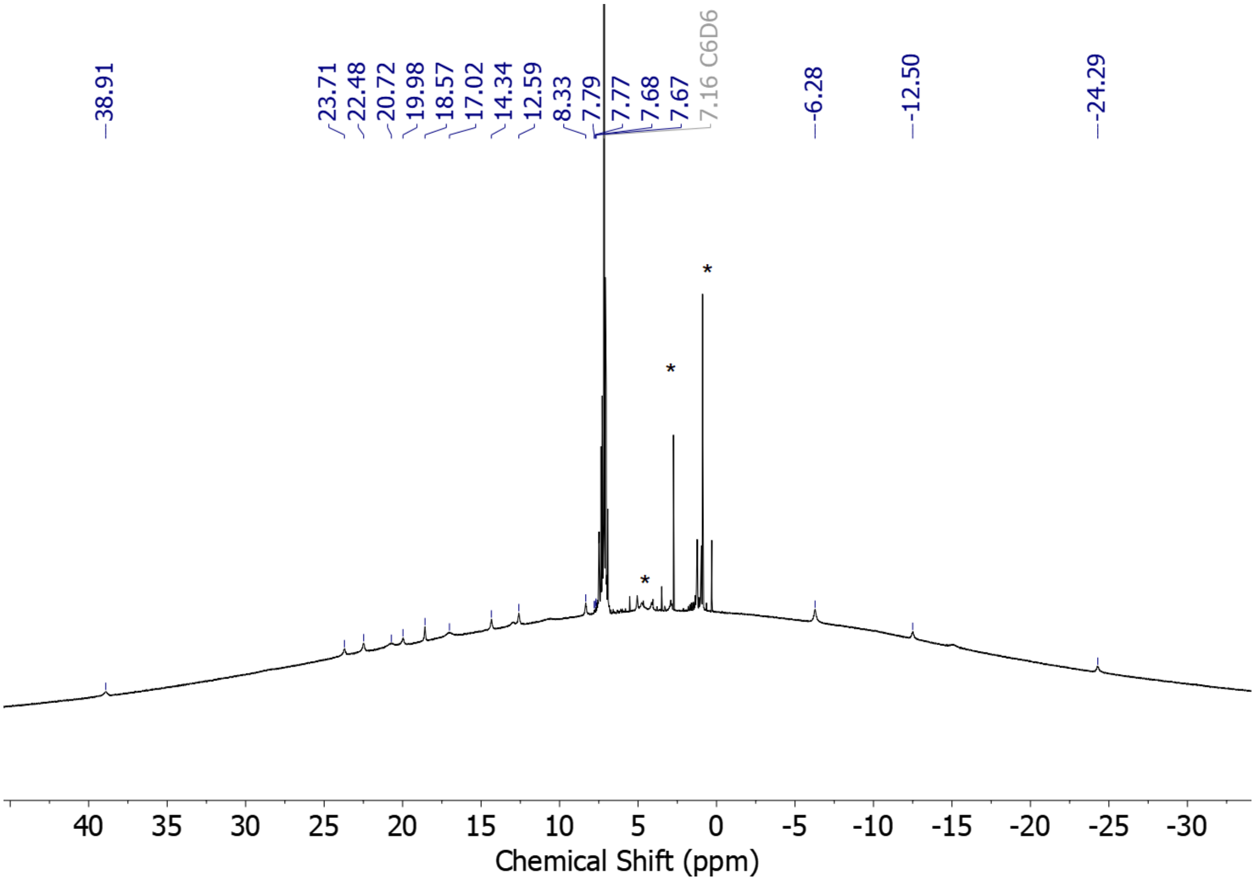

Figure S-37. ^{1}H NMR spectra for the reaction of $[Fe_2(^{Ph}Dbf)_2]$ (**1a**) with $FcPF_6$. * indicates minor organic impurities remaining from the wash (hexanes) and free ligand. Note aromatic ligand peaks near the C_6D_6 signal were not marked for clarity.

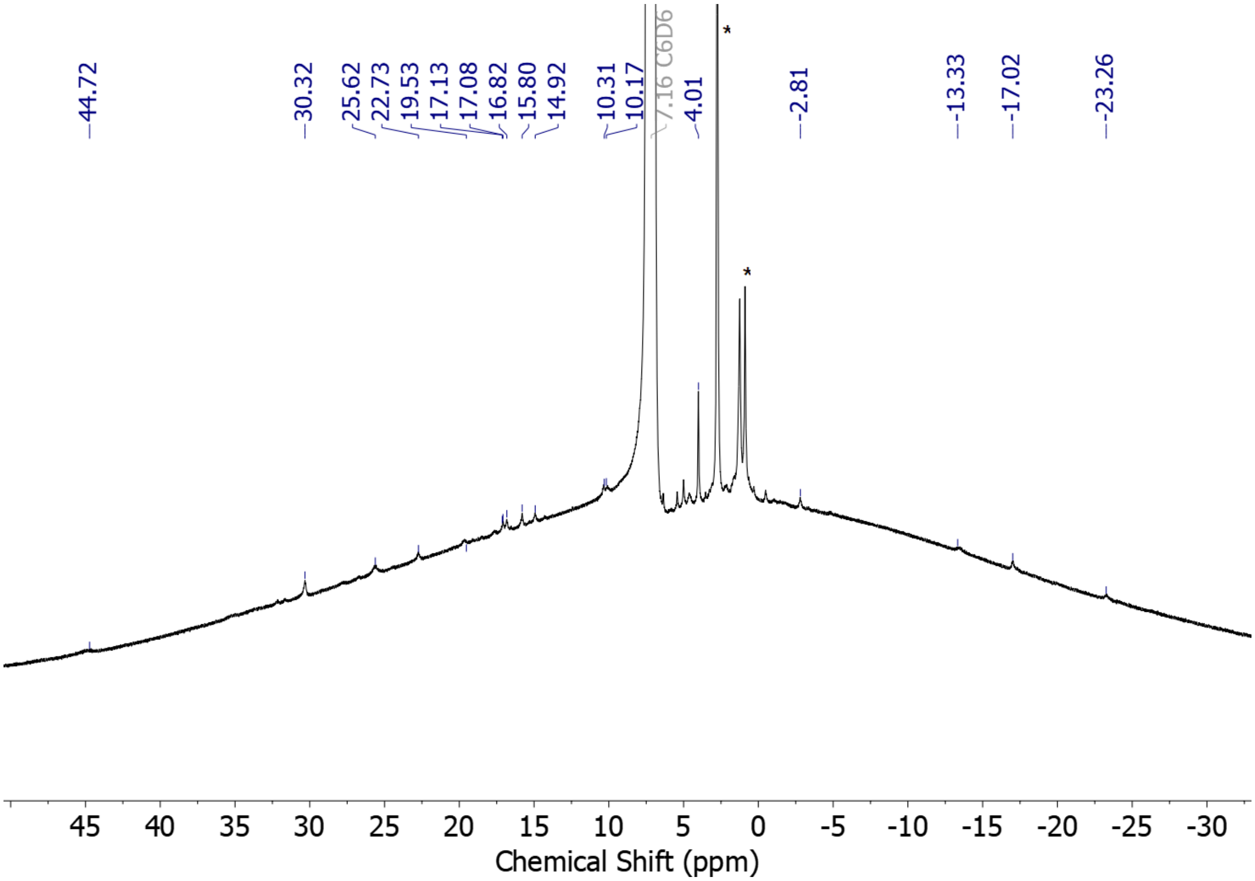

Figure S-38. ^{1}H NMR spectra for the reaction of [(*p*-CF_3-Py)Fe_2(^{Ph}Dbf)$_2$] (**5a**) with $FcPF_6$. * indicates minor organic impurities remaining from the wash (hexanes) and free ligand. Note aromatic ligand peaks near the C_6D_6 signal were not marked for clarity.

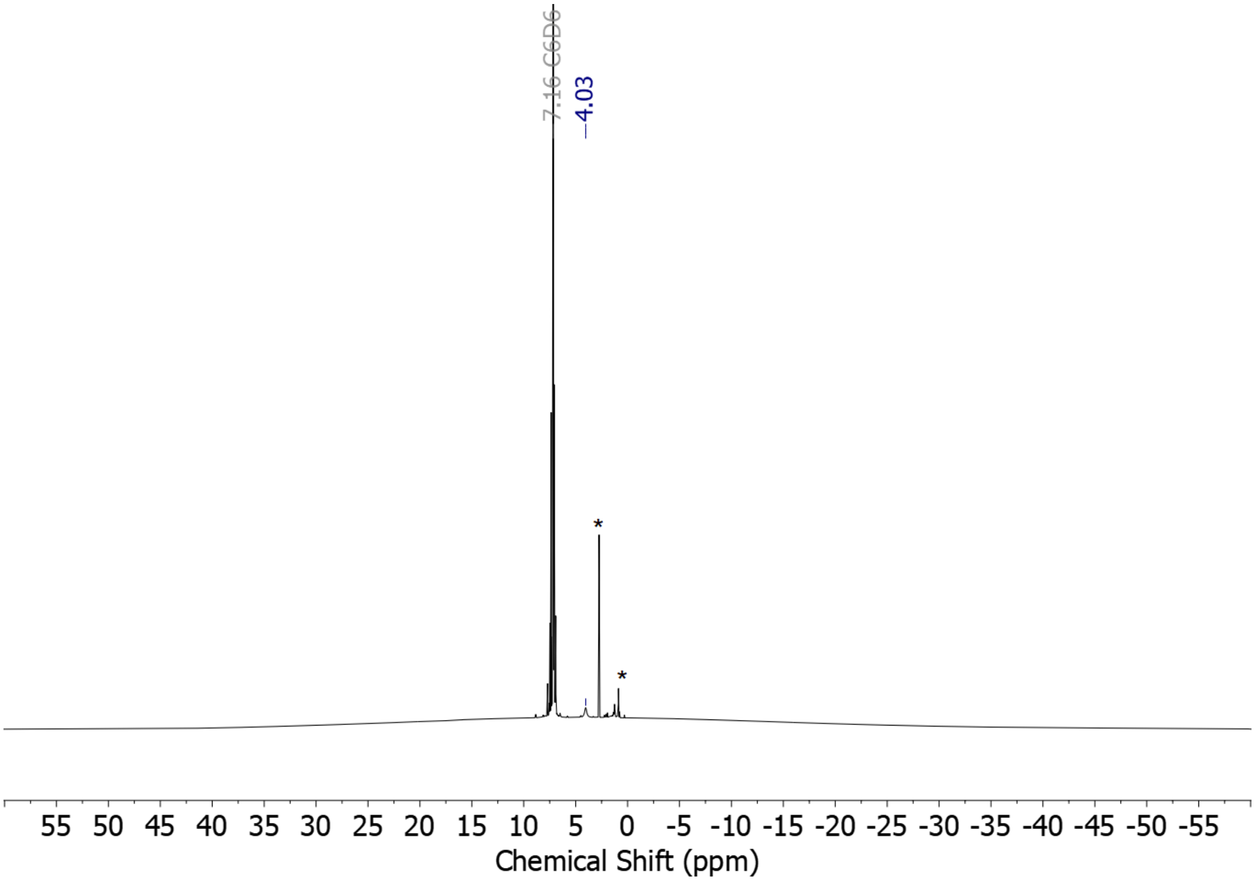

Figure S-39. ^{1}H NMR spectra for the reaction of [(*m*-CF_3-Py)Fe_2(^{Ph}Dbf)$_2$] (**6a**) with $FcPF_6$. * indicates minor organic impurities remaining from the wash (hexanes) and free ligand.

Electron Paramagnetic Resonance (EPR)

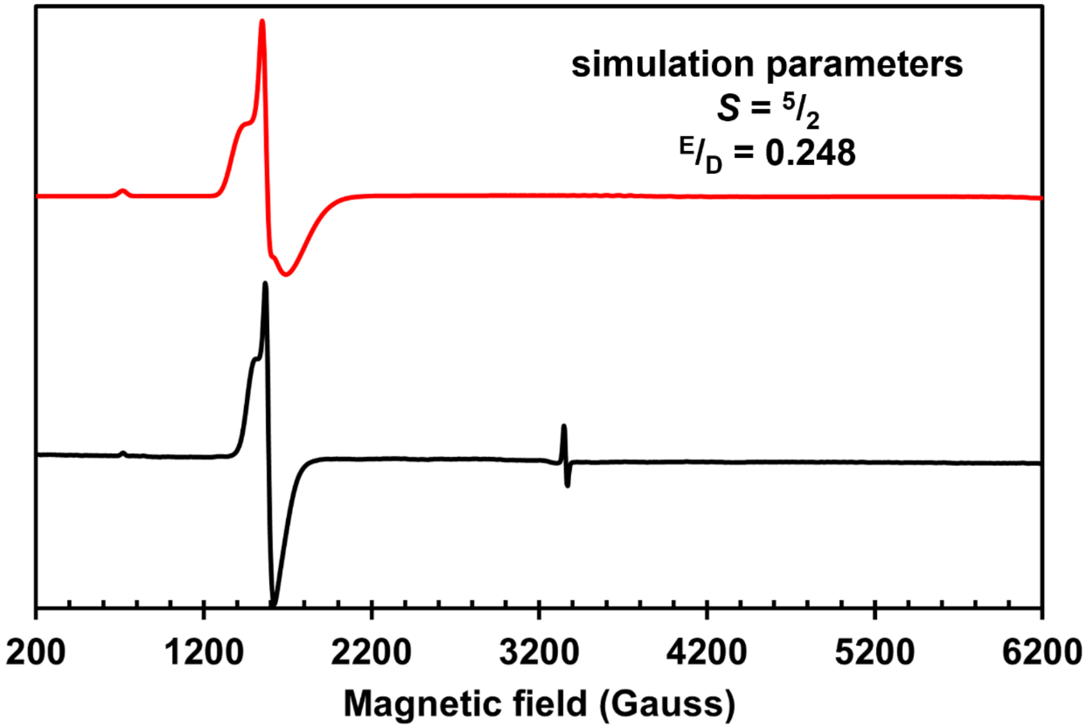

Figure S-40. Frozen toluene EPR spectrum of $[Fe_2(^{Ph}Dbf)_2(Cl)_2]$ (**1b**) at 80 K (black). The red line represents a simulation with VisualRhombo. The small signal at 3359 G corresponds to left-over Gomberg's dimer generated during the reaction.

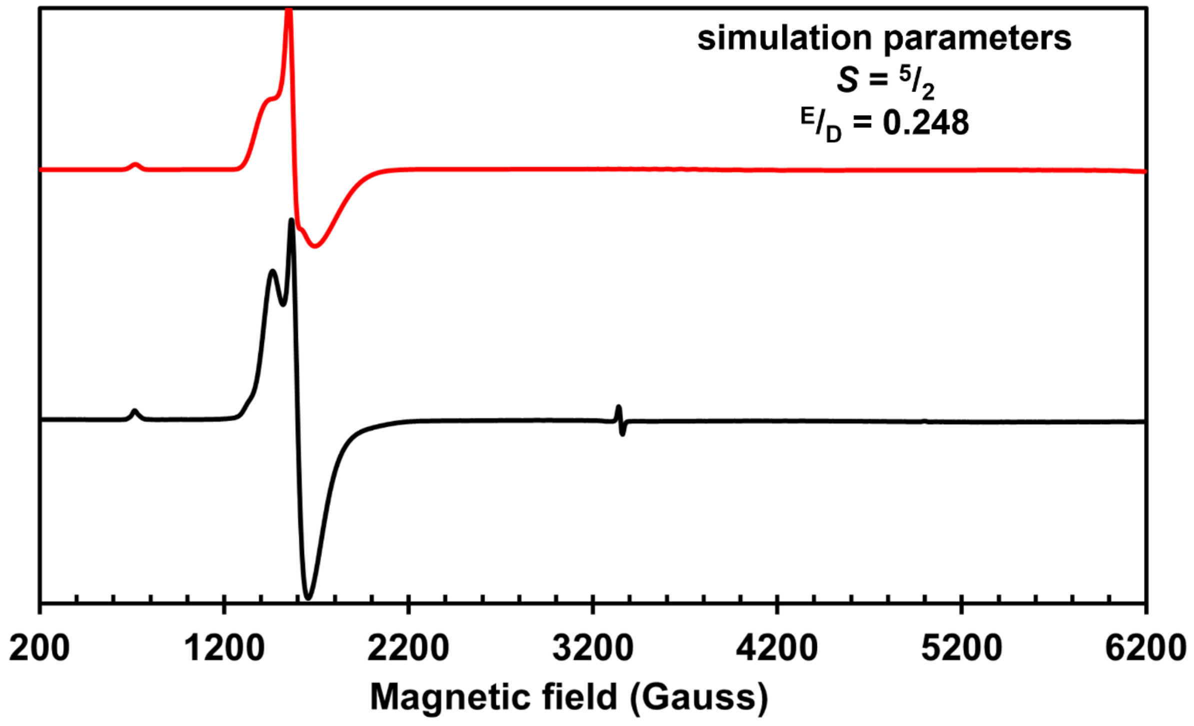

Figure S-41. Frozen toluene EPR spectrum of [PyFe(^{Ph}Dbf)Cl] (**2b**) at 80 K (black). The red line represents a simulation with VisualRhombo.

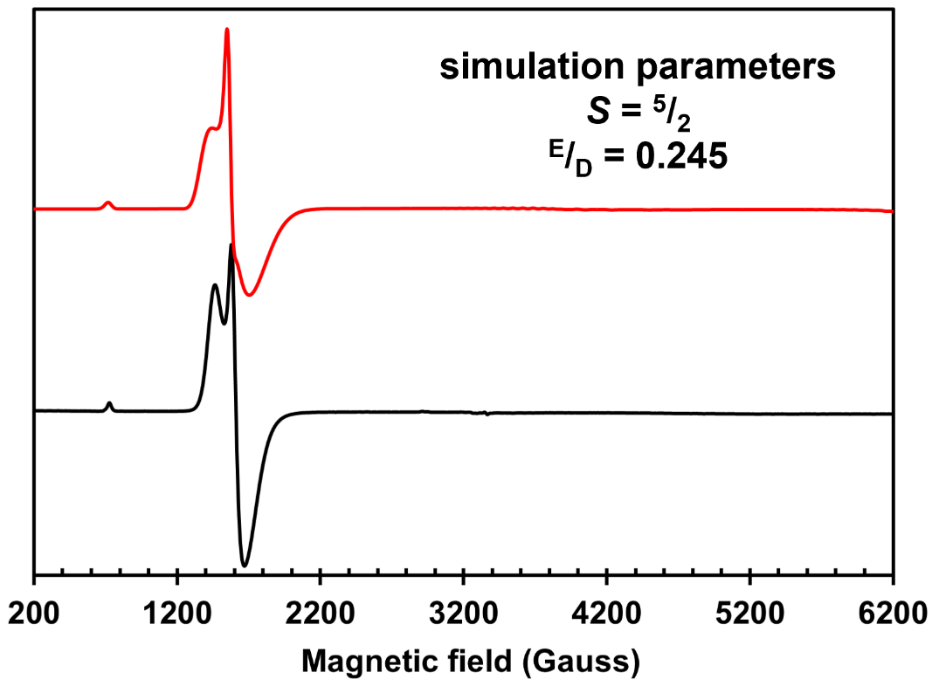

Figure S-42. Frozen toluene EPR spectrum of [(*p*-^{t}Bu-Py)Fe(^{Ph}Dbf)Cl] (**3b**) at 80 K (black). The red line represents a simulation with VisualRhombo.

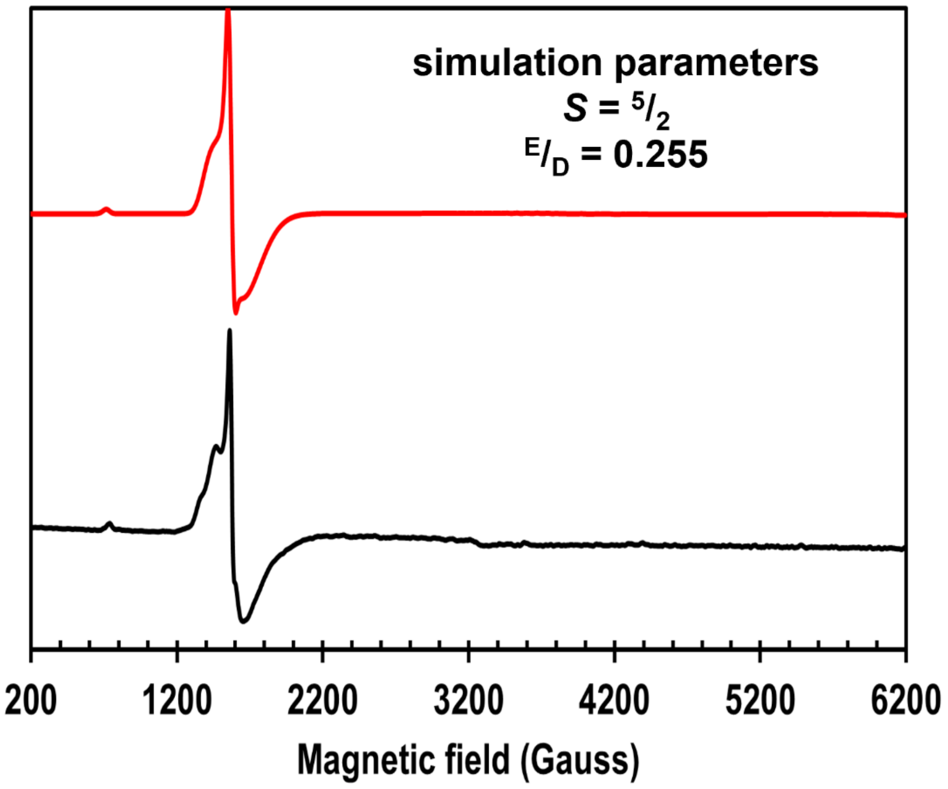

Figure S-43. Frozen toluene EPR spectrum of [(*p*-CF_3-Py)Fe(^{Ph}Dbf)Cl] (**4b**) at 80 K (black). The red line represents a simulation with VisualRhombo.

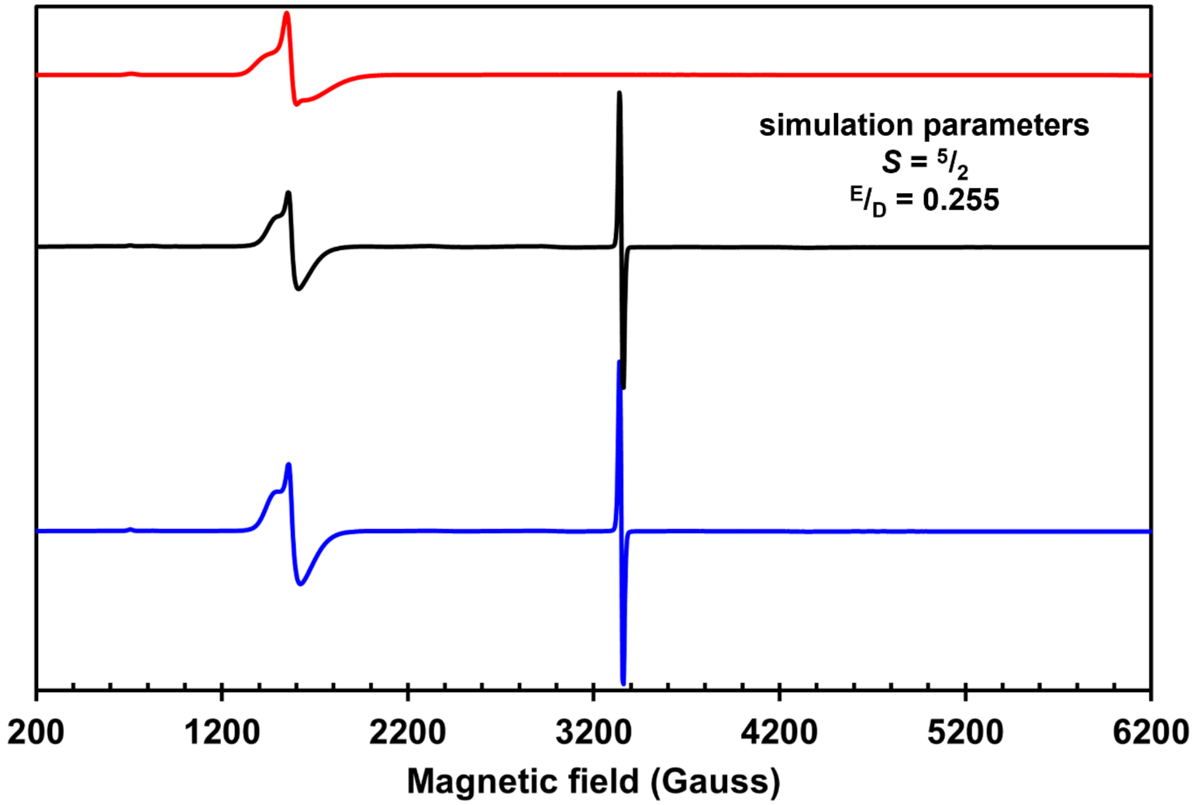

Figure S-44. Frozen toluene EPR spectrum of [(*p*-CF_3-Py)Fe_2^{Ph}Dbf)$_2$(Cl)$_2$] (**5b**) at 80 K (black). The red line represents a simulation with VisualRhombo and the blue line represents the spectrum of **5b** prior to washing, illustrating that the organic radical is not removed. This suggests either that some decomposition is occurring during the reaction (the sample could be unstable) or due to very similar solubilities between **5b** and the Gomberg's dimer byproduct, the two cannot be separated.

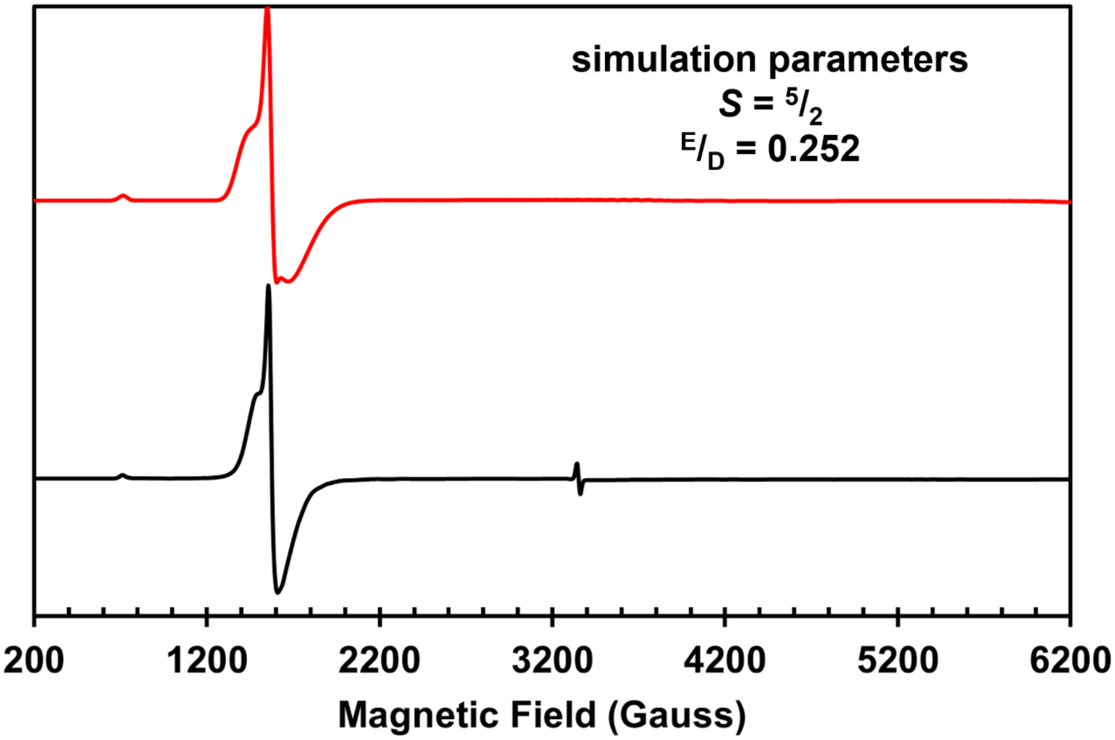

Figure S-45. Frozen toluene EPR spectrum of [(*m*-CF_3-Py)Fe_2(^{Ph}Dbf)$_2$(Cl)$_2$] (**6b**) at 80 K (black). The red line represents a simulation with VisualRhombo. The small signal at 3359 G corresponds to left-over Gomberg's dimer generated during the reaction.

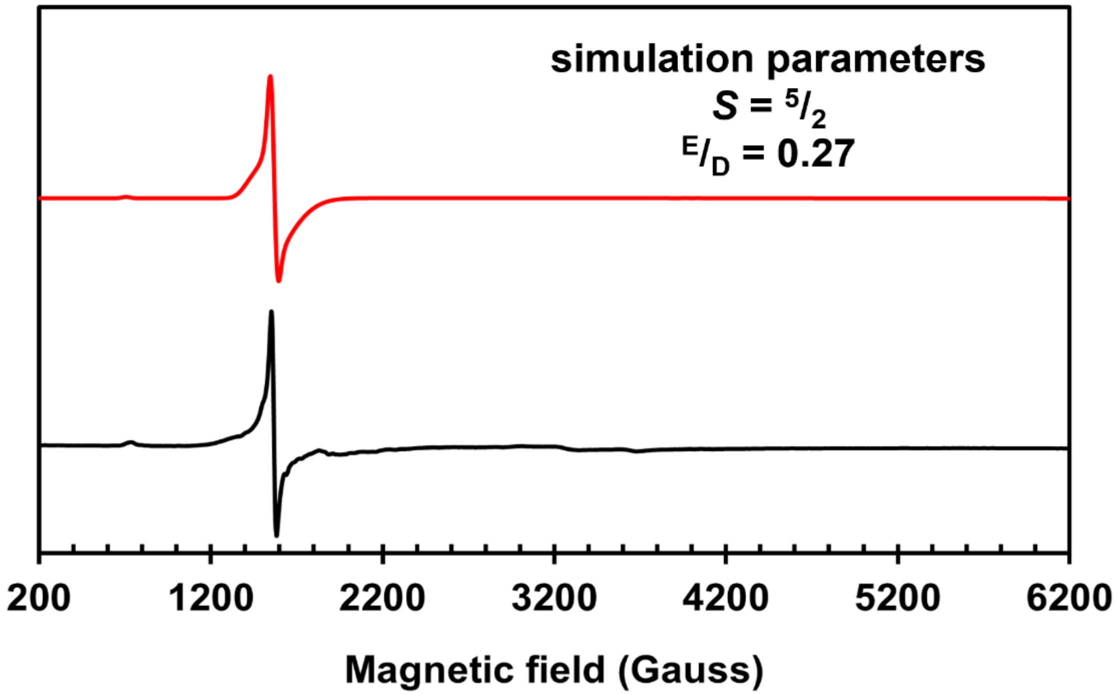

Figure S-46. Frozen toluene EPR spectrum of $[Py_2Fe(^{Ph}Dbf)][PF_6]$ (**2c**) at 80 K (black). The red line represents a simulation with VisualRhombo.

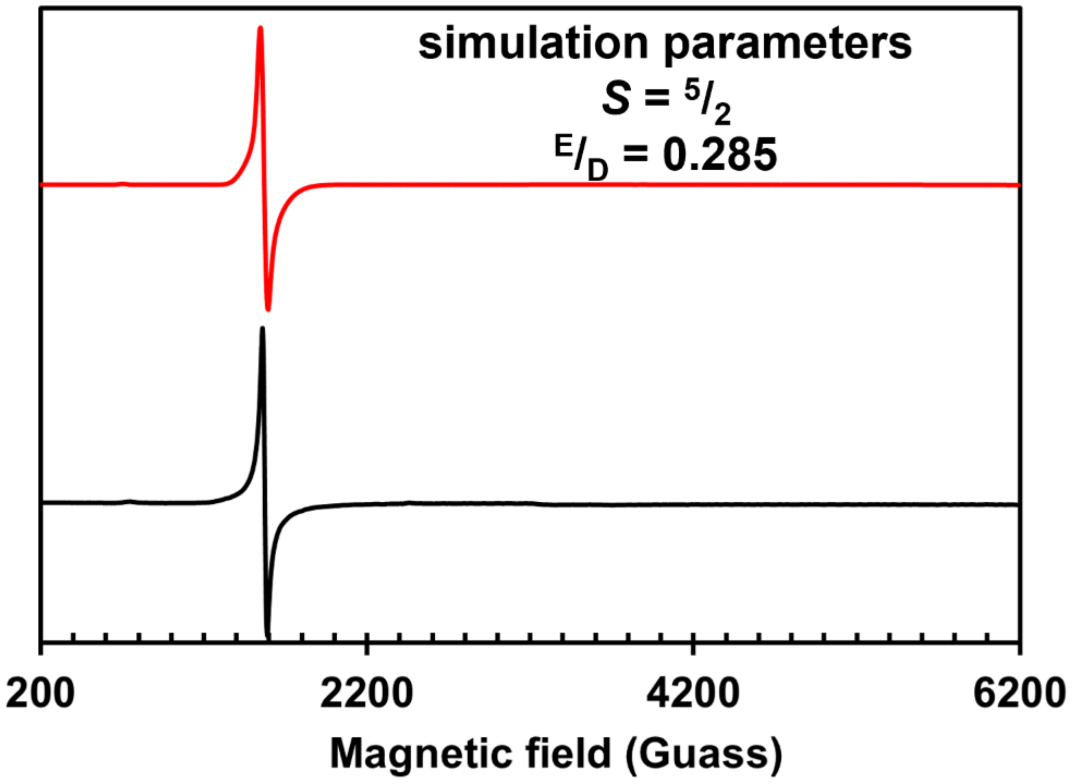

Figure S-47. Frozen toluene EPR spectrum of [(*p*-^{t}Bu-Py)$_2$Fe(^{Ph}Dbf)][PF_6] (**3c**) at 80 K (black). The red line represents a simulation with VisualRhombo.

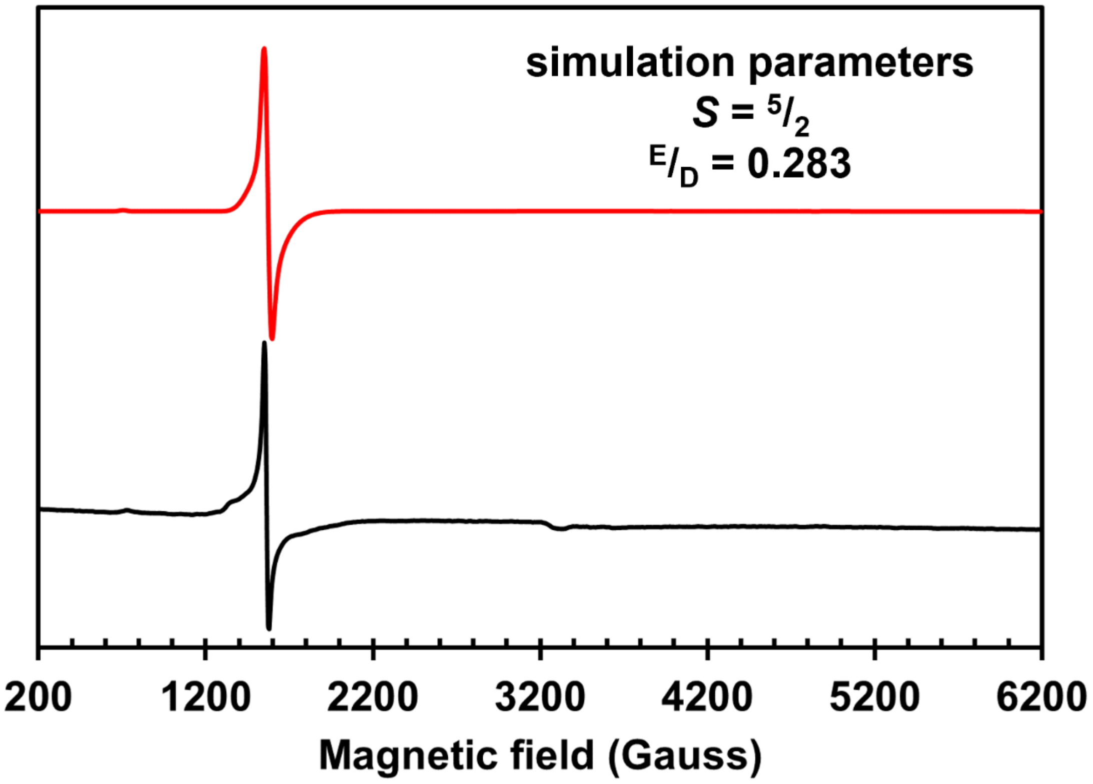

Figure S-48. Frozen toluene EPR spectrum of [(*p*-CF_3-Py)$_2$Fe(^{Ph}Dbf)][PF_6] (**4c**) at 80 K (black). The red line represents a simulation with VisualRhombo.

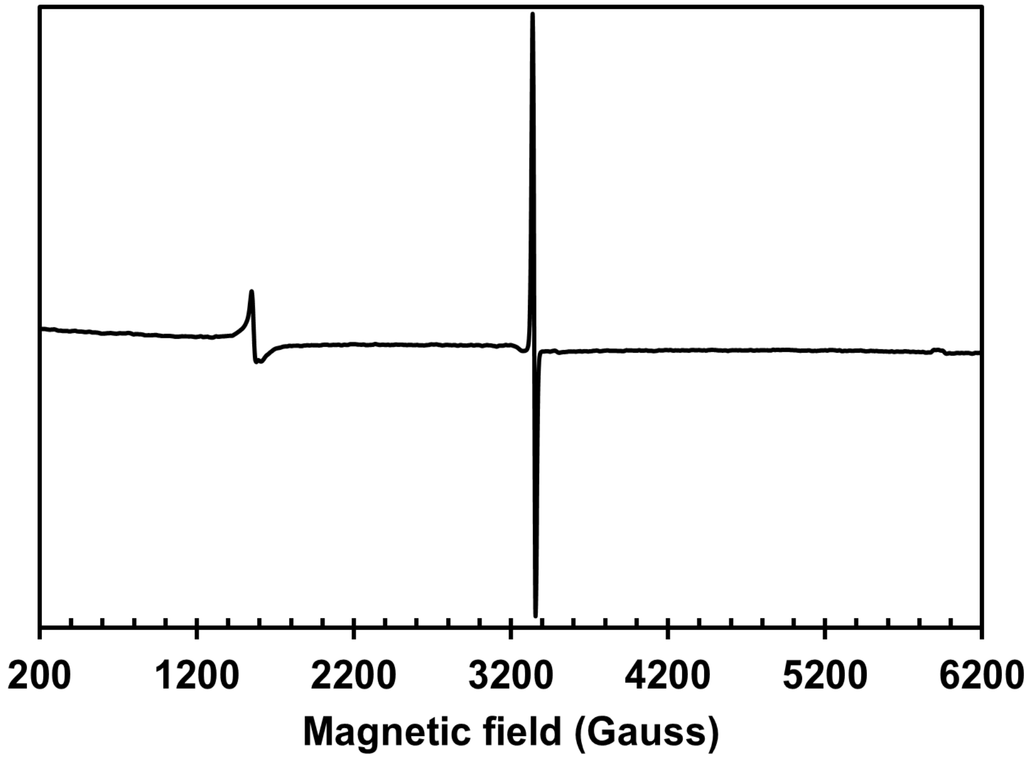

Figure S-49. Frozen toluene EPR spectrum of the reaction of $[Fe_2(^{Ph}Dbf)_2]$ (**1a**) with 2 equiv. $FcPF_6$ at 80 K.

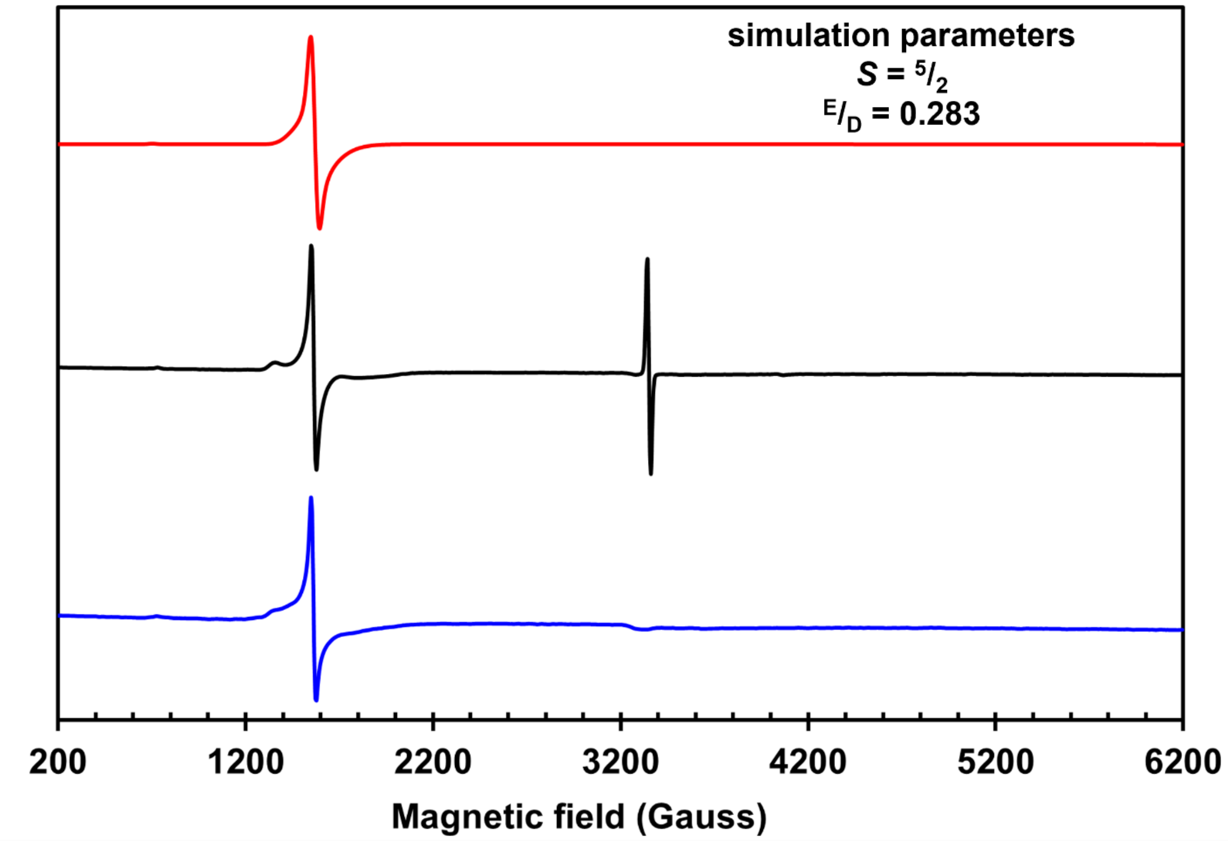

Figure S-50. Frozen toluene EPR spectrum of the reaction of [(*p*-CF_3-Py)Fe_2(^{Ph}Dbf)$_2$] (**5a**) with 2 equiv. $FcPF_6$ at 80 K (black). The red line represents a simulation with VisualRhombo and the blue line corresponds to the frozen toluene EPR spectrum of [(*p*-CF_3-Py)$_2$$Fe_2$(^{Ph}Dbf)][$PF_6$] (**4c**) at 80 K.

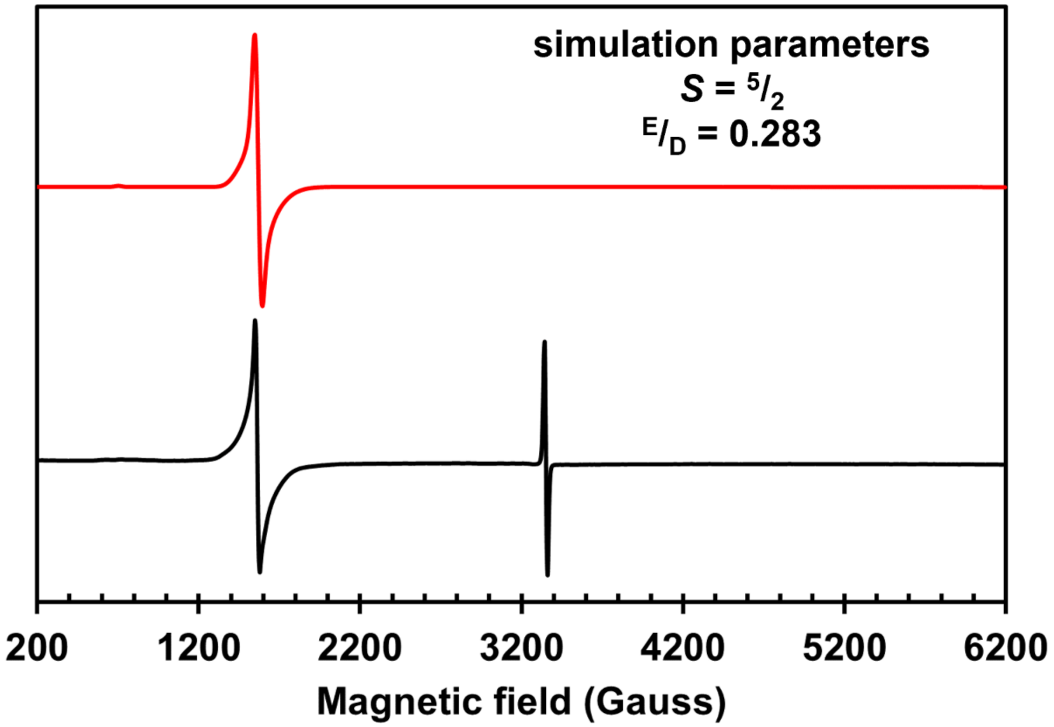

Figure S-51. Frozen toluene EPR spectrum of the reaction of [(*m*-CF_3-Py)Fe_2(^{Ph}Dbf)$_2$] (**6a**) with 2 equiv. $FcPF_6$ at 80 K (black). The red line represents a simulation with VisualRhombo.

Electrochemistry - Cyclic Voltammetry (CV)

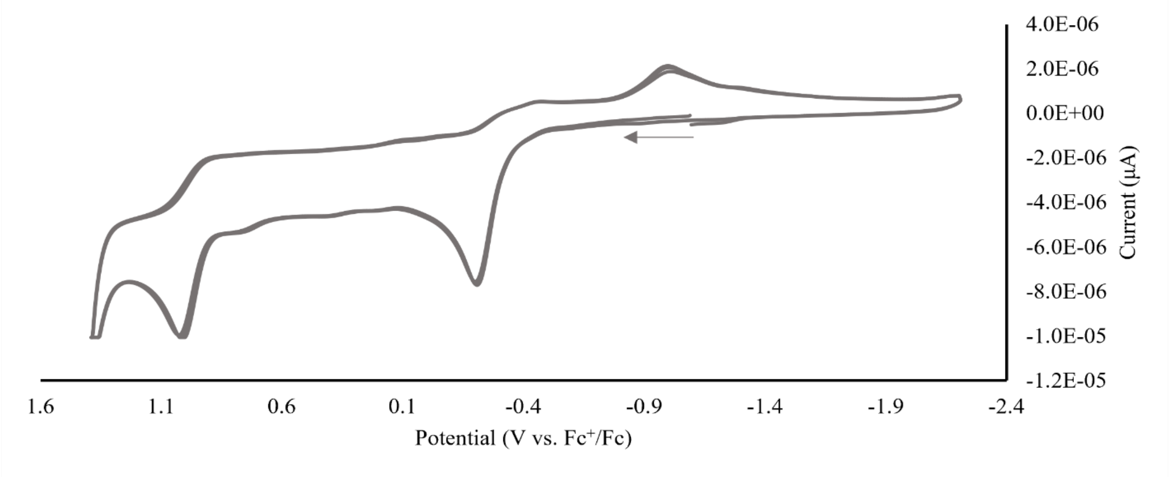

Figure S-52. Cyclic voltammetry spectra for $[Fe_2(^{Ph}Dbf)_2]$ (**1a**) in a 0.3 M $TBAPF_6$ 1,2-difluorobenzene solution referenced against Fc^+/Fc and an internal standard of 0.1 M $AgNO_3$ in ACN, scanning in the positive direction at a scan rate of 100 mV/s (open circuit potential = –0.96 V)

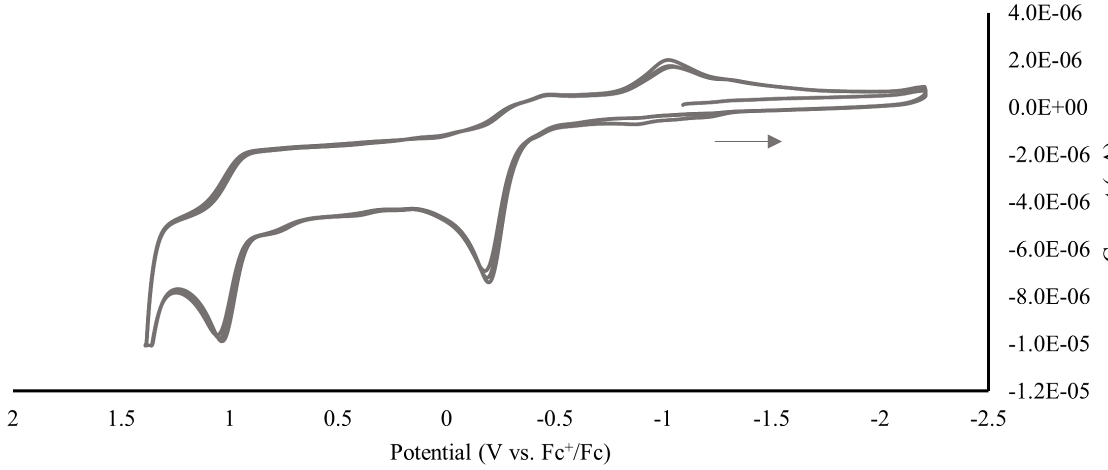

Figure S-53. Cyclic voltammetry spectra for $[Fe_2(^{Ph}Dbf)_2]$ (**1a**) in a 0.3 M $TBAPF_6$ 1,2-difluorobenzene solution referenced against Fc^+/Fc and an internal standard of 0.1 M $AgNO_3$ in ACN, scanning in the negative direction at a scan rate of 100 mV/s.

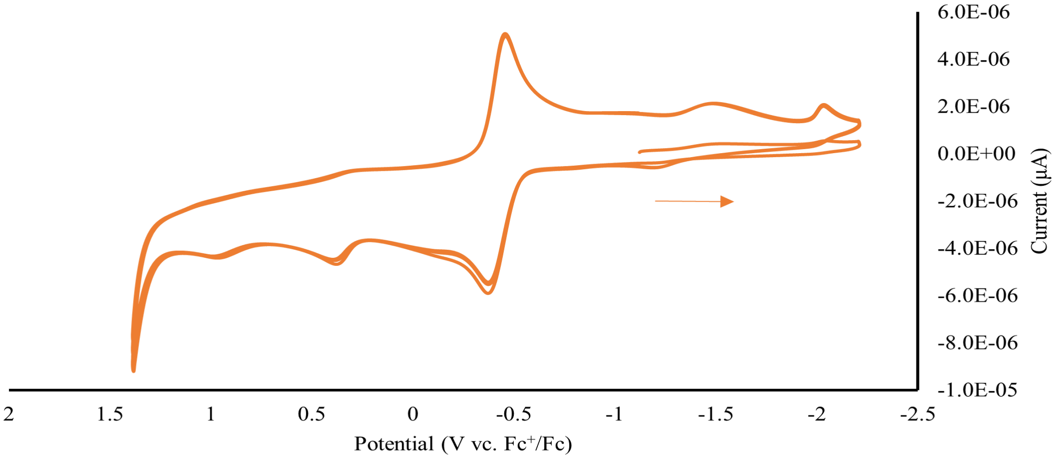

Figure S-54. Cyclic voltammetry spectra for [$Py_2Fe(^{Ph}Dbf)$] (**2a**) in a 0.3 M $TBAPF_6$ 1,2-difluorobenzene solution referenced against Fc^+/Fc and an internal standard of 0.1 M $AgNO_3$ in ACN, scanning in the negative direction at a scan rate of 100 mV/s (open circuit potential = –0.87 V).

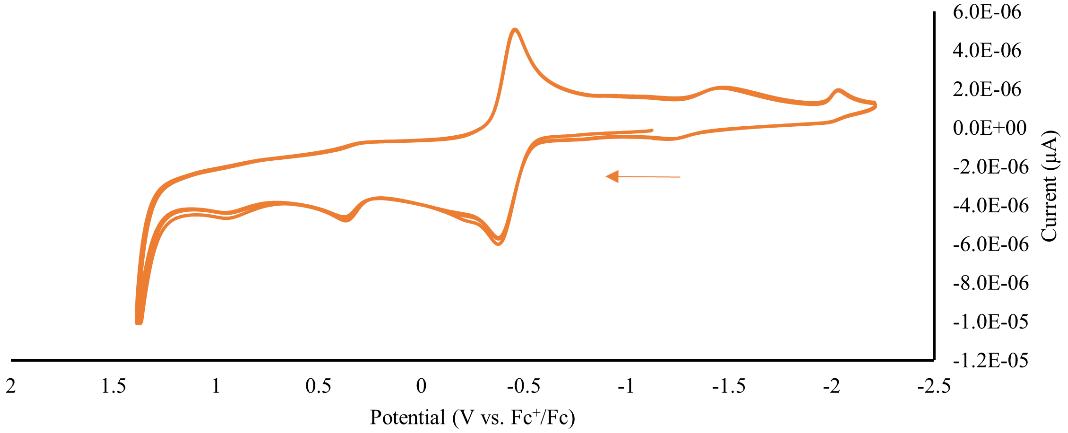

Figure S-55. Cyclic voltammetry spectra for [$Py_2Fe(^{Ph}Dbf)$] (**2a**) in a 0.3 M $TBAPF_6$ 1,2-difluorobenzene solution referenced against Fc^+/Fc and an internal standard of 0.1 M $AgNO_3$ in ACN, scanning in the positive direction at a scan rate of 100 mV/s.

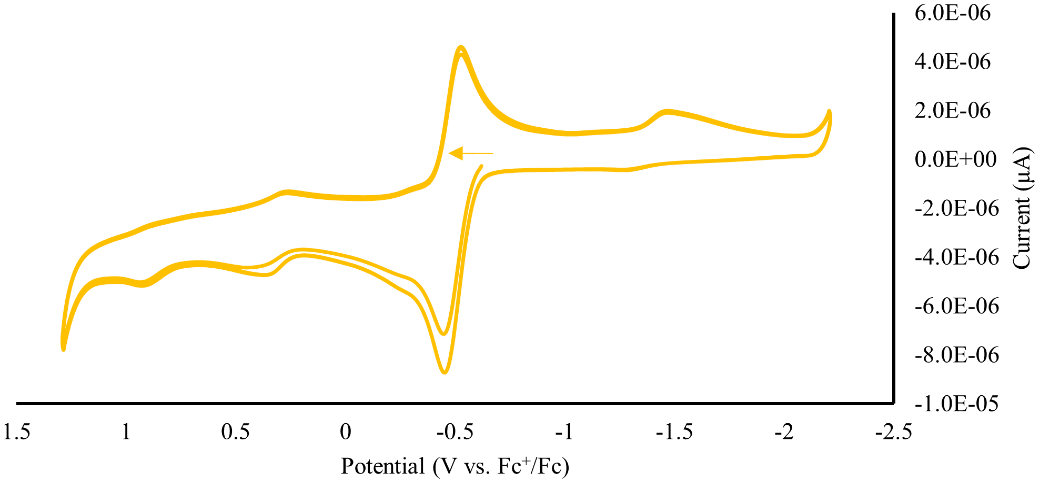

Figure S-56. Cyclic voltammetry spectra for [(*p*-^{t}Bu-Py)$_2$Fe(^{Ph}Dbf)] (**3a**) in a 0.3 M TBAPF$_6$ 1,2-difluorobenzene solution referenced against Fc$^+$/Fc and an internal standard of 0.1 M AgNO$_3$ in ACN, scanning in the positive direction at a scan rate of 100 mV/s (open circuit potential = –0.77 V).

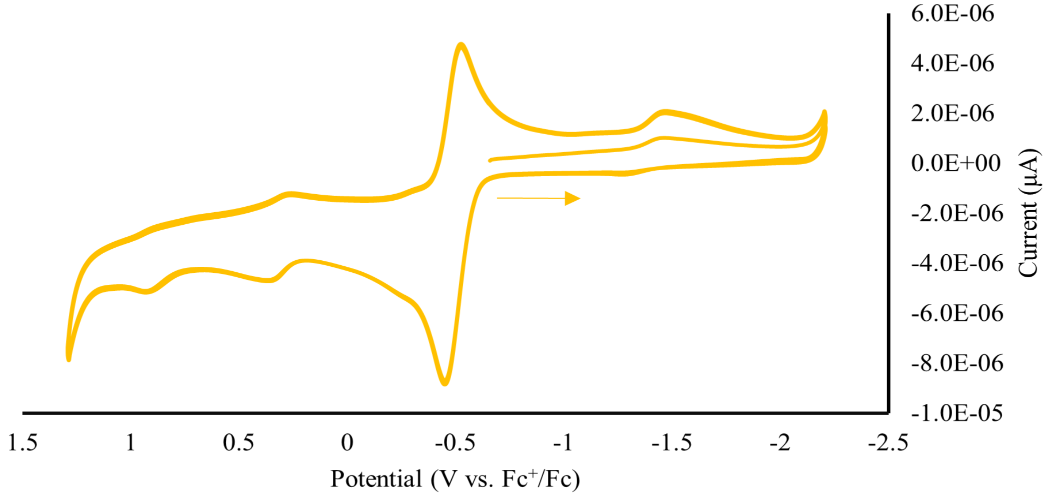

Figure S-57. Cyclic voltammetry spectra for [(*p*-^{t}Bu-Py)$_2$Fe(^{Ph}Dbf)] (**3a**) in a 0.3 M TBAPF$_6$ 1,2-difluorobenzene solution referenced against Fc$^+$/Fc and an internal standard of 0.1 M AgNO$_3$ in ACN, scanning in the negative direction at a scan rate of 100 mV/s.

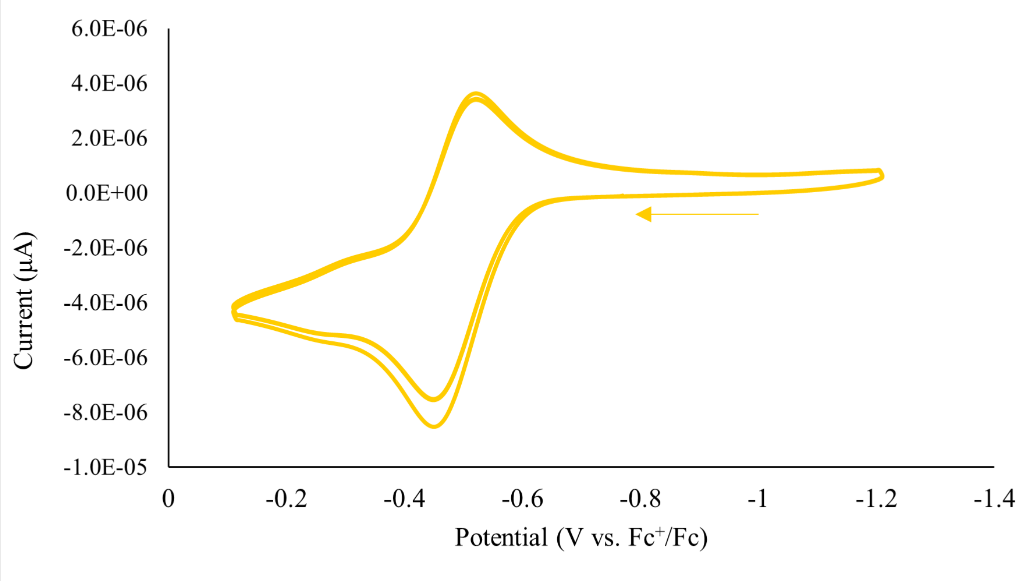

Figure S-58. Cyclic voltammetry spectra for [(*p*-^{t}Bu-Py)$_2$Fe(^{Ph}Dbf)] (**3a**) in a 0.3 M $TBAPF_6$ 1,2-difluorobenzene solution referenced against Fc^+/Fc and an internal standard of 0.1 M $AgNO_3$ in ACN, scanning in the positive direction at a scan rate of 100 mV/s.

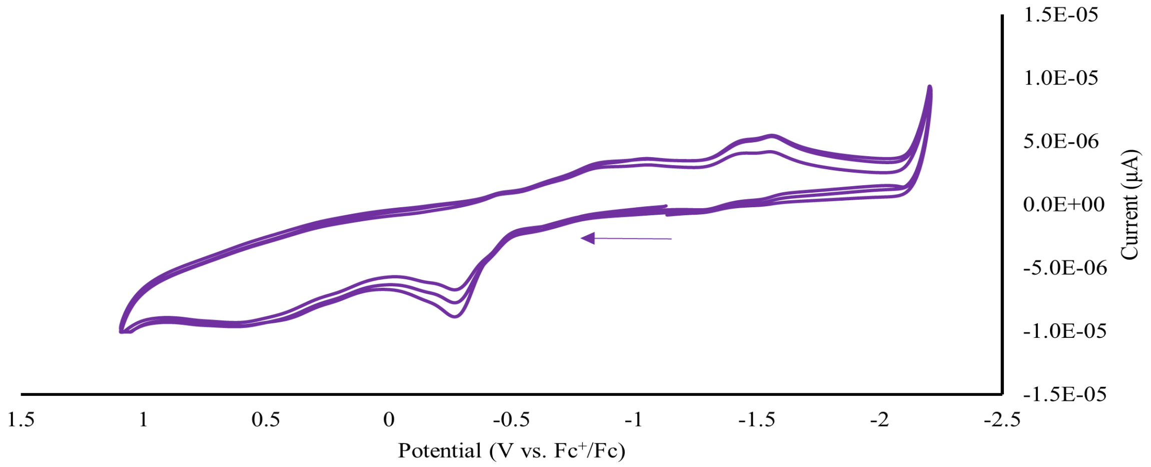

Figure S-59. Cyclic voltammetry spectra for [(*p*-CF_3-Py)$_2$Fe(^{Ph}Dbf)] (**4a**) in a 0.3 M $TBAPF_6$ 1,2-difluorobenzene solution referenced against Fc^+/Fc and an internal standard of 0.1 M $AgNO_3$ in ACN, scanning in the positive direction at a scan rate of 100 mV/s. A single irreversible oxidation peak is observed at –0.275 V (open circuit potential = –1.15 V).

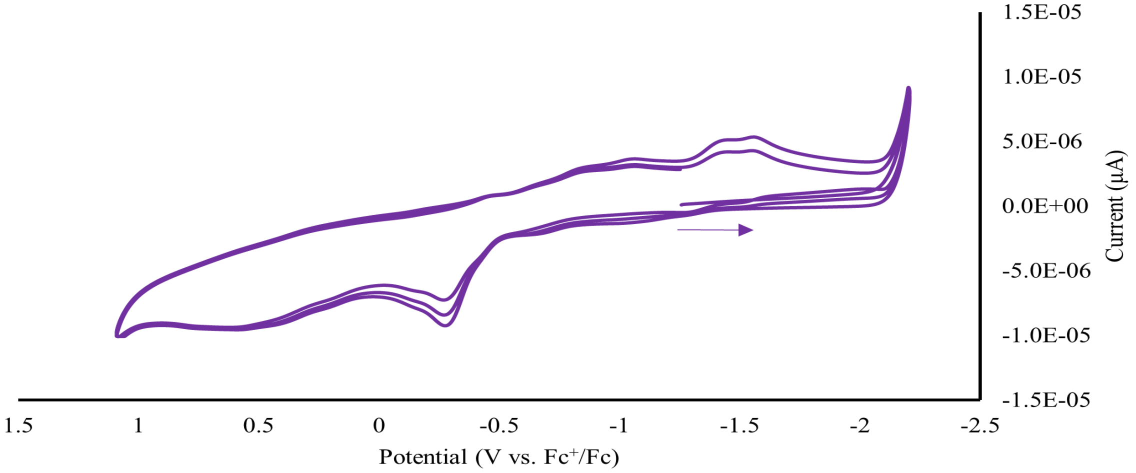

Figure S-60. Cyclic voltammetry spectra for [(*p*-CF_3-Py)$_2$Fe(^{Ph}Dbf)] (**4a**) in a 0.3 M $TBAPF_6$ 1,2-difluorobenzene solution referenced against Fc^+/Fc and an internal standard of 0.1 M $AgNO_3$ in ACN, scanning in the negative direction at a scan rate of 100 mV/s. (E_{pa} = –0.275 V)

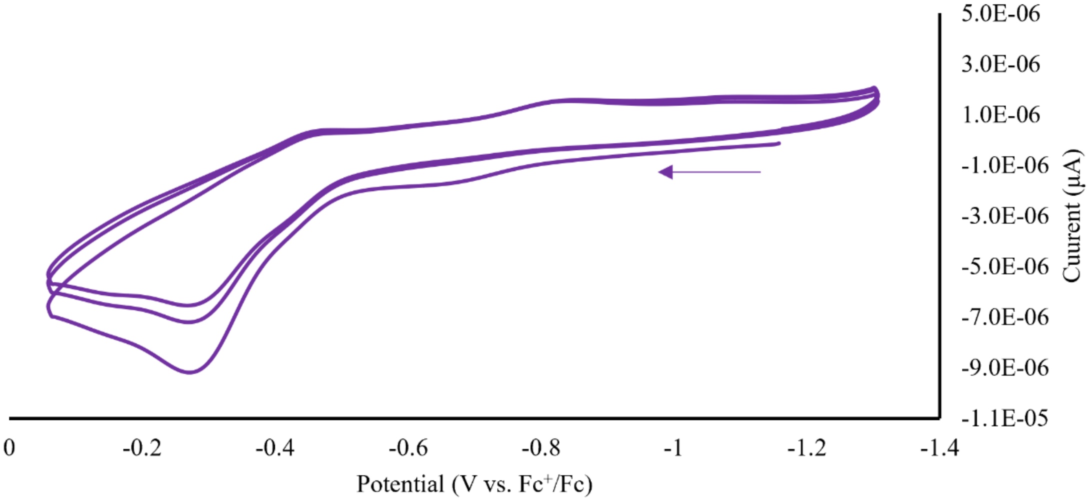

Figure S-61. Cyclic voltammetry spectra for [(*p*-CF_3-Py)$_2$Fe(^{Ph}Dbf)] (**4a**) in a 0.3 M $TBAPF_6$ 1,2-difluorobenzene solution referenced against Fc^+/Fc and an internal standard of 0.1 M $AgNO_3$ in ACN, scanning in the negative direction at a scan rate of 100 mV/s. (E_{pa} = –0.275 V)

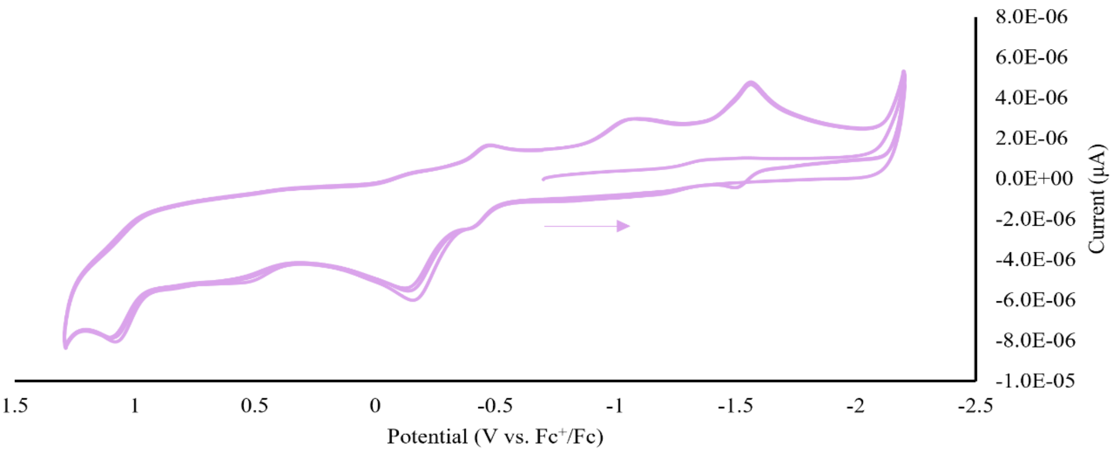

Figure S-62. Cyclic voltammetry spectra for [(*p*-CF_3-Py)Fe_2(^{Ph}Dbf)$_2$] (**5a**) in a 0.3 M $TBAPF_6$ 1,2-difluorobenzene solution referenced against Fc^+/Fc and an internal standard of 0.1 M $AgNO_3$ in ACN, scanning in the negative direction at a scan rate of 100 mV/s (open circuit potential = –0.83 V). The general shape of the data is very similar to that of [Fe_2(^{Ph}Dbf)$_2$] (**1a**), suggestive of a dimeric species as expected. Compared to the CV for **4a**, both reduction peaks appear to be unique to the dimeric structure, as well as the second oxidation (1.1 V).

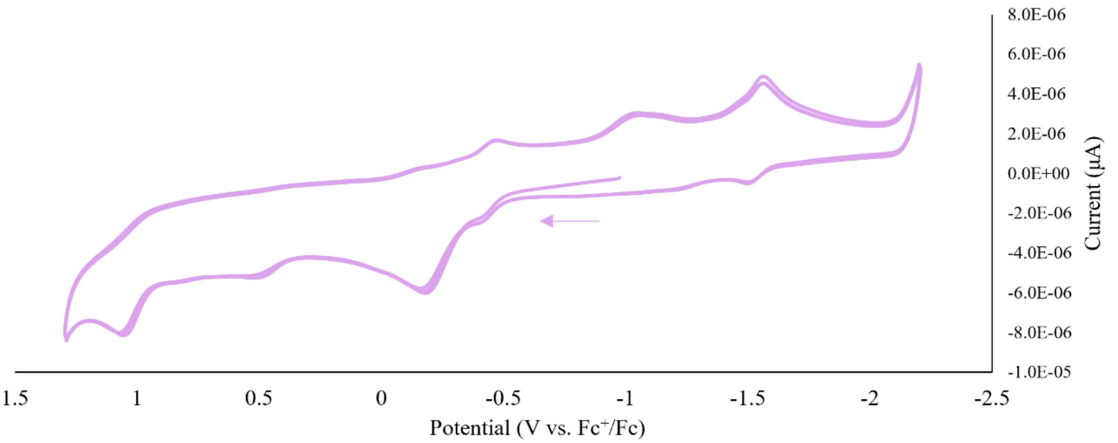

Figure S-63. Cyclic voltammetry spectra for [(*p*-CF_3-Py)Fe_2(^{Ph}Dbf)$_2$] (**5a**) in a 0.3 M $TBAPF_6$ 1,2-difluorobenzene solution referenced against Fc^+/Fc and an internal standard of 0.1 M $AgNO_3$ in ACN, traveling in the positive direction at a scan rate of 100 mV/s.

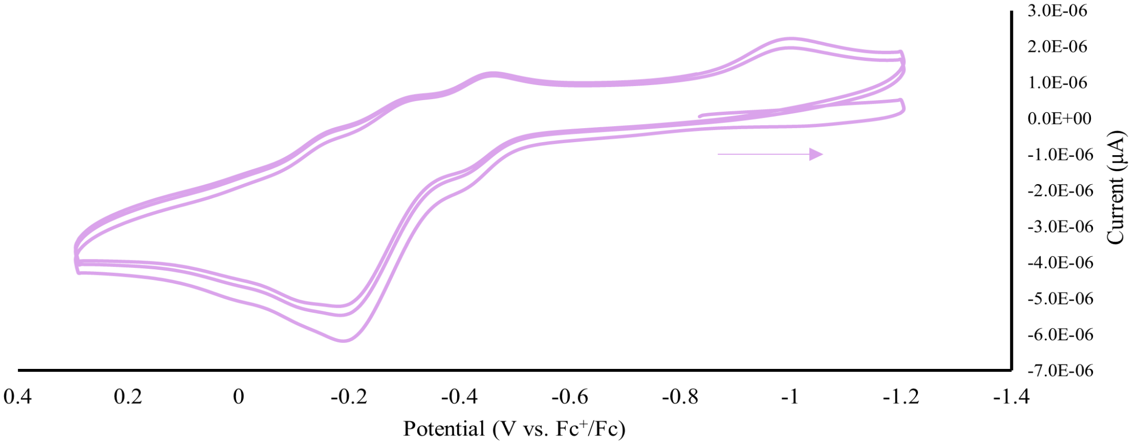

Figure S-64. Cyclic voltammetry spectra for [(p-CF_3-Py)Fe_2(^{Ph}Dbf)$_2$] (**5a**) in a 0.3 M $TBAPF_6$ 1,2-difluorobenzene solution referenced against Fc^+/Fc and an internal standard of 0.1 M $AgNO_3$ in ACN, scanning in the negative direction at a scan rate of 100 mV/s. The appearance of the signal at -1.00 V only after oxidation (-0.195 V) suggests a dependence on the initial oxidation of the complex.

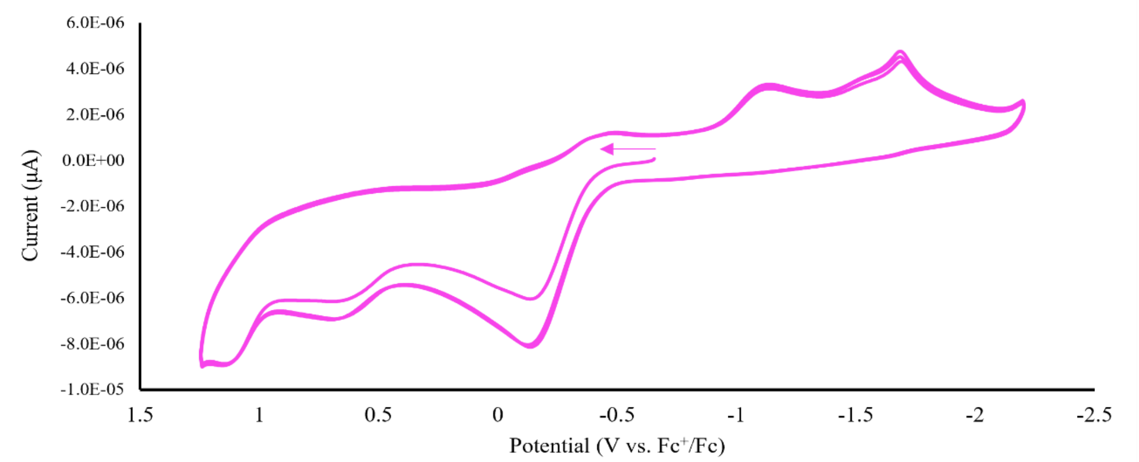

Figure S-65. Cyclic voltammetry spectra for [(m-CF_3Py)Fe_2(^{Ph}Dbf)$_2$] (**6a**) in a 0.3 M $TBAPF_6$ 1,2-difluorobenzene solution referenced against Fc^+/Fc and an internal standard of 0.1 M $AgNO_3$ in ACN, scanning in the positive direction at a scan rate of 100 mV/s (open circuit potential = – 0.67 V).

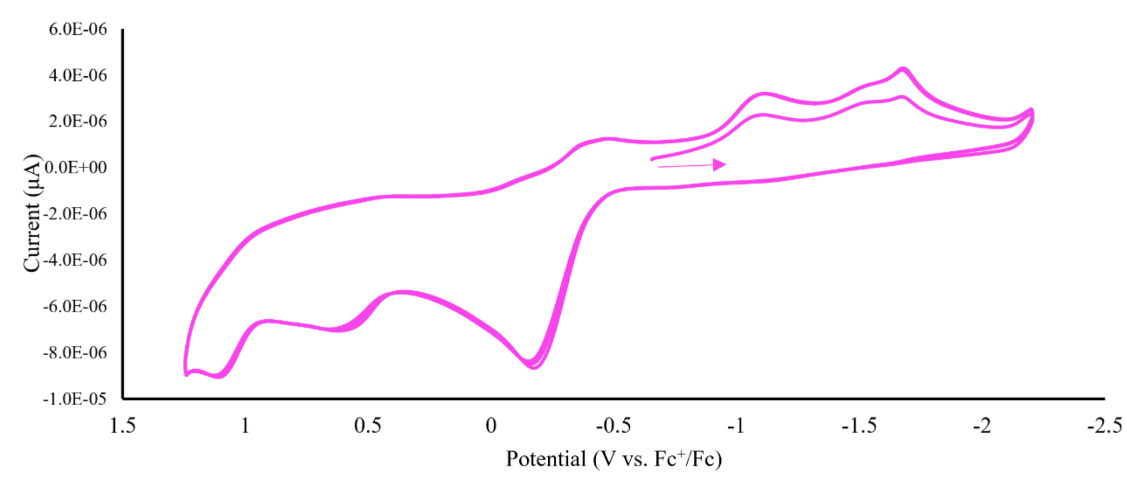

Figure S-66. Cyclic voltammetry spectra for [(*m*-CF_3Py)Fe_2(^{Ph}Dbf)$_2$] (**6a**) in a 0.3 M $TBAPF_6$ 1,2-difluorobenzene solution referenced against Fc^+/Fc and an internal standard of 0.1 M $AgNO_3$ in ACN, scanning in the negative direction at a scan rate of 100 mV/s.

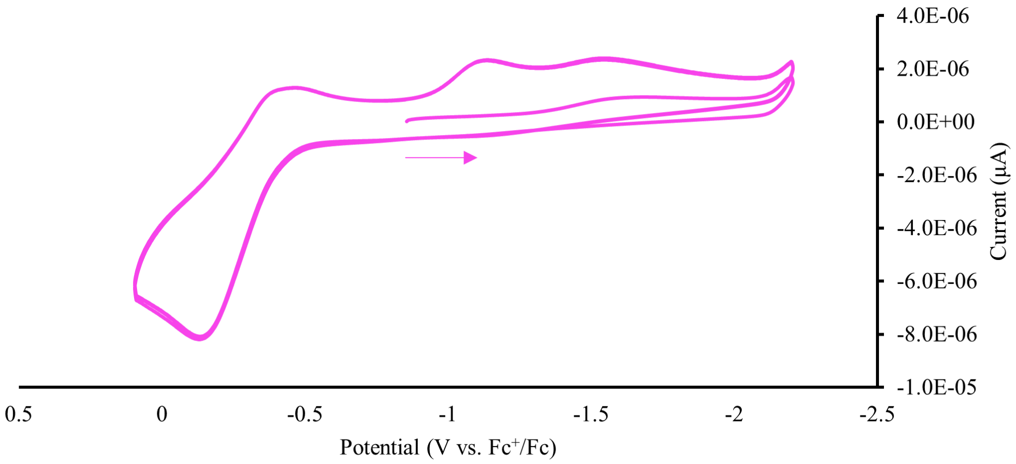

Figure S-67. Cyclic voltammetry spectra for [(*m*-CF_3Py)Fe_2(^{Ph}Dbf)$_2$] (**6a**) in a 0.3 M $TBAPF_6$ 1,2-difluorobenzene solution referenced against Fc^+/Fc and an internal standard of 0.1 M $AgNO_3$ in ACN, scanning in the negative direction at a scan rate of 100 mV/s. The appearance of signals at -1.14 V and -1.545 V only after oxidation of the complex at -1.45 V suggests that these reduction signals are dependent on the initial oxidation of the complex.

Computational results

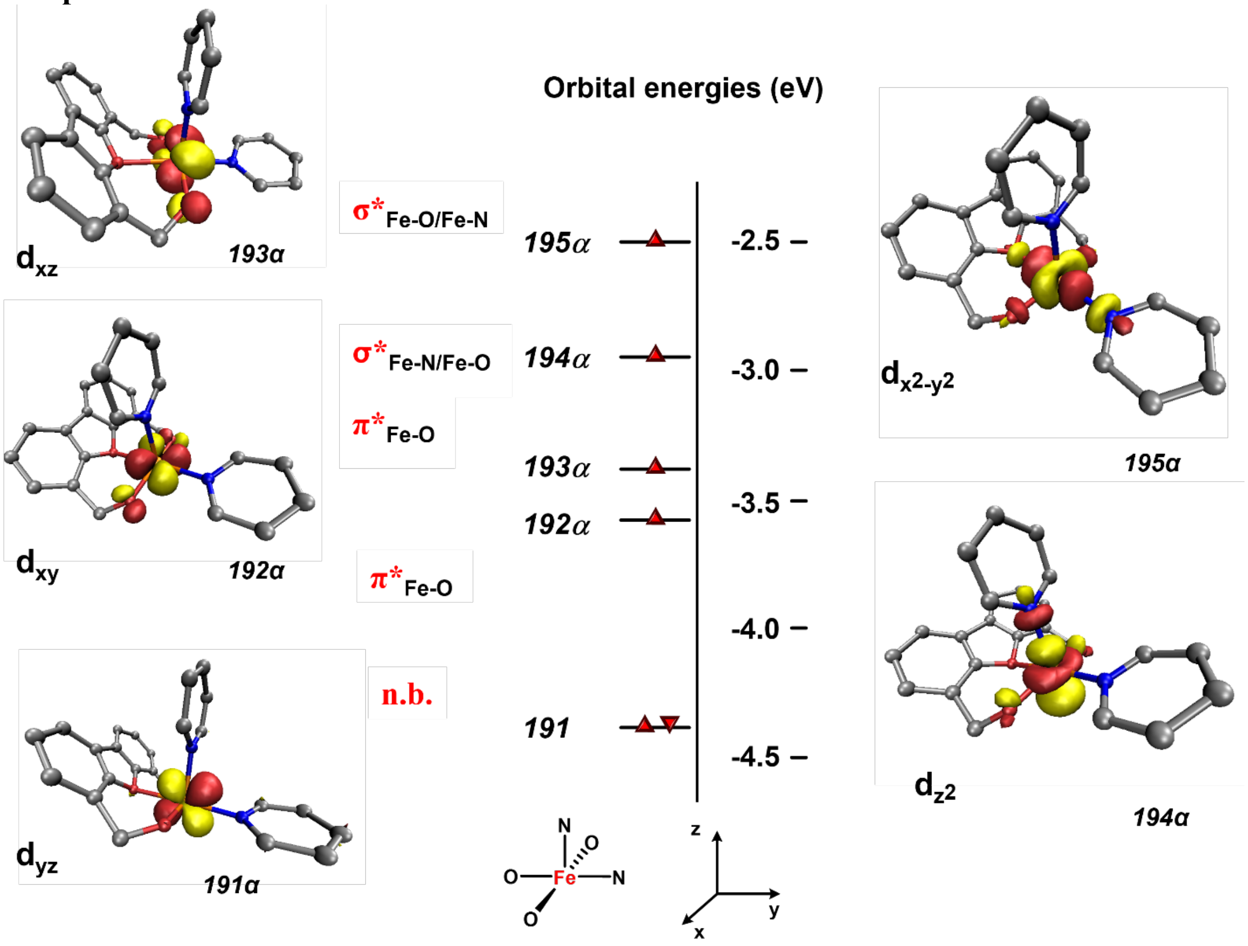

Figure S-68. Frontier molecular orbitals for [$Py_2Fe(^{Ph}Dbf)$] (**2a**) (isovalue 0.04). Quasi-restricted orbitals (α-spins) are shown.

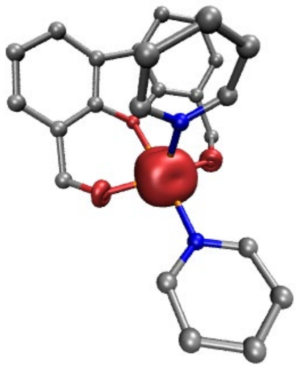

Figure S-69. Spin density plot for [$Py_2Fe(^{Ph}Dbf)$] (**2a**) (isovalue 0.03).

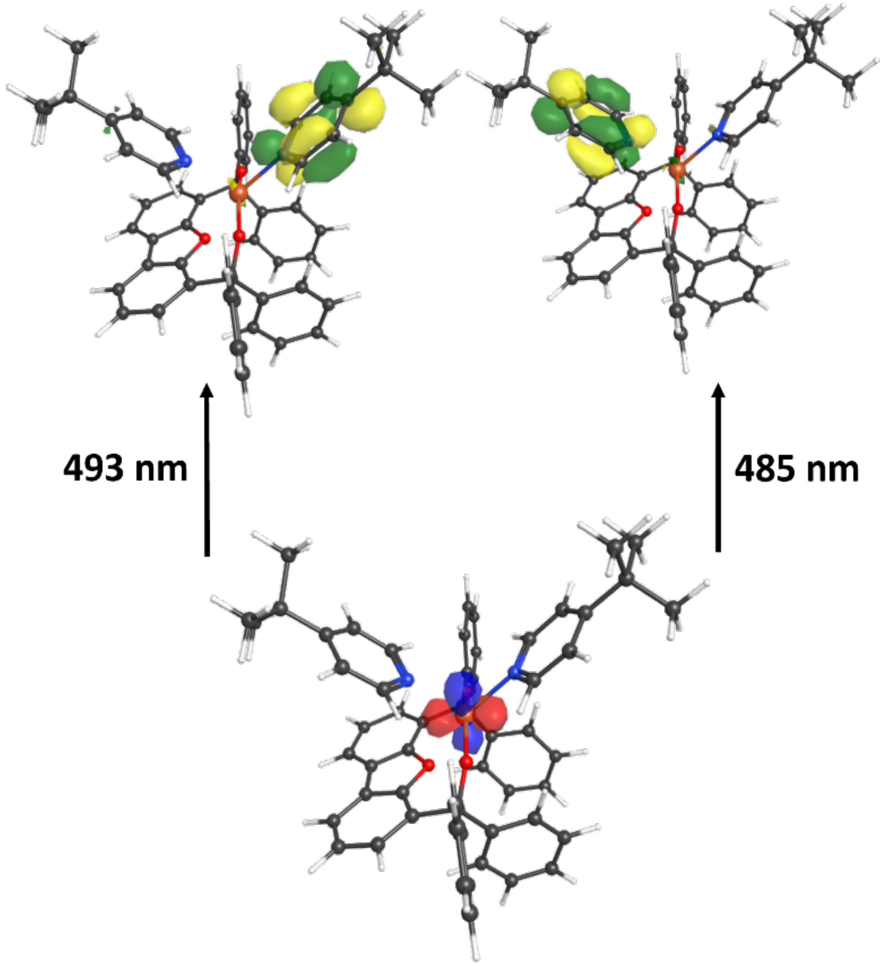

Figure S-70. Natural transition orbitals for [(p-^{t}Bu-Py)$_2$Fe(^{Ph}Dbf)] (**3a**) illustrating the MLCT transitions from the non-bonding d_{yz} orbital into the 4-*tert*-butylpyridine lowest π* orbital.

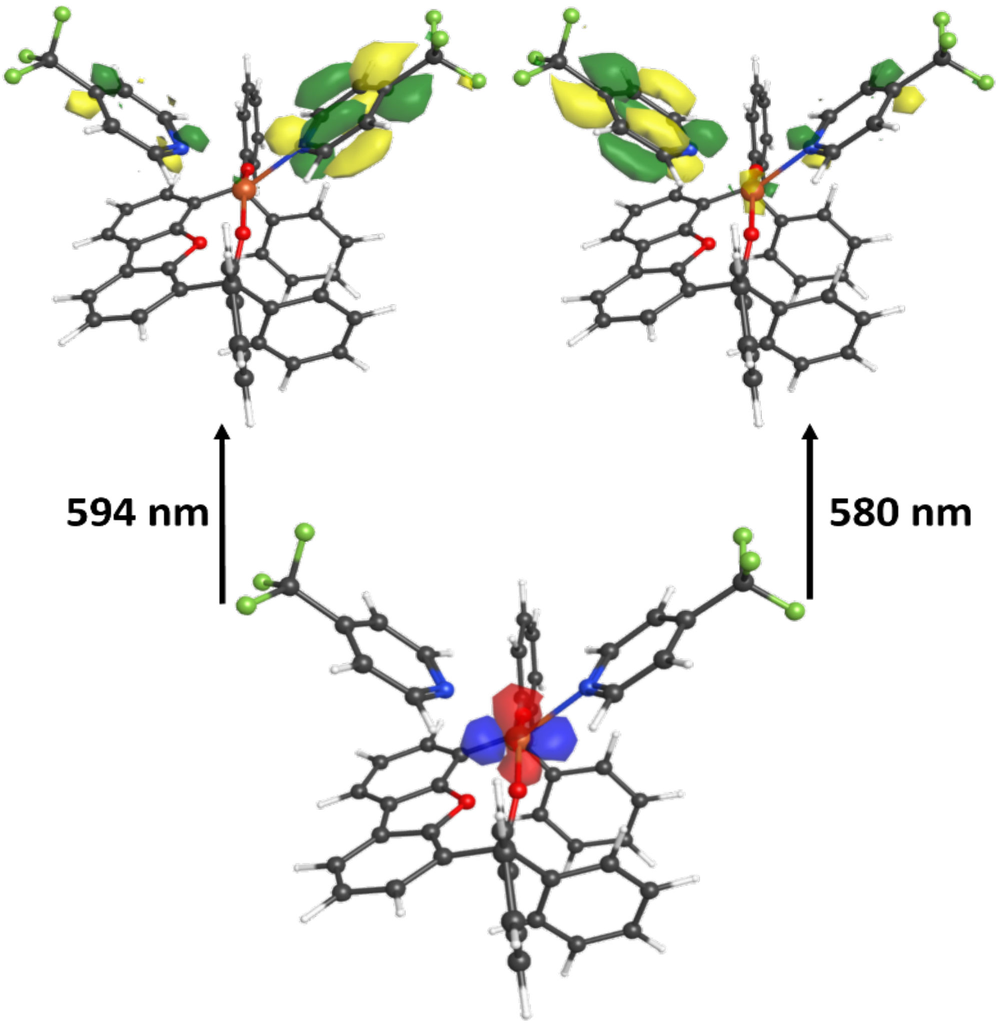

Figure S-71. Natural transition orbitals for [(*p*-CF_3-Py)$_2$Fe(^{Ph}Dbf)] (**4a**) illustrating the MLCT transitions from the non-bonding d_{yz} orbital into the 4-trifluoromethylpyridine lowest π^* orbital.

Table S-1. Representative bond metrics in **2a – 4a.**

	**$Py_2Fe(^{Ph}Dbf)$ (2a)**	**(p-^{t}Bu-Py)$_2$Fe(^{Ph}Dbf) (3a)**	**(p-CF_3-Py)$_2$Fe(^{Ph}Dbf) (4a)**
**Fe–O (Å)**	1.9106(8) / 1.9150(8)	1.9082(9) / 1.9148(9)	1.9087 (14) / 1.9117(14)
**Fe–O_{furan} (Å)**	2.3381(8)	2.3192(9)	2.3152(13)
**Fe–N (Å)**	2.1516(10) / 2.1518(10)	2.1304(11) / 2.1697(12)	2.1338(16) / 2.1618(17)
**O_{furan}–Fe–N (°)**	176.33(3) / 87.45(3)	169.92(4) / 87.53(4)	169.84(6) / 85.97(5)
**O–Fe–O (°)**	158.29(4)	159.86(4)	160.92(6)
**O_{furan}–Fe–O (°)**	83.35(3) / 83.28(3)	84.00(3) / 82.43(3)	84.44(5) / 83.68(5)
**O–Fe–N (°)**	100.25(4) / 93.30(4)<br>99.51(4) / 96.16(4)	98.31(4) / 96.37(4)<br>95.90(4) / 94.44 (4)	97.10(6) / 93.99(6)<br>96.89(6) / 95.08(6)
**N–Fe–N (°)**	94.48(4)	102.35(4)	104.19(6)

Table S-2. Pyridine bond metrics in **2a** compared to those for free pyridine (reported in Å)

	**2a** (X-ray)		**2a** (optimized)		Optimized	
	**axial pyridine**	**equatorial pyridine**	**axial pyridine**	**equatorial pyridine**	**pyridine**	**pyridine radical anion**
**N–C1**	1.339	1.329	1.341	1.341	1.335	1.382
**C1–C2**	1.389	1.392	1.394	1.394	1.398	1.379
**C2–C3**	1.390	1.371	1.397	1.397	1.397	1.439
**C3–C4**	1.373	1.375	1.396	1.397	1.397	1.438
**C4–C5**	1.383	1.389	1.394	1.395	1.399	1.379
**C5–N**	1.337	1.338	1.341	1.341	1.334	1.383

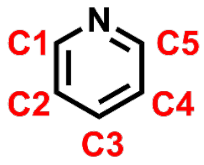

Table S-3. Optimized XYZ coordinates for [$Py_2Fe(^{Ph}Dbf)$] (**2a**) (in Å)

Fe	9.1800516936	12.3574026955	4.3858937649
O	7.3096285273	12.7722215158	4.2621087862
O	10.8623934639	11.5402364845	3.9562197279
O	9.0054560501	12.4481688679	2.0353942927
N	9.1789386686	11.8172930679	6.5111884401
N	9.9062798013	14.4456948373	4.5709687779
C	8.0874420734	12.0243031622	7.2631179386
C	8.0297770614	11.6689223830	8.6103272466
C	9.1476509141	11.0727181970	9.1983346775
C	10.2800390757	10.8536599855	8.4106633253
C	10.2525857563	11.2398281129	7.0709292539
C	9.0396704407	15.4683448462	4.6098374477
C	9.4502611523	16.7972034870	4.7098711511
C	10.8168936949	17.0772803068	4.7694476443
C	11.7181421966	16.0116112242	4.7208407071
C	5.8574810508	10.7924028383	1.5481157955
C	14.9174397937	9.2747027438	2.6545259310
C	4.9987802173	13.2641563078	3.8172604773
C	8.6526787930	14.0751857181	0.4637186189
C	11.2192419215	14.7136790444	4.6189505997
C	6.3326365448	12.6883839759	3.2638308655
C	2.6070388170	14.3660463178	4.8495367534
C	6.7963363806	13.5137703142	2.0309136724
C	8.0888207886	13.3423169258	1.5231171874
C	6.0892375725	11.2080649514	2.8663353530
C	11.3755877639	11.9565926370	1.6412239384
C	3.8372114294	14.6676453796	5.4393594472
C	10.6459172395	9.6502058397	2.4518099111
C	9.7191045488	8.0569889277	0.8569931011
C	10.0289648870	13.6094211266	0.3484101395
C	6.0492350188	10.2423043474	3.8850933915
C	3.7550842599	12.9632856922	3.2377829559
C	10.1859623834	12.6310197196	1.3459043503
C	11.4201479808	10.9446029134	2.8197603959
C	12.8922167466	10.5507730213	3.1280830547
C	9.5887819954	7.5939731237	3.2201579900
C	6.0473880284	14.4947802698	1.3641408847
C	9.3250130011	7.2165216931	1.8988208235
C	5.7822735883	8.9037552734	3.5956020052
C	5.0182446458	14.1208102507	4.9271550611
C	14.8958452171	10.8375660182	4.4897173846
C	13.5670983980	11.1598793689	4.1952497768
C	15.5792671875	9.8935742461	3.7191211895
C	13.5882342358	9.5980796601	2.3656347075

C	7.8653997957	15.0411671954	-0.1802548437
C	5.5506717795	8.4999255516	2.2760091813
C	5.5914815070	9.4492786556	1.2541728232
C	11.1325995965	13.9362592777	-0.4533411404
C	12.4551627719	12.3206499798	0.8231163440
C	10.2405194511	8.7974980879	3.4913242405
C	2.5722728684	13.5097414906	3.7450750695
C	12.3365459041	13.2800830254	-0.2024226179
C	10.3752539656	9.2631551210	1.1322513003
C	6.5643849887	15.2343292093	0.2815099365
H	7.2410546890	12.4814308701	6.7448188956
H	7.1188132653	11.8561279934	9.1824231074
H	9.1352867889	10.7806366508	10.2513670301
H	11.1766716089	10.3859210633	8.8223632372
H	11.1025637392	11.0830527126	6.4023415875
H	7.9856959437	15.1914503326	4.5482655730
H	8.7046430763	17.5944109833	4.7348951244
H	11.1734386380	18.1074588913	4.8469906195
H	12.7968263043	16.1770166130	4.7550733293
H	5.8843398436	11.5200630519	0.7344328432
H	15.4352889558	8.5271121520	2.0473702424
H	11.8788575751	13.8461734592	4.5602837881
H	1.6820968048	14.7891686859	5.2503146455
H	3.8792809761	15.3310910433	6.3079926570
H	9.5132416989	7.7790237288	-0.1803098431
H	6.2277898883	10.5558585839	4.9149081479
H	3.7048990731	12.2846863143	2.3840711505
H	9.2829317053	6.9451625393	4.0455220785
H	5.0293461374	14.6987023146	1.6976235115
H	8.8109368698	6.2757746961	1.6853683618
H	5.7551968687	8.1690119189	4.4050202511
H	5.9779625831	14.3432314046	5.3940746803
H	15.3986658572	11.3254262534	5.3297218151
H	13.0265169265	11.8857100366	4.8028354440
H	16.6167510891	9.6370162038	3.9492777157
H	13.0847280194	9.0919446265	1.5397236214
H	8.2611838275	15.6305325190	-1.0105376089
H	5.3452435737	7.4509181624	2.0475127258
H	5.4199974277	9.1473743778	0.2173244687
H	11.0548959783	14.6875684243	-1.2425541109
H	13.4243312253	11.8486343897	0.9874703571
H	10.4468096854	9.0894895232	4.5222559692
H	1.6167139715	13.2565767120	3.2776246772
H	13.2164327587	13.5167140714	-0.8059107518
H	10.6763787202	9.9064334823	0.3029290513
H	5.9274822710	15.9830458002	-0.1961736305

Table S-4. Optimized XYZ coordinates for [(p-^{t}Bu-Py)$_2$Fe(^{Ph}Dbf)] (**3a**) (in Å)

Fe	-0.3543968755	-0.0606479502	0.1127642356
O	1.4183991531	0.2880278442	-1.4098947108
O	0.0014299226	-1.8998445098	-0.3231768926
O	-0.6043826637	1.8203766564	-0.1822218989
N	0.9798494215	0.0735476453	1.8762943515
N	-2.2600210303	-0.4058829004	1.1351712513
C	2.4011956961	-0.6793418088	-1.4251444722
C	2.1442505945	-2.0547131038	-1.4081993899
C	-0.6609434872	4.1687818788	-0.6906675760
C	-0.0332055219	-2.3415288118	-2.7074599954
C	2.0491367986	1.5119627453	-1.3323896646
C	3.2996671687	-2.8501079035	-1.3994044326
C	-0.1763735314	2.7572765959	-1.1276098408
C	1.6166007719	-1.0138428544	2.3377770814
C	-0.7945275944	2.4461150653	-2.5173733242
C	0.6783808154	-2.5685096705	-1.3468810915
C	0.6619728518	-4.0949467435	-1.0478377735
C	-0.0429905237	-2.0327222751	-5.1249700331
C	3.6768988523	-0.0878619722	-1.4110561070
C	1.3757979655	2.7322646969	-1.2084803958
C	0.6530162977	-2.1747374134	-3.9180277647
C	-1.4368551822	-2.3570432905	-2.7345400037
C	2.2252272399	3.8457409714	-1.1260225165
C	3.4459884139	1.3497171418	-1.3513918493
C	-2.1384232102	2.0428857508	-2.5729780646
C	-1.4381918246	-2.0559804282	-5.1394969868
C	0.3931030881	-5.9093359620	0.5603102218
C	-3.0567048165	0.6181399863	1.4785865235
C	0.6438243978	-6.8564511052	-0.4358088343
C	0.4021561431	-4.5445169847	0.2544578469
C	4.2576808027	2.4911593250	-1.2763325941
C	3.6291839033	3.7305004547	-1.1668804692
C	-0.0946918689	2.5913790076	-3.7229554811
C	-2.1330846128	-2.2171582401	-3.9356576002
C	-0.7596607510	5.2408406961	-1.5927958833
C	-2.6781036841	-1.6455868973	1.4161513052
C	-1.5193484305	6.7223462001	0.1711135766
C	4.8054334530	-0.9210480064	-1.4141871690
C	4.5968996211	-2.2992631096	-1.4104146447
C	0.8990544594	-6.4225293682	-1.7401124915
C	1.2286240547	1.2407203691	2.4810884724
C	-0.7188314235	2.3408085394	-4.9514182664
C	-1.1806613498	6.5045520415	-1.1676214582
C	0.9040215443	-5.0573930039	-2.0420034616
C	2.5043908972	-0.9714297616	3.4067425363

C	-3.8894553396	-1.9099141463	2.0556086425
C	-2.0554945339	1.9430632632	-4.9933407061
C	-2.7635547305	1.7945343300	-3.7954770625
C	-4.7342466615	-0.8558161209	2.4338525168
C	-4.2775942368	0.4373913434	2.1191670474
C	-6.0806526807	-1.0619337077	3.1458681066
C	-1.4327788151	5.6619789474	1.0769556483
C	2.1044233267	1.3681301291	3.5594629324
C	-1.0086291827	4.4001859837	0.6478210122
C	-6.3846690572	-2.5510474102	3.3884406806
C	-6.0443829538	-0.3395299124	4.5128816803
C	2.7784838154	0.2442955112	4.0587338955
C	-7.2118669756	-0.4656547229	2.2760866563
C	5.1558676919	-0.1798941983	4.7461557366
C	3.9151950811	1.7220527409	5.8008263043
C	3.7681983470	0.2990647938	5.2333000258
C	3.2677742996	-0.6308144312	6.3630582859
H	3.1929844974	-3.9350329770	-1.3777635939
H	1.4036096135	-1.9435550234	1.8073400340
H	0.5134657151	-1.8986011759	-6.0566608913
H	1.7447664509	-2.1527136226	-3.9268008906
H	-1.9788232714	-2.4818510128	-1.7956760237
H	1.7859601584	4.8383888857	-1.0223761938
H	-2.6912537671	1.9250269489	-1.6395097450
H	-1.9826802166	-1.9429358144	-6.0805928499
H	0.1848285224	-6.2339388968	1.5839098323
H	-2.6863218653	1.6110732448	1.2127253544
H	0.6341362708	-7.9240437955	-0.2004448257
H	0.1929305223	-3.8025724552	1.0253668877
H	5.3470964045	2.4118498271	-1.2936056531
H	4.2337746784	4.6385861940	-1.1009306738
H	0.9521794598	2.9016549830	-3.7116031911
H	-3.2265011832	-2.2325931074	-3.9336448165
H	-0.5134978645	5.0871839828	-2.6452606753
H	-2.0088186692	-2.4501058248	1.1019201092
H	-1.8539674251	7.7087191082	0.5032881374
H	5.8153967889	-0.5046795504	-1.4087179870
H	5.4556609937	-2.9753003274	-1.4061939385
H	1.0876502019	-7.1514438322	-2.5331408032
H	0.7099835870	2.1081099542	2.0684804472
H	-0.1504926700	2.4544614147	-5.8786370048
H	-1.2519637033	7.3215615371	-1.8909310296
H	1.0894289579	-4.7401360194	-3.0700884780
H	2.9859907250	-1.9000857199	3.7177565369
H	-4.1543943361	-2.9493107565	2.2461635646
H	-2.5430381405	1.7445453480	-5.9513531774

H	-3.8109207159	1.4807424953	-3.8149064010
H	-4.8688011849	1.3207926302	2.3670810567
H	-1.7005689421	5.8158271213	2.1263081023
H	2.2509905746	2.3588190987	3.9881942193
H	-0.9531524717	3.5665204124	1.3481873195
H	-6.4444236427	-3.1178096682	2.4462132744
H	-7.3556889906	-2.6538719727	3.8972686121
H	-5.6252110957	-3.0273824787	4.0280167486
H	-5.2437551424	-0.7426199588	5.1531899372
H	-7.0023676192	-0.4741408856	5.0409630753
H	-5.8744473786	0.7422436117	4.4025000589
H	-7.0760009614	0.6130694436	2.1055548438
H	-8.1861392617	-0.6046682635	2.7720765011
H	-7.2577185209	-0.9587250998	1.2922268952
H	5.1253947201	-1.2120706277	4.3656263867
H	5.8810317231	-0.1506737564	5.5755917705
H	5.5362426493	0.4648146886	3.9382069960
H	4.2956324513	2.4281958839	5.0464238410
H	4.6310916945	1.7157636933	6.6373729538
H	2.9602733906	2.1126417469	6.1860464370
H	2.2780477272	-0.3141006692	6.7286253804
H	3.9680485980	-0.6067705805	7.2137096348
H	3.1845276230	-1.6764313107	6.0297962273

Table S-5. Optimized XYZ coordinates for [(*p*-CF_3-Py)$_2$Fe(^{Ph}Dbf)] (**4a**) (in Å)

Fe	5.7524297455	9.0144225131	5.4484761106
F	1.6994834162	5.5986745305	10.1854650549
F	3.6689699006	4.9835753714	10.8344094763
F	2.8282338778	6.8416697495	11.5592547894
F	0.2489327300	13.4461540868	5.0480211736
F	1.6887366346	14.6805838714	4.0085173742
F	0.5490981833	13.1903119791	2.9195853867
O	7.2073607838	9.6865111756	3.7420392020
O	5.2359206428	7.7191609843	4.1374899953
O	6.8765786327	10.1552845194	6.4974872938
N	4.1978024552	10.5153831064	4.9774585316
N	4.7477868827	8.0535199165	7.1439123775
C	6.0896937593	8.3842740415	1.9880356511
C	6.7554679359	9.5257947803	2.4472729220
C	7.7243337392	10.9630715317	3.8400755130
C	7.6549679496	11.6200110393	2.5990447805
C	5.7015602827	8.4538947469	0.6418242939
C	5.8046144701	7.2086982597	2.9621859055
C	9.2229248071	9.5703422554	6.3217482343
C	8.4626993049	11.6206917888	7.5599374365
C	7.0199157632	10.6778207677	1.6860335202

C	3.5482091045	6.7751843959	9.3088001438
C	7.1129930645	6.4405823889	3.2878055233
C	3.3279767029	6.3236278844	8.0040818758
C	8.2054221559	11.5218951174	5.0289621900
C	8.1595119334	10.6998404285	6.3457950625
C	4.9613291058	8.4860842144	8.3948198561
C	7.4092043804	12.1279694010	8.3334637778
C	8.1300194006	12.9366642187	2.5075660580
C	4.8085178345	6.2078015588	2.3138875630
C	4.3810922234	7.8788745146	9.5071302022
C	5.9680037414	9.5788123327	-0.1640200848
C	3.9505323513	6.9915716677	6.9524377492
C	11.2891228766	8.5514416622	5.5270001188
C	2.9506558428	4.4308013710	1.1352228084
C	9.0945861642	8.5170026003	7.2417820718
C	3.2217650815	10.2377321264	4.1023654884
C	2.2285591362	11.1563217637	3.7663457077
C	6.6235105003	10.7007901495	0.3407315625
C	8.6596910596	12.8422571573	4.8950499145
C	4.2312783381	11.7324909523	5.5402178432
C	3.4485977709	6.2562495078	2.6512365991
C	2.5272422775	5.3791133431	2.0696531031
C	3.2825363356	12.7137542307	5.2647421752
C	2.2551675506	12.4178138108	4.3641242899
C	5.2224941643	5.2443259417	1.3791863032
C	9.7737494737	11.9795757537	7.9143192789
C	7.1264900716	5.5973898502	4.4108403624
C	8.6305762426	13.5300277130	3.6654460571
C	2.9292334434	6.0486300249	10.4818557250
C	4.3047355937	4.3675429937	0.7932853125
C	10.3322539728	9.5723494457	5.4655620147
C	8.2644823660	6.5043411447	2.4916902547
C	11.1517199919	7.5122591444	6.4478011909
C	10.0467729658	7.4993659602	7.3063760519
C	8.9602486139	13.3295688411	9.7578609063
C	7.6519937197	12.9737548601	9.4207438012
C	10.0209210457	12.8269568825	8.9984376931
C	1.1791405791	13.4406432489	4.0767931922
C	9.3988703142	5.7457616112	2.8070672083
C	8.2543215755	4.8393366325	4.7266762136
C	9.3983618741	4.9094638617	3.9238804041
H	5.1710758318	7.6092525239	0.2010447378
H	2.6811967957	5.4682179620	7.8036128241
H	5.6300145366	9.3449065100	8.4887398598
H	6.3897553161	11.8390572971	8.0773074106
H	8.0997781219	13.4839355491	1.5627089523

H	4.5764754470	8.2655631999	10.5079369799
H	5.6411577820	9.5681852735	-1.2067363109
H	3.8262635182	6.6769482286	5.9136797034
H	12.1437876254	8.5714126851	4.8454023222
H	2.2332047480	3.7420507029	0.6815282271
H	8.2362114539	8.5071082032	7.9155035038
H	3.2588450372	9.2427960427	3.6555904167
H	1.4564398017	10.8902048896	3.0437994280
H	6.8126588363	11.5729014907	-0.2892053693
H	9.0470953125	13.3589246186	5.7737220262
H	5.0625205300	11.9114166068	6.2241490185
H	3.1218582394	6.9880010858	3.3902157449
H	1.4720162502	5.4359098128	2.3516000227
H	3.3537051048	13.6933205629	5.7397134375
H	6.2778678809	5.1693419048	1.1099604708
H	10.6165208090	11.5858628851	7.3427165499
H	6.2340271029	5.5378145340	5.0359481807
H	9.0024726821	14.5569182860	3.6282383974
H	4.6534183923	3.6248026931	0.0706021788
H	10.4588111992	10.3771189626	4.7386446026
H	8.2849090513	7.1515586434	1.6125743867
H	11.8970523822	6.7141945791	6.4953239055
H	9.9272800753	6.6899036889	8.0317075179
H	9.1530057114	13.9878479759	10.6090173999
H	6.8125994794	13.3543014240	10.0096612357
H	11.0510612959	13.0886168585	9.2550765527
H	10.2876660183	5.8157500068	2.1741462413
H	8.2418243332	4.1884475519	5.6051913010
H	10.2837144601	4.3183550372	4.1717425513

Table S-6. Optimized XYZ coordinates for free pyridine (in Å)

C	-0.09347381560938	-1.44850688110984	0.01436418659670
C	1.09424836770535	-0.71083423724555	-0.03668986091683
C	1.00828577730467	0.68338408063671	-0.07039351548114
C	-0.25802361772095	1.27421383545630	-0.05178311926255
C	-1.38007086116299	0.44124644338284	-0.00066199139500
N	-1.30568327385567	-0.89118308964699	0.03304510845329
H	-0.06611165216097	-2.54591432704897	0.04272785174618
H	2.06156448677943	-1.22229554069261	-0.05098499181600
H	1.91260740879015	1.29888001187527	-0.11292187153426
H	-0.38173655070235	2.36172173739732	-0.07852378836596
H	-2.38960626936728	0.87228796699553	0.01482199197560

Table S-7. Optimized XYZ coordinates for free pyridine radical anion (in Å)

C	-0.08177975193811	-1.45307217803046	0.01357313633522

C	1.08860786969909	-0.72503946119141	-0.03193386505424
C	1.05081711275811	0.71181407742463	-0.05623025970838
C	-0.27358734769743	1.27449325849625	-0.04705146519345
C	-1.37999777276613	0.45314117999996	-0.00127710921395
N	-1.36173804778481	-0.92868066091617	0.03658409621668
H	-0.03301377690769	-2.55583107981979	0.03765115672404
H	2.04970797485846	-1.26203986167407	-0.04895159719703
H	1.95396563424345	1.32582876506301	-0.13179855127573
H	-0.42452209402550	2.36530493483860	-0.07705534735993
H	-2.38645980043944	0.90708102580946	0.00948980572676

X-Ray Diffraction Techniques. All structures were collected on a Rigaku Oxford Diffraction Synergy-S diffractometer equipped with a HyPix6000HE detector and operating with a CuKα (1.54184 Å) or MoKα (0.71073 Å) radiation source. Data collection, unit cell refinement, and data processing were carried out with CrysAlisPro, while structures were solved utilizing SHELXS and refined using SHELXL via Olex2. Olex2, PovRay, and ORTEP applications were used to generate structure graphics. Crystals were mounted on a cryoloop or glass fiber pin using Paratone N oil. Structures were collected at 100 K. All non-hydrogen atoms were refined anisotropically. Hydrogen atoms were placed at idealized positions and refined using a riding model. The isotropic displacement parameters of all hydrogen atoms were fixed to 1.2 times the atoms they are linked to (1.5 times for methyl groups). Further details on specific structures are noted below.

[Fe_2(^{Ph}Dbf)$_2$] (1a) The structure was solved in the monoclinic space group $P2_1/n$ with 4 molecules per unit cell. Two of the phenyl groups on the ligand exhibited disorder and were modeled using similarity constraints. Due to high disorder of the diethyl ether solvent molecules, an acceptable model could not be refined. Instead, a solvent mask was implemented in the Olex2 software which did not significantly affect the chemically significant features of the structure.

[Py_2Fe(^{Ph}Dbf)] (2a). The structure was solved in the monoclinic space group $P2_1/c$ with 4 molecules per unit cell. Benzene solvent molecules were modeled with 3 molecules in the unit cell.

[(*p*-^{t}Bu-Py)$_2$Fe(^{Ph}Dbf)] (3a). The structure was solved in the triclinic space group $P\bar{1}$ with 2 molecules per unit cell. The *p*-^{t}buPy functional group is clearly disordered, but attempts to model the disorder did not significantly improve the structure model. This group is surrounded by the solvent mask region and its position is presumably impacted by the positions of the disordered solvent. Thus the disorder is probably multiposition and/or dynamic. The data collection was set up assuming 2/m Laue symmetry when the correct Laue symmetry is -1. Considering this mistake, we are fortunate to have >99% completeness.

[(*p*-CF_3-Py)$_2$Fe(^{Ph}Dbf)] (4a). The structure was solved in the triclinic space group $P\bar{1}$ with 2 molecules per unit cell. Benzene solvent molecules were modeled with 2 molecules in the unit cell.

[(*p*-CF_3-Py)Fe_2(^{Ph}Dbf)$_2$] (5a). The structure was solved in the triclinic space group P$\bar{1}$ with 2 molecules per unit cell. Due to high disorder of the hexanes solvent molecules, an acceptable model could not be refined. Instead, a solvent mask was implemented in the Olex2 software which did not significantly affect the chemically significant features of the structure.

[PyFe(^{Ph}Dbf)Cl] (2b). The structure was solved in the triclinic space group P$\bar{1}$ with 2 molecules per unit cell. The Cl and pyridine ligands exhibited disorder and were modeled using similarity constraints.

[Py_2Fe(^{Ph}Dbf)][PF_6] (2c). The structure was solved in the triclinic space group P$\bar{1}$ with 2 molecules per unit cell. The PF_6^- anion exhibited disorder and was modeled using appropriate constraints. The trifluorotoluene solvent molecule exhibited disorder that was resolved by modeling the solvent in the asymmetric unit via the FragmentDB tool.

[(THF)$_2$Fe(^{Ph}Dbf)][PF_6] (7). The structure was solved in the triclinic space group P$\bar{1}$ with 4 molecules per unit cell. The crystal quality was relatively poor with disordered and partially evaporated solvent likely plaguing the sample. The poor crystal quality impacted the refinement by the need to use a solvent mask. In addition, a significant percentage of the ADPs are oblate or prolate. A disorder model was used for only the most extreme ADP cases; the rest were left as is resulting in quite a few checkCIF alerts related to ADPs.

Table S-8. X-ray diffraction experimental details

	$[Fe_2(^{Ph}Dbf)_2]$ (**1a**) CSD 2314720	$[Py_2Fe(^{Ph}Dbf)]$ (**2a**) CSD 2314721	$[(p\text{-}^{t}Bu\text{-}Py)_2Fe(^{Ph}Dbf)]$ (**3a**) CSD 2314723
**Empirical Formula**	$C_{76}H_{52}Fe_2O_6$	$C_{48}H_{36}FeN_2O_3$ $[1.5(C_6H_6)]$	$C_{56}H_{52}N_2O_3Fe$
**Formula Weight**	1172.87	744.67	856.84
**Temp. (K)**	100	100	100
**Radiation**	Mo	Cu	Cu
**Crystal System**	Monoclinic	Monoclinic	Triclinic
**Space Group**	$P2_1/n$	$P2_1/c$	$P\bar{1}$
**a (Å)**	23.4214(2)	13.1404(3)	11.8798(3)
**b (Å)**	9.87320(10)	20.3682(4)	14.6795(2)
**c (Å)**	31.4477(2)	17.0834(3)	15.0321(2)
**α (°)**	90	90	89.1050(10)
**β (°)**	104.3250(10)	105.160(2)	84.343(2)
**γ (°)**	90	90	89.289(2)
**Volume ($Å^3$)**	7045.99(11)	4413.19(16)	2608.20(8)
**Z**	4	4	2
**ρ_{calc} (g/cm^3)**	1.106	1.297	20.184
**μ (mm^{-1})**	3.672	0.391	6.104
**F(000)**	2432	18.4.0	16724
**Crystal size (mm^3)**	0.06 x 0.12 x 0.21	0.834 × 0.635 × 0.408	0.08 x 0.16 x 0.21
**2Θ range for data collection (°)**	5.4 – 155.12	6.514 – 76.436	6.078 – 76.502
**Index Ranges**	$-29 \leq h \leq 29$<br>$-12 \leq k \leq 11$<br>$-39 \leq l \leq 39$	$-22 \leq h \leq 22$<br>$-35 \leq k \leq 34$<br>$-29 \leq l \leq 28$	$-19 \leq h \leq 20$<br>$-24 \leq k \leq 24$<br>$-25 \leq l \leq 25$
**Reflections Collected**	182322	148057	82458
**Independent Reflections**	14909	23108	26641
**Data/Restraints/Parameters**	14909/0/767	23108/0/568	26641/0/565

**GOF**	1.052	1.029	1.049
**Final R indexes [I>=2σ (I)]**	R_1 = 0.0501, wR2 = 0.1356	R_1 = 0.0522, wR2 = 0.1395	R_1 = 0.0600, wR2 = 0.1706
**Final R indexes [all data]**	R_1 = 0.0551, wR2 = 0.1394	R_1 = 0.0651, wR2 = 0.1457	R_1 = 0.0827, wR2 = 0.1813
**Largest diff. peak/hole/e (Å^{-3})**	0.31/-0.60	0.89/-0.74	1.15/-1.23

Table S-8 continued. X-ray diffraction experimental details

	[(*p*-CF_3-Py)$_2$Fe(^{Ph}Dbf)] (**4a**) CSD 2314724	[(*p*-CF_3-Py)Fe_2(^{Ph}Dbf)$_2$] (**5a**) CSD 2314725	[Py_2Fe(^{Ph}Dbf)][PF_6] (**2b**) CSD 2314726
**Empirical Formula**	$C_{62}H_{46}F_6FeN_2O_3$•2(C_6H_6)	$C_{82}H_{56}F_3Fe_2NO_6$• [(C_6H_6)]•2.5[C_6H_{14}]	$C_{43}H_{31}ClFeNO_{36}$
**Formula Weight**	1036.86	1398.08	700.99
**Temp. (K)**	100	100	100
**Radiation**	Cu	Cu	Cu
**Crystal System**	Triclinic	Triclinic	Triclinic
**Space Group**	$P\bar{1}$	$P\bar{1}$	$P\bar{1}$
**a (Å)**	11.0504(2)	11.0908(7)	9.75483(18)
**b (Å)**	12.3691(2)	16.6402(9)	10.73958(17)
**c (Å)**	19.5705(2)	23.6934(9)	16.0247(2)
**α (°)**	73.374(2)	71.373(4)	77.2330(13)
**β (°)**	80.512(1)	83.723(4)	85.2312(14)
**γ (°)**	80.970(1)	89.424(5)	88.6981(14)
**Volume (Å^3)**	2510.95(7)	4117.4(4)	1631.60(5)
**Z**	2	2	2
**ρ_{calc} (g/cm^3)**	1.371	1.128	1.427
**μ (mm^{-1})**	3.015	3.276	4.806

**F(000)**	1072	1448	726.0
**Crystal size (mm^3)**	0.30 × 0.18 × 0.05	0.36 × 0.28 × 0.09	0.19 × 0.16 × 0.07
**2Θ range for data collection (°)**	4.75 – 55.028	5.606 – 127.198	5.674 – 155.02
**Index Ranges**	-13 ≤ h ≤ 13	-12 ≤ h ≤ 12	-12 ≤ h ≤ 12
	-15 ≤ k ≤ 15	-19 ≤ k ≤ 19	-13 ≤ k ≤ 13
	-24 ≤ l ≤ 24	-22 ≤ l ≤ 27	-20 ≤ l ≤ 20
**Reflections Collected**	53750	36287	29288
**Independent Reflections**	10562	12952	6881
**Data/Restraints/Parameters**	10562/0/667	12952/0/901	6881/0/506
**GOF**	1.103	1.092	1.114
**Final R indexes [I>=2σ (I)]**	R_1 = 0.0472, wR2 = 0.1327	R_1 = 0.0699, wR2 = 0.1881	R_1 = 0.0491, wR2 = 0.1495
**Final R indexes [all data]**	R_1 = 0.0529, wR2 = 0.1374	R_1 = 0.1053, wR2 = 0.2074	R_1 = 0.0515, wR2 = 0.1520
**Largest diff. peak/hole/e ($Å^{-3}$)**	0.64/-0.58	0.58/-0.49	0.52/-0.79

Table S-8 continued. X-ray diffraction experimental details

	$[Py_2Fe(^{Ph}Dbf)][PF_6]$ (**2c**) CSD 2314727	$[(THF)_2Fe(^{Ph}Dbf)][PF_6]$ (**7**) CSD 2314729
**Empirical Formula**	$2[C_{48}H_{36}FeN_2O_3PF_6]\bullet[(C_7H_5F_3)]$	$C_{59.5}H_{68.5}F_7FeO_5P$
**Formula Weight**	21925.32	1083.46
**Temp. (K)**	100	100
**Radiation**	Cu	Cu
**Crystal System**	Triclinic	Triclinic
**Space Group**	$P\bar{1}$	$P\bar{1}$
**a (Å)**	11.4861(1)	10.4348(2)

**b (Å)**	12.0461(2)	19.4122(4)
**c (Å)**	16.8315(3)	25.4468(4)
**α (°)**	79.702(2)	85.1350(10)
**β (°)**	82.423(1)	86.701(2)
**γ (°)**	72.767(1)	78.805(2)
**Volume ($Å^3$)**	2180.75(6)	5033.82(17)
**Z**	2	4
**ρ_{calc} (g/cm^3)**	1.466	1.430
**μ (mm^{-1})**	3.809	3.360
**F(000)**	988.0	2278.0
**Crystal size (mm^3)**	0.241 × 0.143 × 0.068	0.46 × 0.09 × 0.05
**2Θ range for data collection (°)**	2.678 – 77.509	4.654 – 156.682
**Index Ranges**	$-14 \le h \le 13$<br>$-15 \le k \le 15$<br>$-21 \le l \le 21$	$-13 \le h \le 12$<br>$-24 \le k \le 24$<br>$-32 \le l \le 30$
**Reflections Collected**	40086	116206
**Independent Reflections**	9199	21079
**Data/Restraints/Parameters**	9199/0/695	21079/734/1229
**GOF**	1.1	1.070
**Final R indexes [I>=2σ (I)]**	R_1 = 0.0396,<br>wR2 = 0.1115	R_1 = 0.0775,<br>wR_2 = 0.2288
**Final R indexes [all data]**	R_1 = 0.0416,<br>wR2 = 0.1133	R_1 = 0.0968,<br>wR_2 = 0.2495
**Largest diff. peak/hole/e ($Å^{-3}$)**	0.44/-0.54	1.01/-0.72

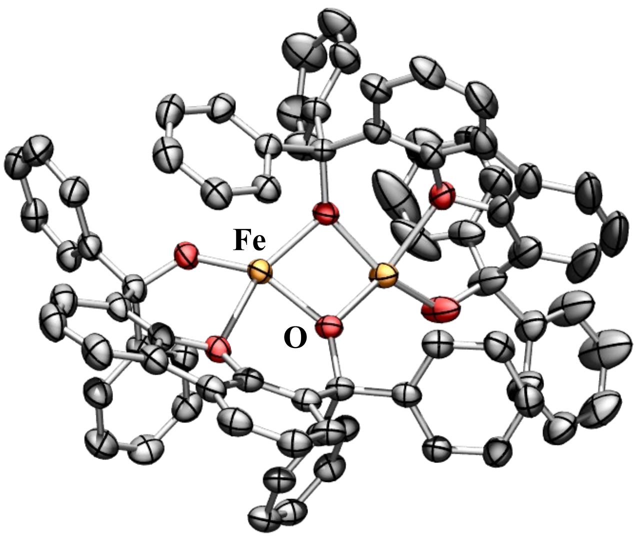

Figure S-72. Solid-state molecular structure for $[Fe_2(^{Ph}Dbf)_2]$ (**1a**) with anisotropic displacement ellipsoids at 50% probability level. Hydrogen atoms and disordered phenyl groups are omitted for clarity. Color scheme: Fe – orange, O – red, C – gray.

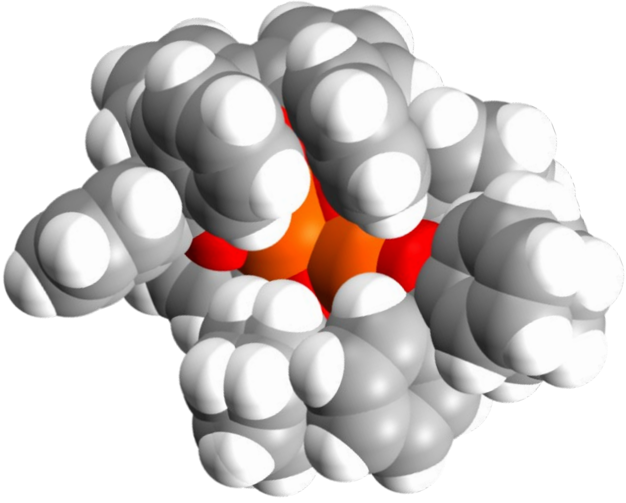

Figure S-73. Space-filling model of the solid-state molecular structure for $[Fe_2(^{Ph}Dbf)_2]$ (**1a**) Color scheme: Fe – orange, O – red, C – gray, H – white.

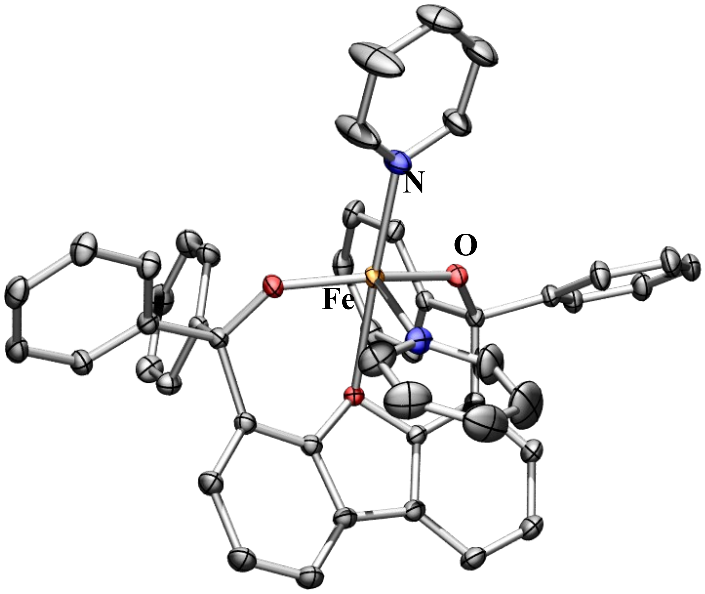

Figure S-74. Solid-state molecular structure for [$Py_2Fe(^{Ph}Dbf)$] (**2a**) with anisotropic displacement ellipsoids at 50% probability level. Hydrogen atoms are omitted for clarity. Color scheme: Fe – orange, O – red, N – blue, C – gray.

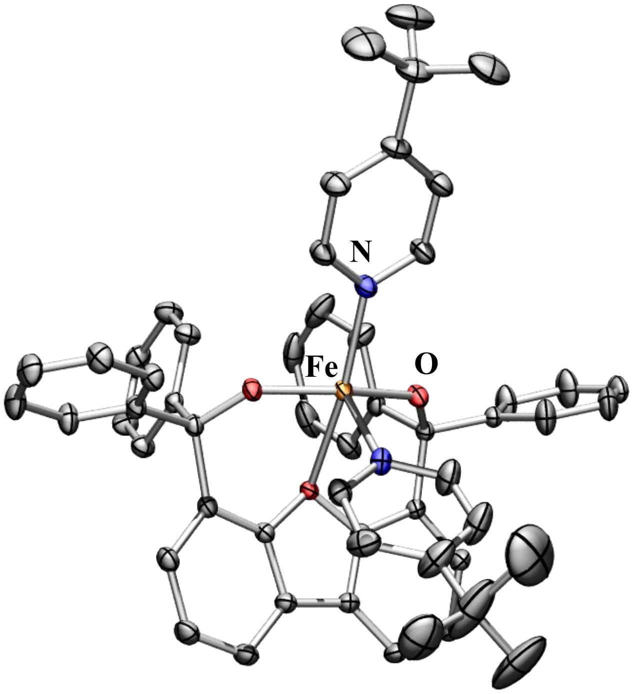

Figure S-75. Solid-state molecular structure for [(p-^{t}Bu-Py)$_2$Fe(^{Ph}Dbf)] (**3a**) with anisotropic displacement ellipsoids at 50% probability level. Hydrogen atoms are omitted for clarity. Color scheme: Fe – orange, O – red, N – blue, C – gray.

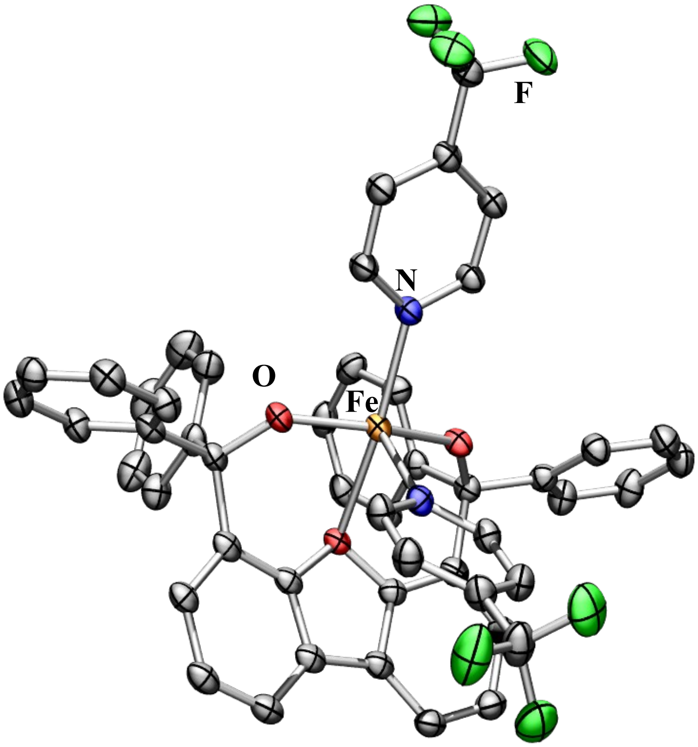

Figure S-76. Solid-state molecular structure for [(*p*-CF_3-Py)$_2$Fe(^{Ph}Dbf)] (**4a**) with anisotropic displacement ellipsoids at 50% probability level. Hydrogen atoms and benzene solvent in the unit cell are omitted for clarity. Color scheme: Fe – orange, O – red, N – blue, C – gray, F – green.

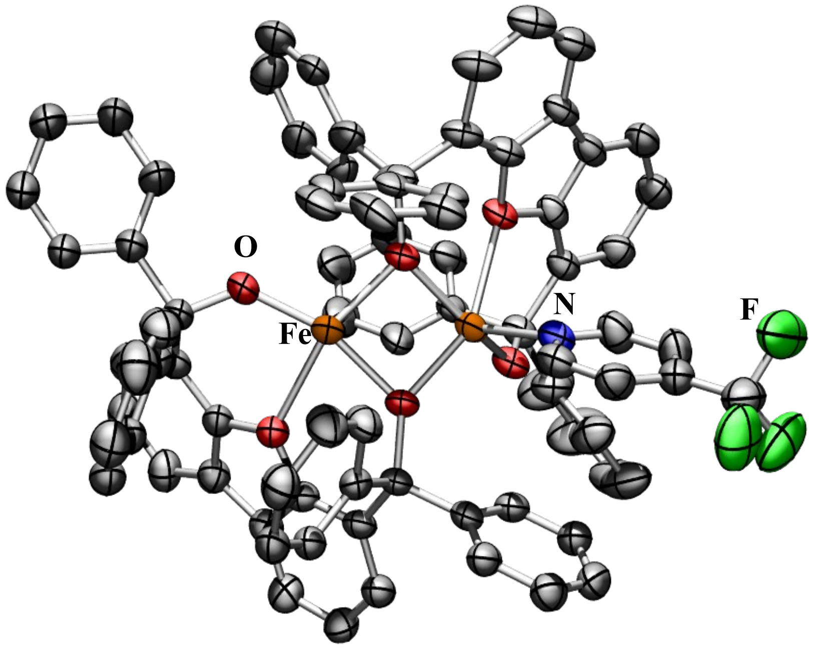

Figure S-77. Solid-state molecular structure for [(p-CF_3-Py)Fe_2(^{Ph}Dbf)$_2$] (**5a**) with anisotropic displacement ellipsoids at 50% probability level. Hydrogen atoms and benzene solvent in the unit cell are omitted for clarity. Color scheme: Fe – orange, O – red, N – blue, C – gray, F – green.

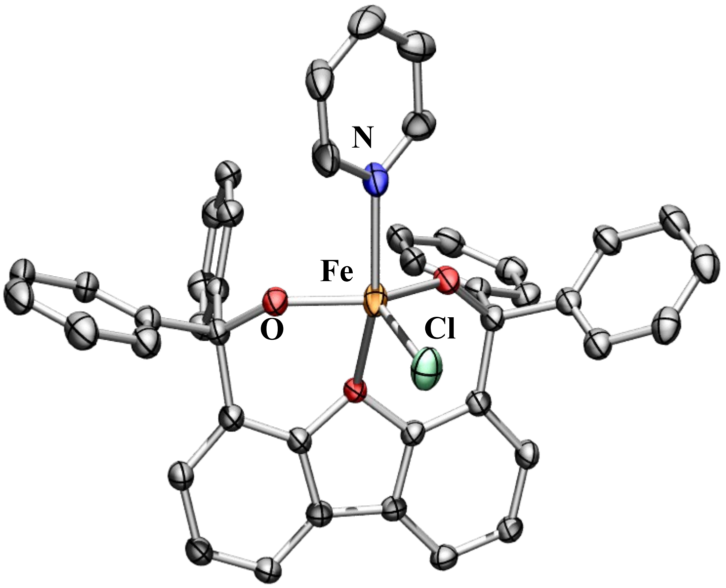

Figure S-78. Solid-state molecular structure for [PyFe(^{Ph}Dbf)Cl] (**2b**) with anisotropic displacement ellipsoids at 50% probability level. Hydrogen atoms and disordered pyridine and chloride groups are omitted for clarity. Color scheme: Fe – orange, O – red, N – blue, C – gray, Cl – aquamarine.

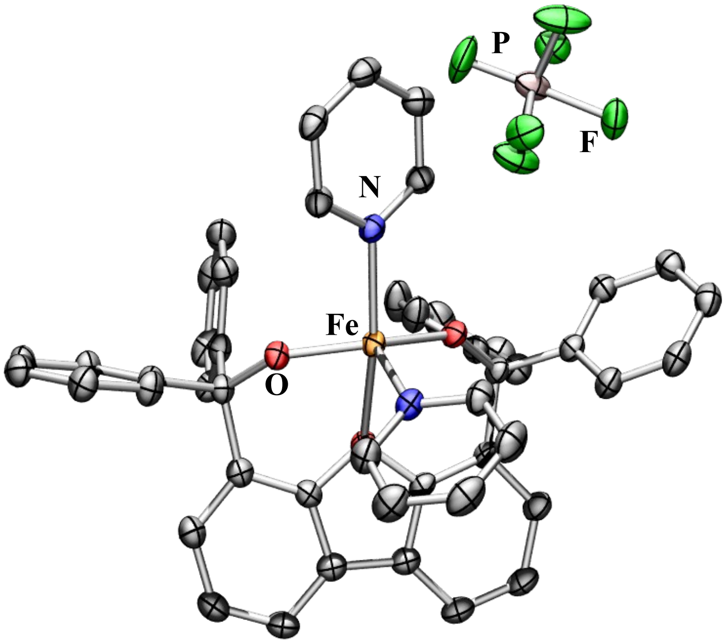

Figure S-79. Solid-state molecular structure for $[Py_2Fe(^{Ph}Dbf)][PF_6]$ (**2c**) with anisotropic displacement ellipsoids at 50% probability level. Hydrogen atoms and disordered hexafluorophosphate group are omitted for clarity. Color scheme: Fe – orange, O – red, N – blue, C – gray, F – green, P – pink.

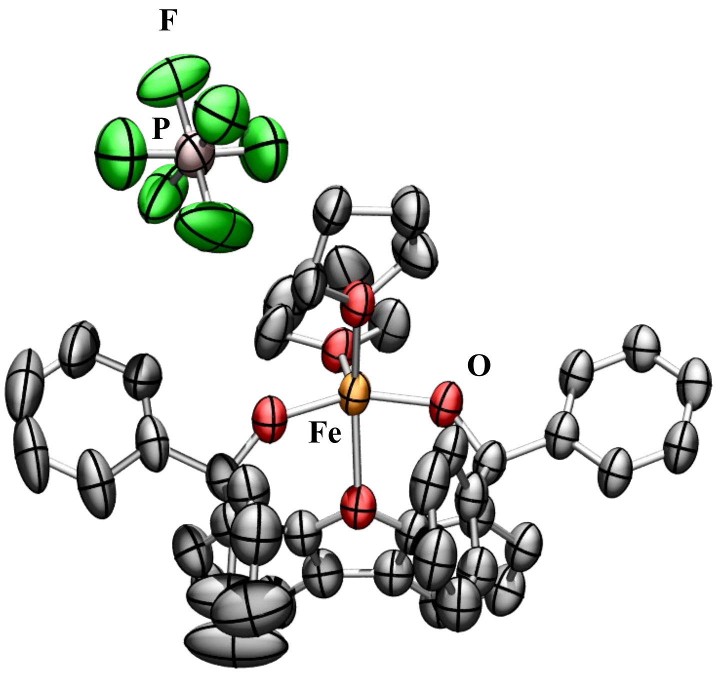

Figure S-80. Solid-state molecular structure for [(THF)$_2$Fe(^{Ph}Dbf)][PF$_6$] (**7**) with anisotropic displacement ellipsoids at 50% probability level. Hydrogen atoms and disordered hexafluorophosphate and THF groups are omitted for clarity. Color scheme: Fe – orange, O – red, C – gray, F – green, P – pink.

Table S-9. Relevant bond metrics for $[Fe_2(^{Ph}Dbf)_2]$ (**1a**)

Complex **1a**	Bond Length (Å)	Bond Angle (◦)
Fe_1-O_1	1.8541(15)	–
Fe_2-O_2	1.8516(17)	–
Fe_1-O_3 (μ-O)	2.0062(14)	–
Fe_2-O_3 (μ-O)	1.9947(14)	–
Fe_1-O_4 (μ-O)	2.0211(14)	–
Fe_2-O_4 (μ-O)	1.9620(14)	–
Fe_1-O_5 (O_{furan})	2.2230(13)	–
Fe_2-O_6 (O_{furan})	2.2243(16)	–
Fe_1-O_3-Fe_2	–	95.56(6)
Fe_1-O_3-Fe_2	–	97.12(6)
O_1-Fe_1-O_4	–	120.80(6)
O_3-Fe_2-O_2	–	121.67(6)
O_5-Fe_1-O_3	–	80.51(5)
O_4-Fe_2-O_6	–	81.00(5)

Table S-10. Reported g_{eff} values obtained via low temperature (80 K) EPR for Fe(III) species

Complex	ID Number	g_{eff} Values
$[Fe_2(^{Ph}Dbf)_2(Cl)_2]$	1b	9.28, 4.46, 4.22
$[Fe(^{Ph}Dbf)(Py)(Cl)]$	2b	9.35, 4.58, 4.18
$[Fe(^{Ph}Dbf)(p\text{-}^{t}Bu\text{-}Py)(Cl)]$	3b	9.24, 4.59, 4.16
$[Fe(^{Ph}Dbf)(p\text{-}CF_3\text{-}Py)(Cl)]$	4b	9.09, 4.56, 4.24
$[Fe_2(^{Ph}Dbf)_2(p\text{-}CF3\text{-}Py)(Cl)_2]$	5b	9.74, 4.44, 4.24
$[Fe_2(^{Ph}Dbf)_2(m\text{-}CF3\text{-}Py)(Cl)_2]$	6b	9.5, 4.52, 4.25
$[Fe_2(^{Ph}Dbf)_2][PF_6]$	1c	–
$[Fe(^{Ph}Dbf)(Py)_2][PF_6]$	2c	9.2, 5.0, 4.27
$[Fe(^{Ph}Dbf)(p\text{-}^{t}Bu\text{-}Py)_2][PF_6]$	3c	9.13, 4.25
$[Fe(^{Ph}Dbf)(p\text{-}CF_3\text{-}Py)_2][PF_6]$	4c	9.23, 4.28
$[Fe_2(^{Ph}Dbf)_2(p\text{-}CF3\text{-}Py)][PF_6]$	5c	–
$[Fe_2(^{Ph}Dbf)_2(m\text{-}CF3\text{-}Py)][PF_6]$	6c	–

Species 1c, 5c, and 6c decomposed rapidly and largely organic radical was observed in the EPR spectra. Please see S-49 through SI-52 for more information.

Pyridine Lability Studies

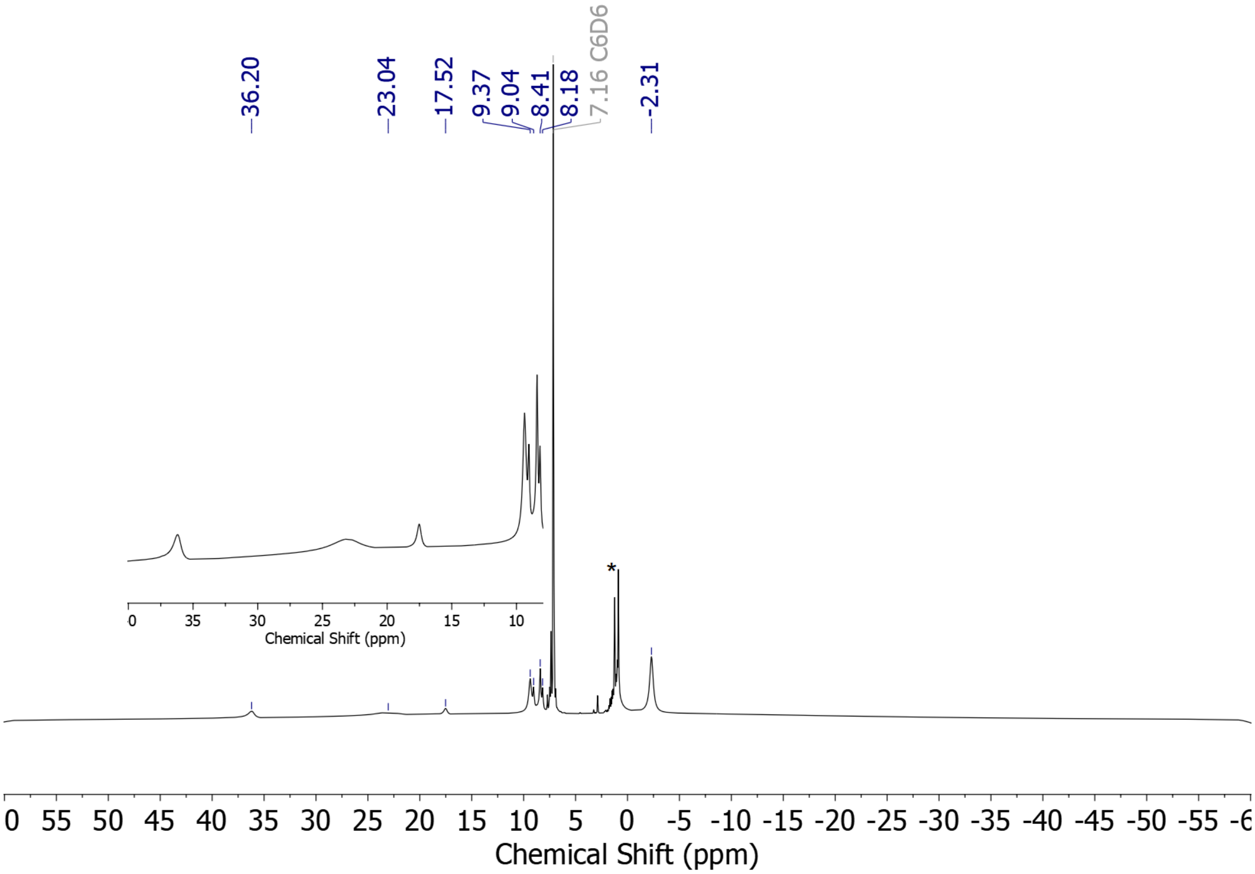

Figure S-81. ^{1}H NMR of the isolated product (yellow) of the reaction of [Fe(^{Ph}Dbf)(Py)$_2$] (**2a**) and 4-*tert*-butylpyridine. The isolated product can be identified as the corresponding pyridine bound monomer [Fe(^{Ph}Dbf)(*p*-^{t}Bu-Py)$_2$] (**3a**). * indicated minor organic impurity from trituration (hexanes).

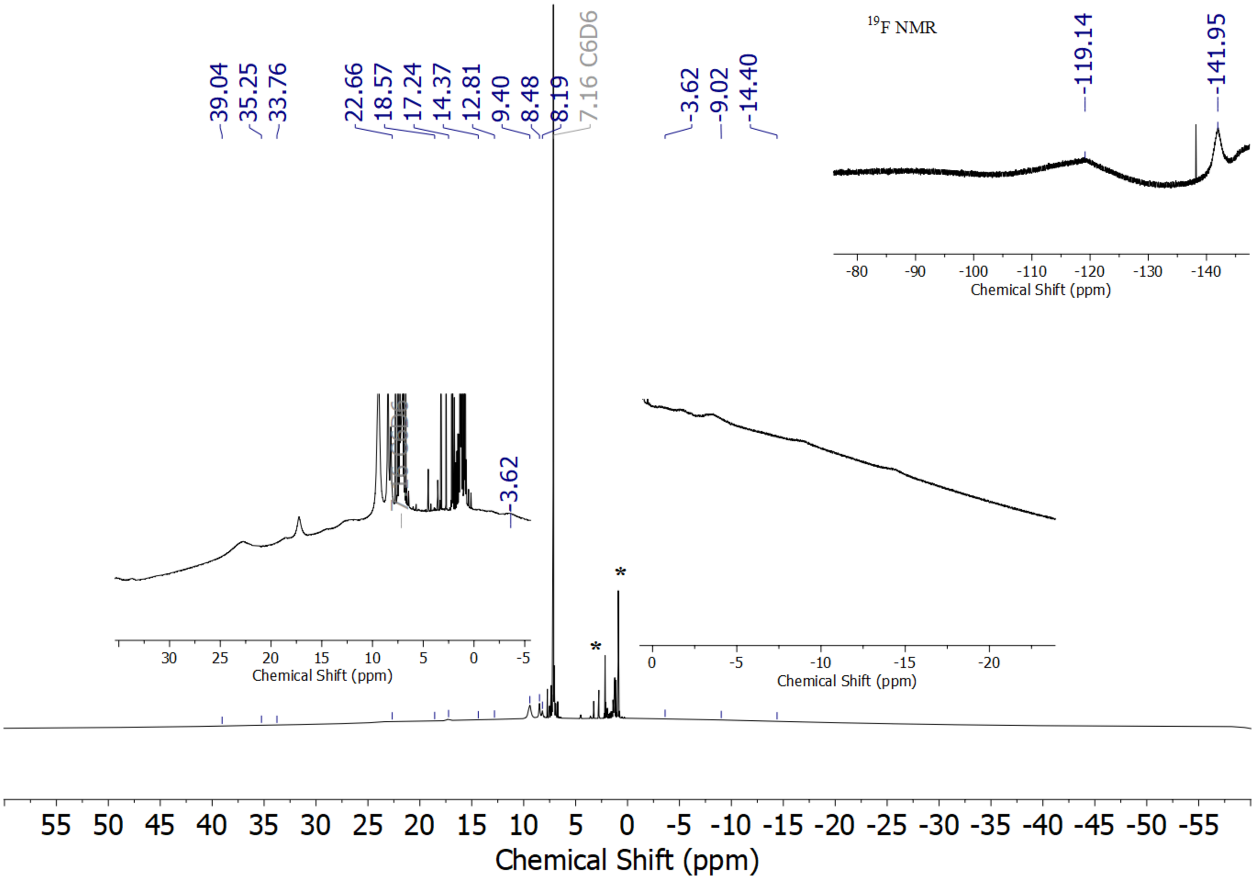

Figure S-82. ^{1}H NMR of the isolated product (purple) of the reaction of [Fe(^{Ph}Dbf)(Py)$_2$] (**2a**) and 4-trifluoromethylpyridine. The isolated product can be identified as the corresponding pyridine bound monomer [Fe(^{Ph}Dbf)(*p*-CF_3-Py)$_2$] (**4a**) and [Fe_2(^{Ph}Dbf)$_2$(*p*-CF_3-Py)] (**5a**). The inlaid (*left and right bottom*) ^{1}H NMR highlights identifying paramagnetic peaks. The inlaid ^{19}F NMR (*right top*) indicates formation of [Fe(^{Ph}Dbf)(*p*-CF_3-Py)$_2$] (**4a**) and [Fe_2(^{Ph}Dbf)$_2$(*p*-CF_3-Py)] (**5a**) and free *p*-CF_3-Py. * indicates minor organic impurities (hexanes and pyridine). Impurities with signals near the residual benzene peak (pyridine) were not labeled for clarity.

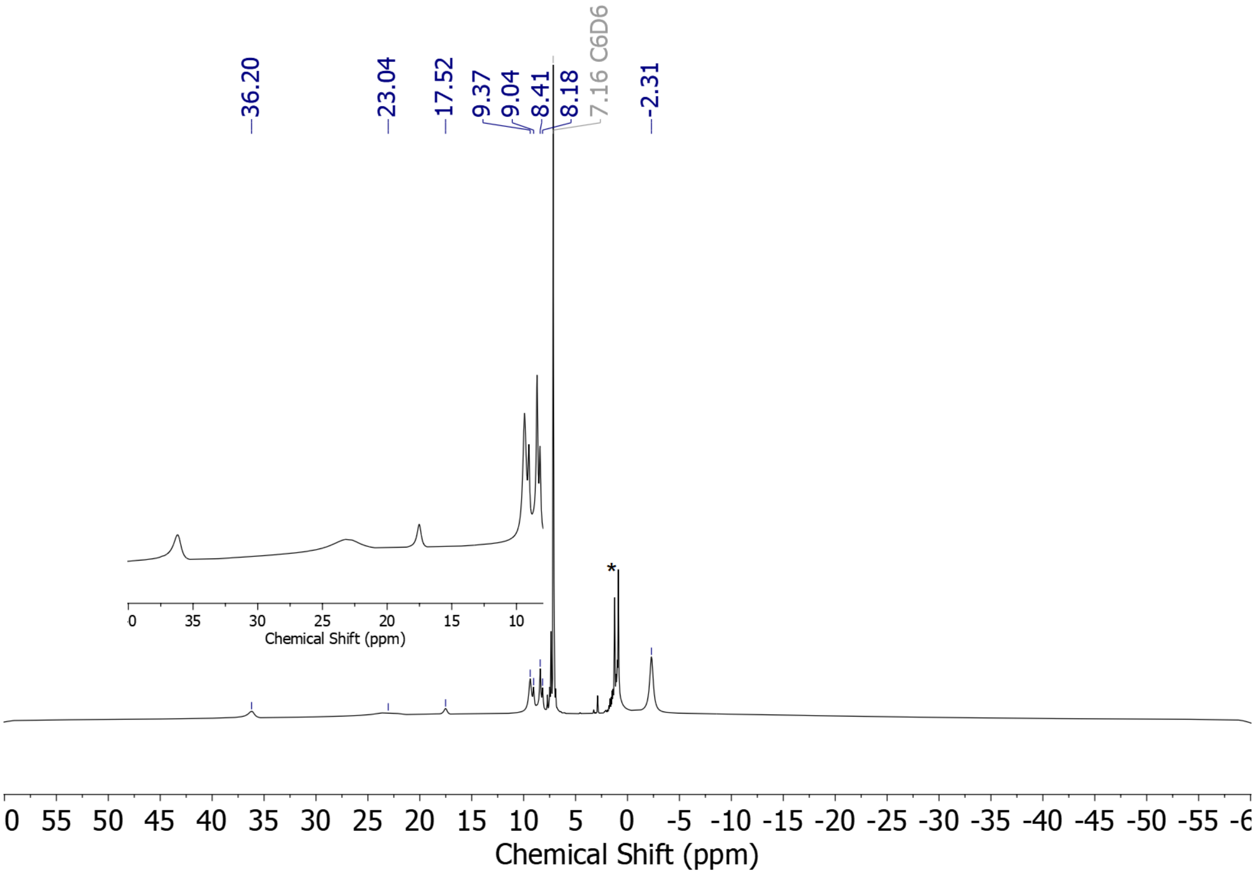

Figure S-83. ^{1}H NMR of the isolated product (yellow) of the reaction of [Fe(^{Ph}Dbf)(*p*-^{t}Bu-Py)$_2$] (**3a**) and pyridine. The isolated product can be identified as [Fe(^{Ph}Dbf)(*p*-^{t}Bu-Py)$_2$] (**3a**), suggesting that pyridine cannot displace *p*-*tert*-Butyl-pyridine. The inlaid spectra highlights identifying paramagnetic peaks at high chemical shifts. * indicates minor organic impurities from hexanes.

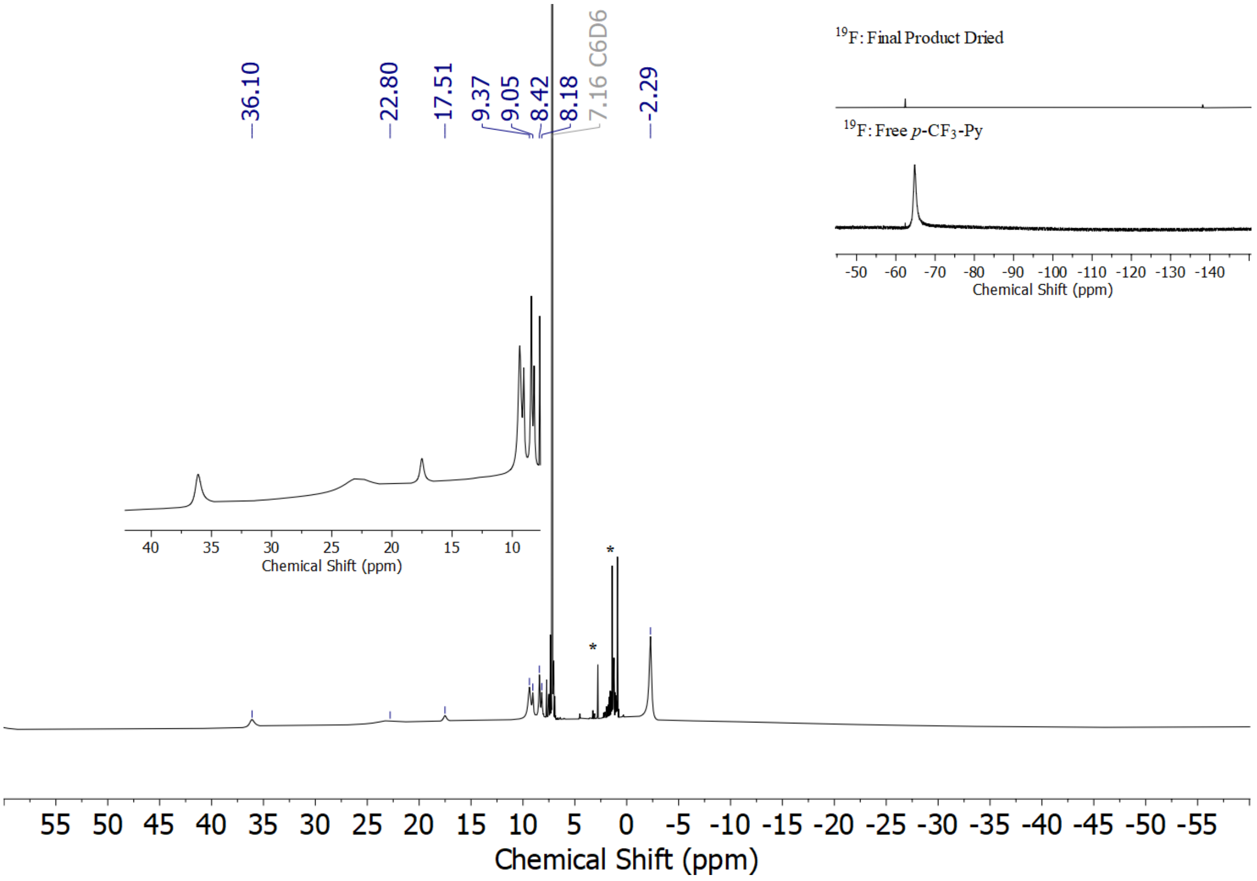

Figure S-84. ^{1}H NMR of the isolated product (yellow) of the reaction of [Fe(^{Ph}Dbf)(*p*-^{t}Bu-Py)$_2$] (**3a**) and 4-trifluoromethylpyridine. The isolated product can be identified as [Fe(^{Ph}Dbf)(*p*-^{t}Bu-Py)$_2$] (**3a**), suggesting that 4-trifluoromethylpyridine cannot displace *p*-*tert*-Butyl-pyridine. The inlaid spectra highlights identifying paramagnetic peaks at high chemical shifts. * indicates minor organic impurities from hexanes. Peaks associated with excess 4-trifluoromethylpyridine around the residual benzene peak were not labeled for clarity.

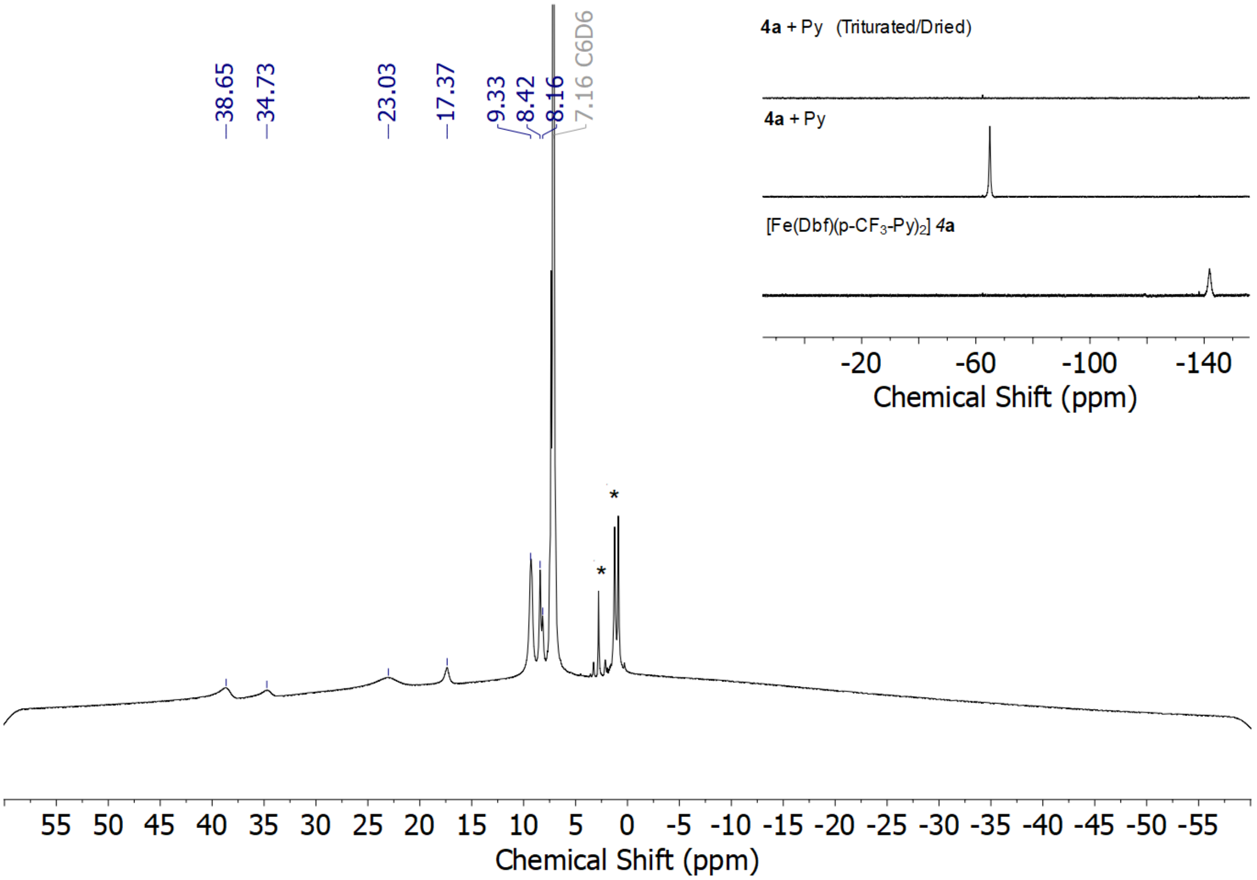

Figure S-85. ^{1}H NMR of the isolated product (orange) of the reaction of [Fe(^{Ph}Dbf)(*p*-CF_3-Py)$_2$] (**4a**) and pyridine. The isolated product can be identified as the corresponding pyridine bound monomer [Fe(^{Ph}Dbf)(Py)$_2$] (**2a**). The inlet stacked ^{19}F NMR spectra are of [Fe(^{Ph}Dbf)(*p*-CF_3-Py)$_2$] (**4a**) (*inlet bottom*), the reaction of [Fe(^{Ph}Dbf)(*p*-CF_3-Py)$_2$] (**4a**) with pyridine both in solution (*inlet middle*) and hexanes trituration and drying (*inlet top*). The signal in the organic region (*inlet middle*) indicates free *p*-CF_3-Py upon addition of pyridine ligand. Additionally, this free *m*-CF_3-Py can be removed to isolate the corresponding pyridine monomer [Fe(^{Ph}Dbf)(Py)$_2$] (**2a**) as seen by the ^{1}H NMR and ^{19}F NMR (*inlet top*). * indicates minor organic impurities (hexanes and pyridines). Impurities with signals near the residual benzene peak (pyridines) were not labeled for clarity.

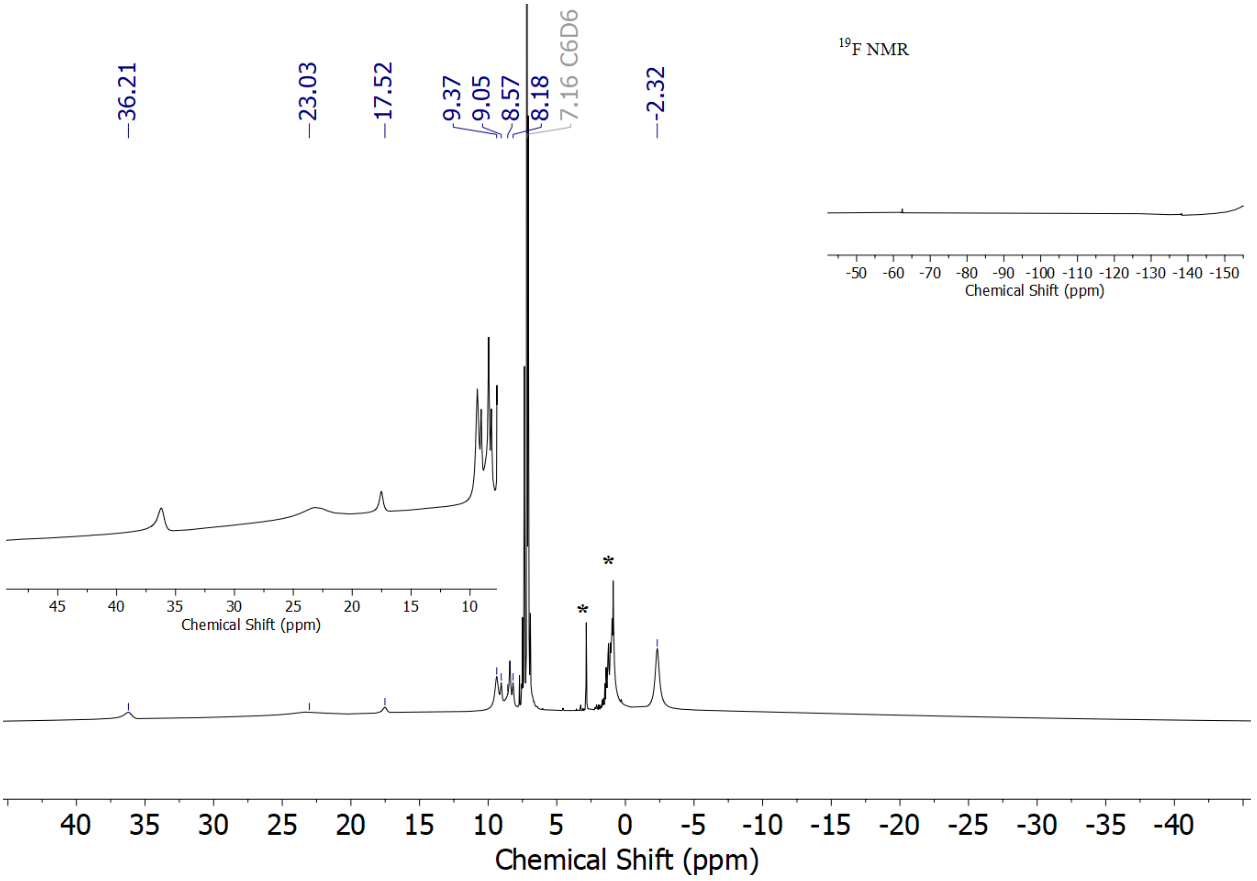

Figure S-86. ^{1}H NMR of the isolated product (orange) of the reaction of [Fe(^{Ph}Dbf)(*p*-CF_3-Py)$_2$] (**4a**) and 4-*tert*-butylpyridine. The isolated product can be identified as the corresponding pyridine bound monomer [Fe(^{Ph}Dbf)(*p*-^{t}Bu-Py)$_2$] (**3a**). The inlaid (*left*) ^{1}H NMR highlights identifying paramagnetic peaks. The ^{19}F NMR (*inlay right*) indicates free *p*-CF_3-Py can be removed by vacuum to isolate the corresponding monomer [Fe(^{Ph}Dbf)(*p*-^{t}Bu-Py)$_2$] (**3a**) as seen by the ^{1}H NMR and ^{19}F NMR (*inlet top*). * indicates minor organic impurities (hexanes and ligands). Impurities with signals near the residual benzene peak (pyridines) were not labeled for clarity.

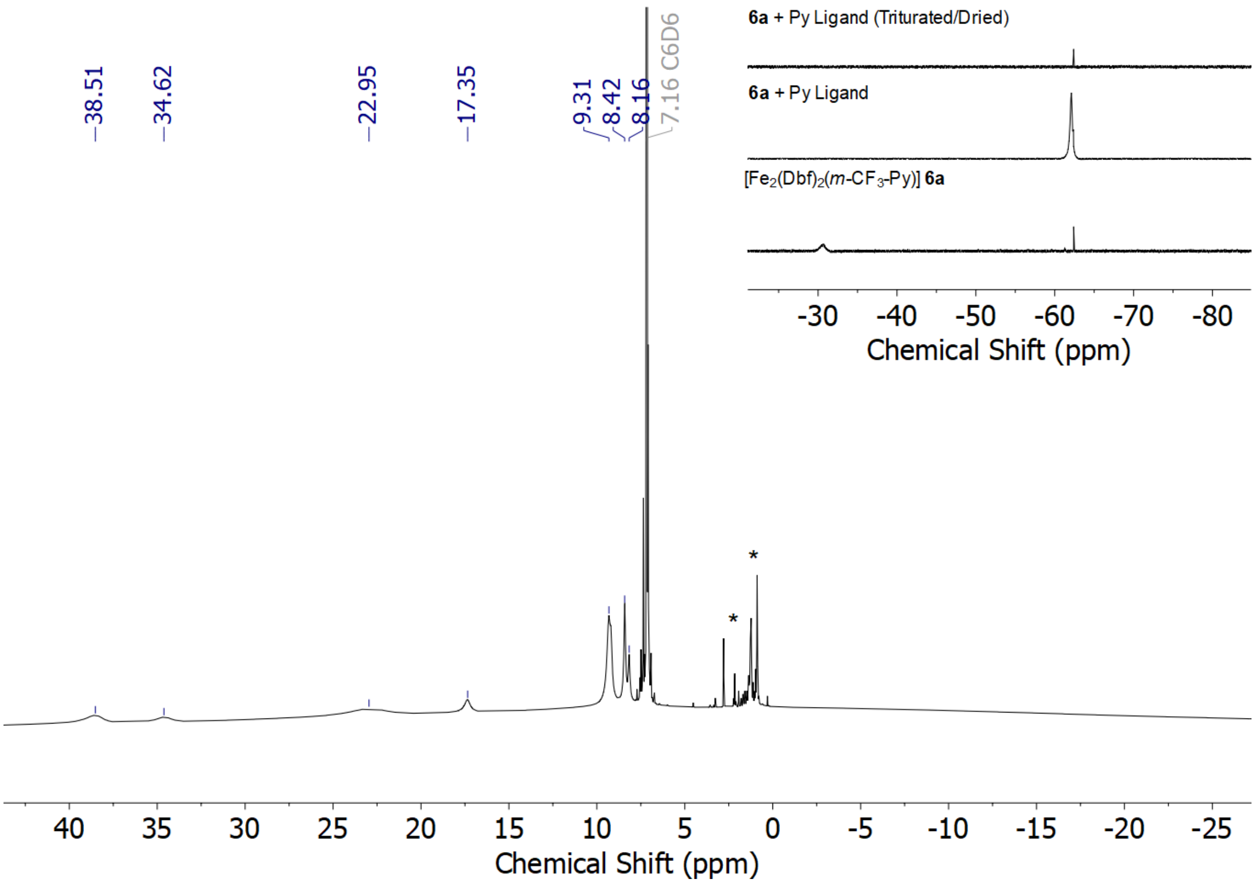

Figure S-87. ^{1}H NMR of the isolated product (orange) of the reaction of [Fe_2(^{Ph}Dbf)$_2$(*m*-CF_3-Py)] (**6a**) and pyridine. The isolated product can be identified as the corresponding pyridine bound monomer [Fe(^{Ph}Dbf)(Py)$_2$] (**2a**). The inlet stacked ^{19}F NMR spectra are of [Fe_2(^{Ph}Dbf)$_2$(*m*-CF_3-Py)] (**6a**) (*inlet bottom*), the reaction of [Fe_2(^{Ph}Dbf)$_2$(*m*-CF_3-Py)] (**6a**) with pyridine both in solution (*inlet middle*) and hexanes trituration and drying (*inlet top*). The signal in the organic region (*inlet middle*) indicates free *m*-CF_3-Py upon addition of pyridine ligand. Additionally, this free *m*-CF_3-Py can be removed to isolate the corresponding pyridine monomer [Fe(^{Ph}Dbf)(Py)$_2$] (**2a**) as seen by the ^{1}H NMR and ^{19}F NMR (*inlet top*). * indicates minor organic impurities (hexanes and ligands). Impurities with signals near the residual benzene peak (pyridines) were not labeled for clarity.

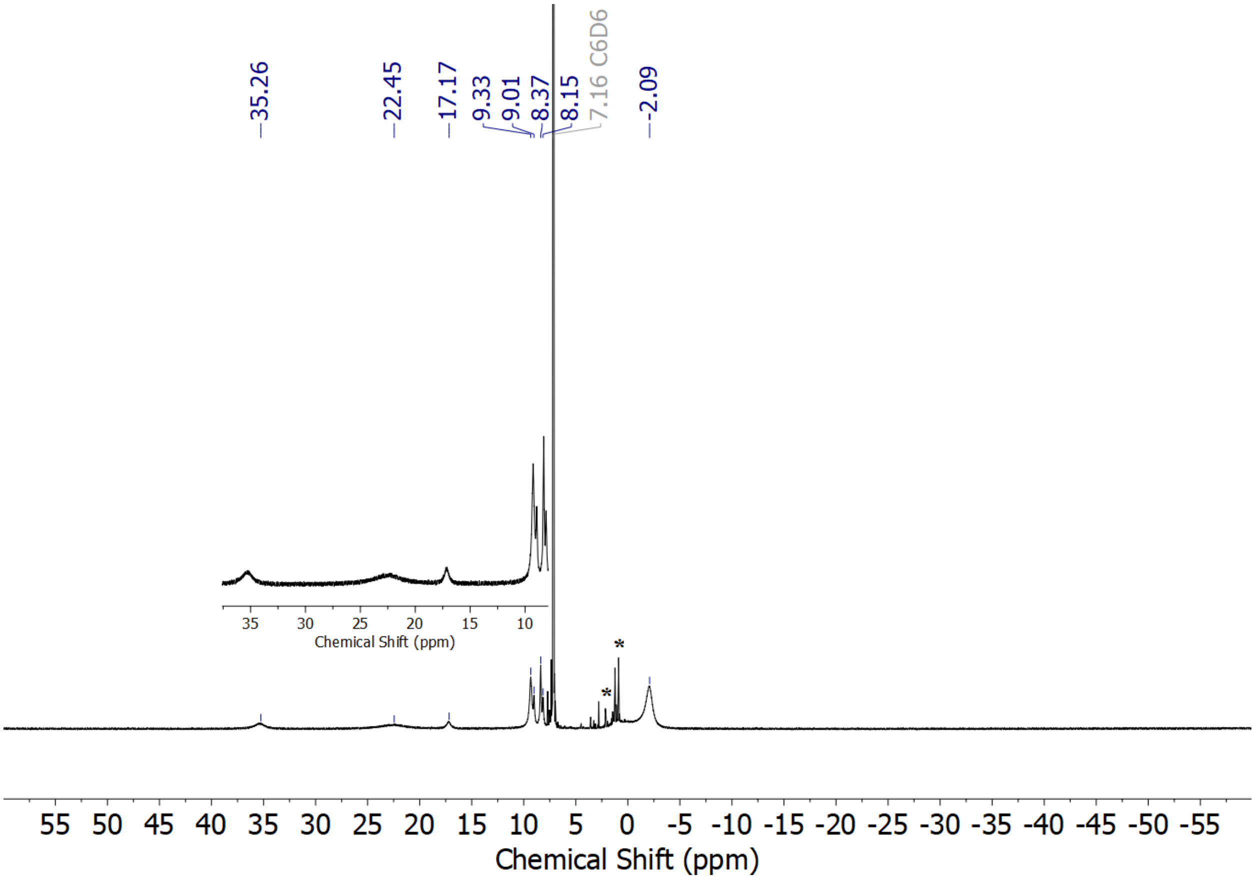

Figure S-88. ^{1}H NMR of the isolated product (orange) of the reaction of [Fe_2(^{Ph}Dbf)$_2$(*m*-CF_3-Py)] (**6a**) and 4-*tert*-butylpyridine. The isolated product can be identified as the corresponding pyridine bound monomer [Fe(^{Ph}Dbf)(*p*-^{t}Bu-Py)$_2$] (**3a**). The inlaid (*left*) ^{1}H NMR highlights identifying paramagnetic peaks. * indicates minor organic impurities (hexanes and pyridine). Impurities with signals near the residual benzene peak (pyridine) were not labeled for clarity.

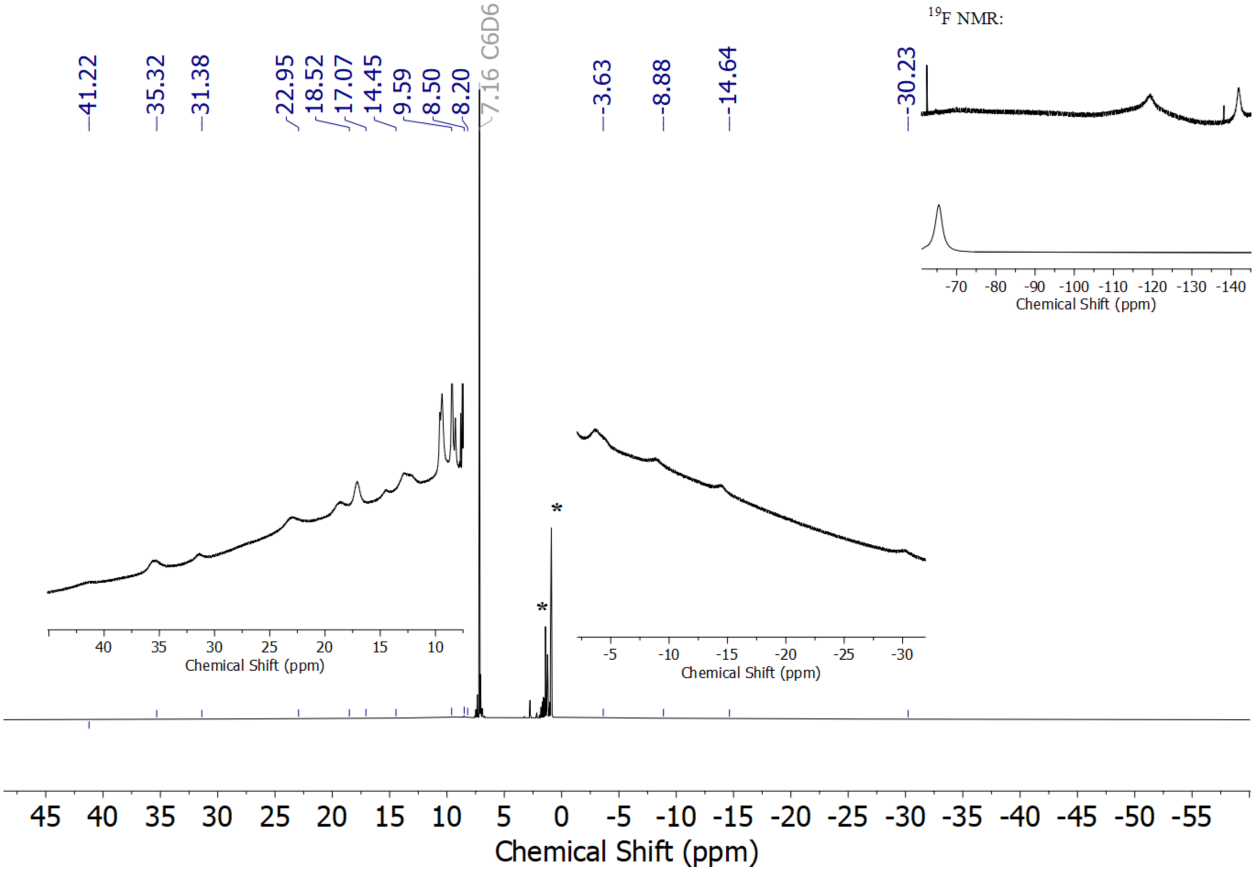

Figure S-89. ^{1}H NMR of the isolated product (orange) of the reaction of $[Fe_2(^{Ph}Dbf)_2(m\text{-}CF_3\text{-}Py)]$ (**6a**) and 4-trifluoromethylpyridine. The isolated product can be identified as the corresponding pyridine bound monomer $[Fe(^{Ph}Dbf)(p\text{-}CF_3\text{-}Py)_2]$ (**4a**) and $[Fe_2(^{Ph}Dbf)_2(p\text{-}CF_3\text{-}Py)]$ (**5a**). The inlaid (*left and right bottom*) ^{1}H NMR highlights identifying paramagnetic peaks. The inlaid ^{19}F NMR (*right top*) indicates formation of $[Fe(^{Ph}Dbf)(p\text{-}CF_3\text{-}Py)_2]$ (**4a**) and $[Fe_2(^{Ph}Dbf)_2(p\text{-}CF_3\text{-}Py)]$ (**5a**) and free *p*-CF_3-Py. * indicates minor organic impurities (hexanes and pyridine). Impurities with signals near the residual benzene peak (pyridine) were not labeled for clarity.

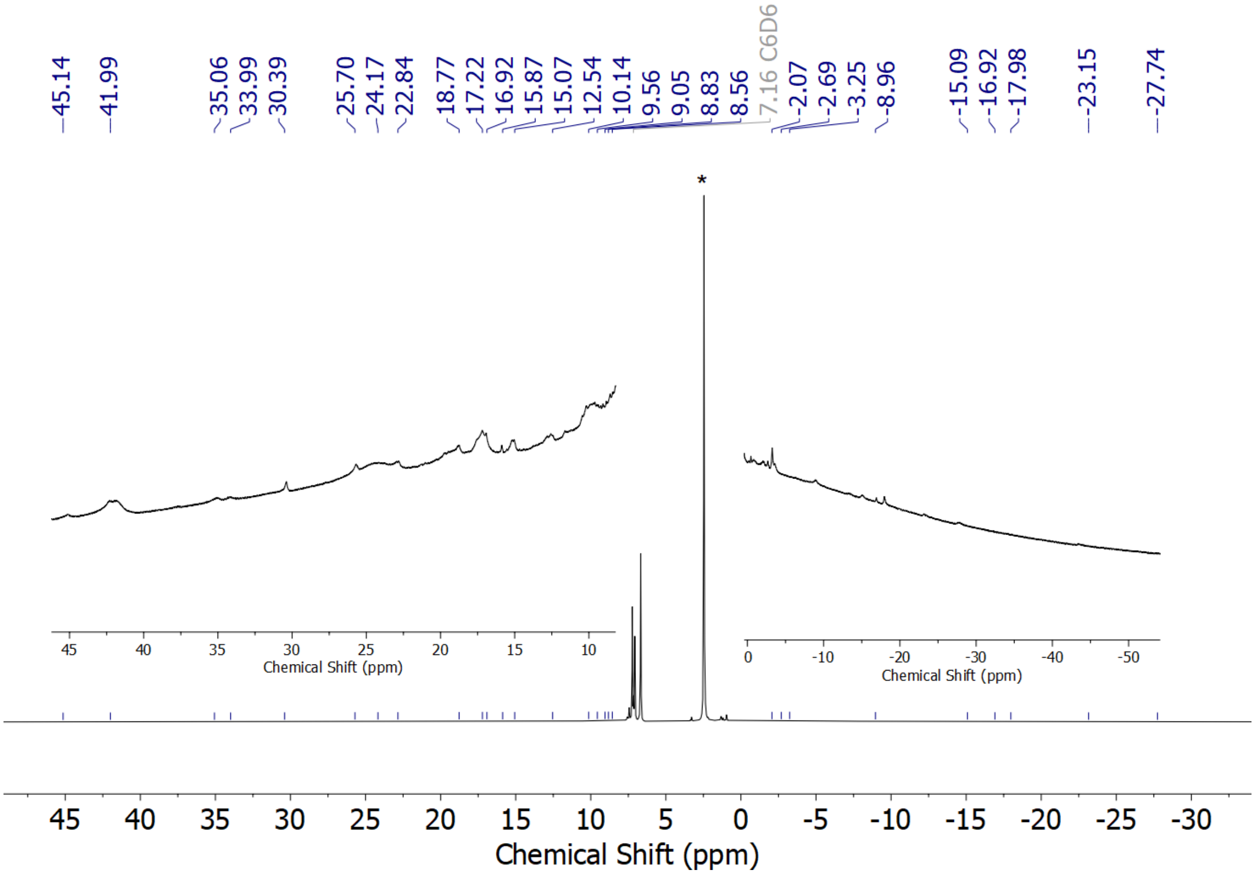

Figure S-90. ^{1}H NMR of the product of the reaction of [$Fe_2(^{Ph}Dbf)_2$] (**1a**) and lutidine. The inlaid (*left and right bottom*) ^{1}H NMR highlights identifying paramagnetic peaks. * indicates corresponds to the methyl group in lutidine from the excess lutidine added.
